# Secondary Metabolites of the Marine Sponge-Derived Fungus *Aspergillus subramanianii* 1901NT-1.40.2 and Their Antimicrobial and Anticancer Activities

**DOI:** 10.3390/md23090353

**Published:** 2025-08-30

**Authors:** Olga O. Khmel, Anton N. Yurchenko, Phan Thi Hoai Trinh, Ngo Thi Duy Ngoc, Vo Thi Dieu Trang, Huynh Hoang Nhu Khanh, Alexandr S. Antonov, Konstantin A. Drozdov, Roman S. Popov, Natalya Y. Kim, Dmitrii V. Berdyshev, Ekaterina A. Chingizova, Ekaterina S. Menchinskaya, Ekaterina A. Yurchenko

**Affiliations:** 1 G.B. Elyakov Pacific Institute of Bioorganic Chemistry Far Eastern Branch of Russian Academy of Science, Prospect 100-letiya Vladivostoka, 159, Vladivostok 690022, Russia; khmel.oo@dvfu.ru (O.O.K.); yurchenkoan@piboc.dvo.ru (A.N.Y.); antonov_as@piboc.dvo.ru (A.S.A.); popov_rs@piboc.dvo.ru (R.S.P.); kim_ny@piboc.dvo.ru (N.Y.K.); chingizova_ea@piboc.dvo.ru (E.A.C.);; 2 Institute of Biotechnology, Bioengineering and Food Systems, Advanced Engineering School, Far Eastern Federal University, 10 Ajax Bay, Russky Island, Vladivostok 690922, Russia; 3 Institute of Oceanography, Vietnam Academy of Science and Technology, Nha Trang 650000, Vietnam; phanhoaitrinh84@gmail.com (P.T.H.T.); votrang@io.vast.vn (V.T.D.T.); khanhhuynh@io.vast.vn (H.H.N.K.)

**Keywords:** *Aspergillus subramanianii*, *Circumdati*, secondary metabolites, ergostanoid, pyrazine alkaloid, bis-indolyl benzenoid alkaloids, *Candida albicans*, anticancer activity, breast cancer

## Abstract

The aim of this study was to investigate the metabolites in *Aspergillus subramanianii* 1901NT-1.40.2 extract using UPLC-MS, isolate and elucidate the structure of individual compounds, and study the antimicrobial and cytotoxic activities of the isolated compounds. The structures of two previously unreported ergostane triterpenoid aspersubrin A (**1**) and pyrazine alkaloid ochramide E (**2**) were established using NMR and HR ESI-MS. The absolute configuration of **1** was determined using quantum chemical calculations. Moreover, the known polyketides sclerolide (**3**) and sclerin (**4**); the indolediterpene alkaloid 10,23-dihydro-24,25-dehydroaflavinine (**5**); the bis-indolyl benzenoid alkaloids kumbicin D (**6**), asterriquinol D dimethyl ether (**7**), petromurin C (**8**); and the cyclopentenedione asterredione (**9**) were isolated. The effects of compounds **3**-**9** on the growth and biofilm formation of the yeast-like fungus *Candida albicans* and the bacteria *Staphylococcus aureus* and *Escherichia coli* were investigated. Compounds **5** and **6** inhibited *C. albicans* growth and biofilm formation at an IC_50_ of 7–10 µM. Moreover, the effects of compounds **3**-**9** on non-cancerous H9c2 cardiomyocytes, HaCaT keratinocytes, MCF-10A breast epithelial cells, and breast cancer MCF-7 and MDA-MB-231 cells were also investigated. Compound **8** (10 µM) significantly decreased the viability of MCF-7 cells, inhibited colony formation, and arrested cell cycle progression and proliferation in monolayer culture. Moreover, **8** significantly decreased the area of MCF-7 3D spheroids by approximately 30%. A competitive test with 4-hydroxytamoxyfen and molecular docking showed that estrogen receptors (ERβ more than ERα) were involved in the anticancer effect of petromurin C (**8**).

## 1. Introduction

Marine fungi are known sources of various drugs and lead molecules, including antibiotics and anticancer agents. Cephalosporin C from the Mediterranean Sea isolated from the fungus *Cephalosporium acremonium* was the second most common β-lactam antibiotic [1]. Halimide was isolated from the marine green alga *Halimeda copiosa*-derived *Aspergillus* sp. CNC-139 [2] and then it was modified to obtain plinabulin with anti-microtubule anticancer action [3,4]. Between 2012 and 2023, 223 antibacterial compounds from marine fungi have been reported [5], of which only 50 have been identified as anti-candidal [6]. From 2019 to 2023, more than 20 compounds from marine fungi have been reported as promising in vitro anticancer agents, and 4,4′-bond secalonic acid D from *Penicillium oxalicum* and FGFC1 from *Stachybotrys longispora* FG216 have been successfully investigated in vivo against hepatocellular carcinoma and lung cancer [7].

An intensive study of marine fungi from the Vietnamese coastline has resulted in the isolation and investigation of several low-molecular-weight compounds with promising antimicrobial and anticancer activities. Tripeptide derivatives with a cinnamic acid moiety, asterripeptides A–C, from mangrove-derived *Aspergillus terreus* LM5.2, were found to be dual anti-staphylococcal and Nrf2-dependent anti-inflammatory compounds that can promote the healing of skin wounds infected with *Staphylococcus aureus* [8]. Anthraquinone vismione E from the marine sponge-derived fungus *Aspergillus* sp. 1901NT-1.2.2 showed anti-migratory and antiproliferative activities against breast cancer cells [9]. Mycophenolic acid derivatives with antiproliferative activity have been isolated from *Penicillium* sp. 1901NT-2.53.1 [10].

In a continuation of the study of biologically active fungal metabolites from Vietnamese sources, a number of fungal strains isolated from marine sponges of Nha Trang Bay was screened for their antimicrobial and cytotoxic activities [11]. The ethyl acetate extract of the culture of fungal strain 1901NT-1.40.2, isolated from the sponge *Cliona* sp., inhibited the viability of non-cancerous HaCaT and H9c2 cells by 50.3% and 40.1%, and cancerous HeLa and MCF-7 cells by 79.1% and 87.5%, respectively. This difference in toxicity between non-cancerous and cancer cells prompted us to focus on this strain, which was identified as *Aspergillus subramanianii* from the Circumdati section. The fungi in this section produce steroids, bis-indole benzoquinone, and pyrazine alkaloids, and several types of sclerotia contain anti-insect compounds [12].

Thus, the aims of this study were to investigate the metabolites in *the A. subramaniani* 1901NT-1.40.2 extract using a UPLC-MS approach, isolate and elucidate the structures of individual compounds, and study the antimicrobial and cytotoxic activities of the isolated compounds.

## 2. Results

### 2.1. UPLC-MS Analysis

**Figure 1:**
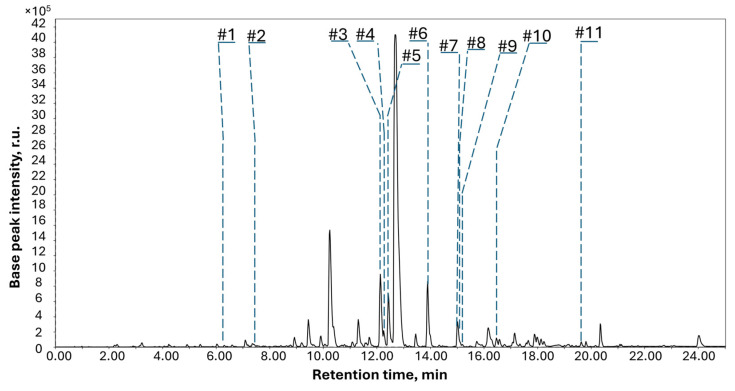
UPLC-MS profile (total ion current chromatogram) of the EtOAc extract of *Aspergillus subramanianii* 1901NT-1.40.2 culture.

The purified ethyl acetate extract of *A. subramaniani* 1901NT-1.40.2 cultivated on rice media was investigated using UPLC-MS, and the UPLC-MS data were analyzed using the Global Natural Product Social Molecular Networking (GNPS) database. Eleven peaks were annotated on the UPLC-MS chromatogram of *A. subramaniani* (Figure 1). A detailed list of the annotated compounds in the extract is provided in Table A1 (Appendix A).

For peaks #1–3, #5, #8, and #10, only molecular formulas corresponding to the exact masses were assumed. Peak #4 was detected at 12.50 min (*m*/*z* 318.2790, corresponding to C_21_H_35_NO [M + H]^+^) and showed MS/MS spectra similar to those of 2-benzyl-1-methyl-5-nonylpyrrolidin-3-ol [13] based on a comparison with the GNPS spectral library (MQScore 0.82). Peak #6 was detected at 13.90 min (*m*/*z* 429.1824, corresponding to C_24_H_36_O_5_ [M + H]^+^) and was annotated as the well known fungal bis-indolyl benzenoid alkaloid asterriquinol D dimethyl ether [7]. Peak #7 was detected at 15.05 min (*m*/*z* 427.2452, corresponding to C_24_H_36_O_5_ [M + Na]^+^) and annotated as the well known fungal polyketide lovastatin [14] based on the exact mass value and the same fragmentation pattern as that in the GNPS database.

Peak #9 was detected at 15.49 min at *m*/*z* 1202.85 ([M + H]^+^) and corresponded to the molecular formula of C_62_H_111_N_11_O_12_. It was assigned to the known fungal peptide cyclosporine A [15] based on the exact mass value and a comparison of the fragmentation pattern with those in the GNPS database (MQScore 0.83). Peak #11 was detected at 19.83 min (*m*/*z* 411.3265, corresponding to C_28_H_44_O_3_ [M – H_2_O + H]^+^) and was identified as ergosterol peroxide based on a comparison of the MS/MS spectrum and RT value with those of an in-house database [16].

**Figure 2:**
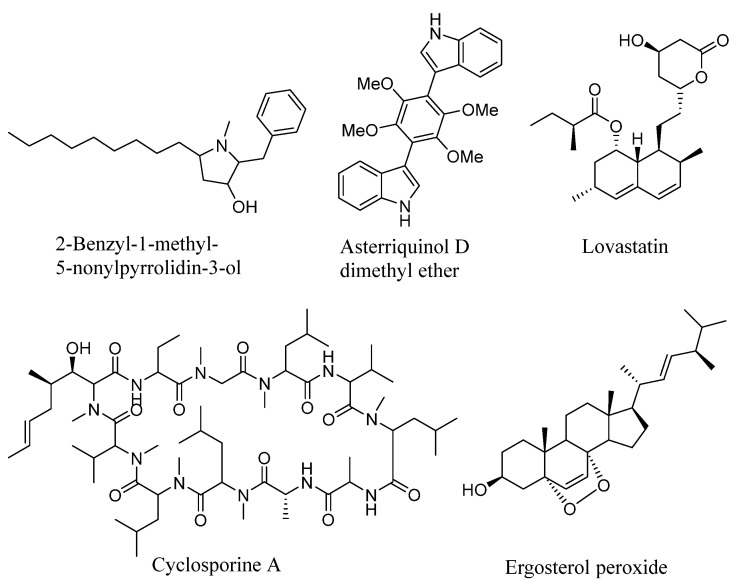
Compounds annotated in the EtOAc extract of *A. subramanianii* 1901NT-1.40.2 culture.

The structures of the annotated compounds are shown in Figure 2.

### 2.2. Isolation and Identification of Compounds ***1***–***9***

**Figure 3:**
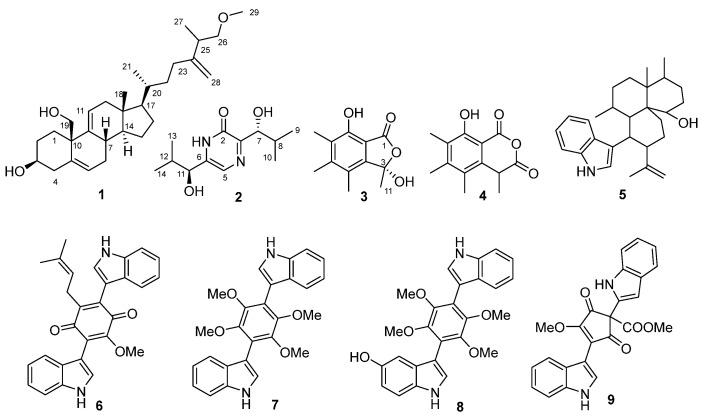
Compounds isolated from the EtOAc extract of *Aspergillus subramanianii* 1901NT-1.40.2 fungal strain.

The chromatographic separation of the EtOAc extract of *A. subramanianii* 1901NT-1.40.2 resulted in the isolation of nine individual compounds (Figure 3): new triterpenoid aspersubrin A (**1**), new pyrazine alkaloid ochramide E (**2**), known polyketides sclerolide (**3**) [17] and sclerin (**4**) [17], known indoloterpene alkaloid 10,23-dihydro-24,25-dehydroaflavinine (**5**) [18], known bis-indole alkaloids kumbicin D (**6**) [19], asterriquinol D dimethyl ether (**7**) [19], and petromurin C (**8**) [19], and asterredione (**9**) [20].

The molecular formula of compound **1** was determined to be C_29_H_46_O_3_ based on the HRESIMS data (*m/z* 465.3349 [M + Na]^+^), which corresponded to the ^13^C NMR data. An analysis of the ^1^H and ^13^C NMR data (Table 1, Figures S2–S7) of **1** showed the presence of three methyl groups (δ_C_ 11.9, δ_H_ 0.53; δ_C_ 17.4, δ_H_ 1.06; δ_C_ 18.6, δ_H_ 0.95), a methoxy group (δ_C_ 58.9, δ_H_ 3.34), nine *sp*^3^-methylenes (δ_C_ 23.4, δ_H_ 1.38, δ_H_ 1.80; δ_C_ 28.3, δ_H_ 1.14, δ_H_ 2.33; δ_C_ 28.5, δ_H_ 1.36, δ_H_ 2.00; δ_C_ 28.9, δ_H_ 1.91, δ_H_ 2.00; δ_C_ 31.4, δ_H_ 1.89, δ_H_ 2.13; δ_C_ 31.5, δ_H_ 1.51, δ_H_ 1.89; δ_C_ 37.9, δ_H_ 1.38, δ_H_ 1.82; δ_C_ 42.6, δ_H_ 2.16, δ_H_ 2.38), two oxygenated *sp*^3^-methylenes (δ_C_ 59.4, δ_H_ 3.40, δ_H_ 3.74; δ_C_ 77.1, δ_H_ 3.22, δ_H_ 3.39), a *sp*^2^-metylene (δ_C_ 108.5, δ_H_ 4.77), four *sp*^3^-methines (δ_C_ 38.8, δ_H_ 1.74; δ_C_ 40.0, δ_H_ 2.38; δ_C_ 51.8, δ_H_ 2.20; δ_C_ 56.4, δ_H_ 1.32), an oxygenated *sp*^3^-methines (δ_C_ 70.9, δ_H_ 3.67), two *sp*^2^-methines (δ_C_ 122.2, δ_H_ 5.46; δ_C_ 121.8, δ_H_ 5.51), two quaternary *sp*^3^-carbons (δ_C_ 42.1; δ_C_ 42.2), as well as three quaternary *sp*^2^-carbons (δ_C_ 136.1; δ_C_ 139.1; δ_C_ 152.8).

**Figure 4:**
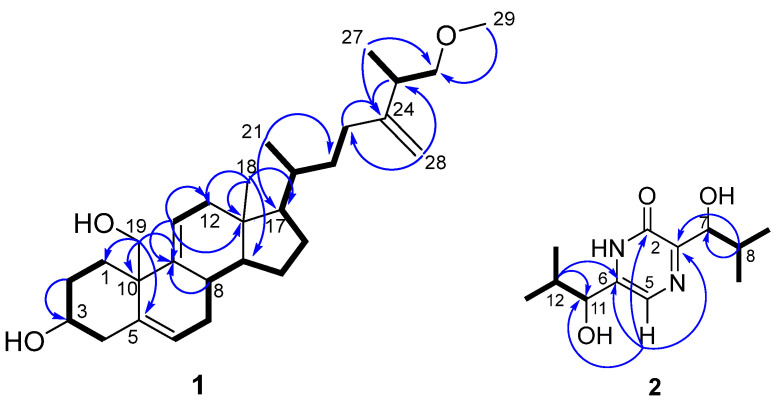
Key HMBC (arrows) and COSY (bold lines) correlations in compounds **1** and **2**.

The COSY correlations (Figure 4 and Figure S6) revealed the spin systems H(1)–H(2)–H(3)–H(4), H(6)–H(7)–H(8)–H(14)–H(15)–H(16)–H(17)–H(20)–(H(21))–H(22)–H(23), and H(26)–H(25)–H(27). HMBC correlations (Figure 4 and Figure S4) from H-1 (δ_H_ 2.33, 1.14) to C-4, C-10, and С-19, as well as from H-19α (δ_Н_ 3.74) to C-1, C-8, C-9, and C-10 were observed. The locations of methyl groups at C-18, С-21, and С-27, and a methoxy group at С-29 were determined by the HMBC correlations from Н-18 (δ_H_ 0.53) to C-12, C-13, and C-17, from Н-21 (δ_H_ 0.95) to C-17, С-20, and C-22, from Н-27 (δ_H_ 1.06) to С-24, C-25, and C-26, from Н-29 (δ_H_ 3.34) to С-26, as well as from Н-28 (δ_H_ 4.77) to С-23, C-24, and C-25.

Thus, the planar structure of compound **1** was established, and it was named aspersubrin A. Unfortunately, the insufficient amount and high lability of the isolated compound did not allow us to obtain NOE and ECD spectra to prove the stereoconfigurations of compound **1**. The stereochemistry of **1** was suggested based on its obvious biogenetic relationship with other ergostane triterpenoids [21]. 

The closest analogs of **1** are litosterol [22] and ergosta-5,7,9(11),24(28)-tetraenol [7]. Several ergostane derivatives with the simultaneous presence of double bonds Δ^5^ and Δ^9(11)^ [7] have been described, although they are conjugated with a double bond Δ^7^. Thus, aspersubrin A (**1**) is the first example of a Δ^5,9(11),24(28)^ ergostatriene derivative. In addition, the simultaneous oxidation of methyl groups C-19 and C-26 has not been previously described.

The molecular formula of compound **2** was determined to be C_12_H_20_N_2_O_3_ based on the HRESIMS data (*m/z* 263.1372 [M + Na]^+^) and was confirmed by ^13^C NMR data. An analysis of ^1^H and ^13^C NMR data (Table 1, Figures S8–S13) of **2** using DEPT and HSQC techniques revealed the presence of four methyl (δ_C_ 15.8, δ_H_ 0.76; δ_C_ 17.3, δ_H_ 0.99; δ_C_ 18.8, δ_H_ 0.95; δ_C_ 20.1, δ_H_ 1.08) and four methine (δ_C_ 31.9, δ_H_ 2.29; δ_C_ 34.6, δ_H_ 2.00;δ_C_ 73.8, δ_H_ 4.39; δ_C_ 75.1, δ_H_ 4.70) groups as well as a four *sp*^2^-carbons (δ_С_ 120.9, 139.3, 155.5, 158.1).

The COSY correlations (Figure 4 and Figure S11) revealed the spin systems H(9)–H(8)–(H(7))–H(10) and H(14)–H(12)–(H(11))–H(13). The HMBC correlations (Figure 4 and Figure S11) from H-5 (δ_H_ 7.25) to C-2, C-3, and С-6 and characteristic chemical shifts in the ^13^C NMR spectrum showed a structure of a pyrazinone ring. The HMBC correlations from H-5 (δ_H_ 7.25) to C-11, from H-12 (δ_H_ 2.00) to C-6, C-11, as well as from H-8 (δ_Н_ 2.29) to C-3 and C-7 showed the position of a methylpropyl side chain in **2**. Thus, compound **2** was elucidated as 3,6-bis(1-hydroxy-2-methylpropyl)pyrazin-2(1H)-one.

To establish the absolute stereostructure of **2**, the modified Mosher’s method was used. Unfortunately, attempts to obtain MTPA ethers resulted in the destruction of these compounds. Therefore, the stereostructure of **2** was established by calculating its ECD spectra using the TDDFT_B3LYP/6-311G(d)_PCM//B3LYP/6-311G(d)_PCM method. Theoretical ECD spectra were obtained via the statistical averaging of the ECD spectra calculated for twenty-four individual conformations of the stereoisomers of **2**. The conformations that input to the spectral properties that dominated are presented in Figure S44.

**Figure 5:**
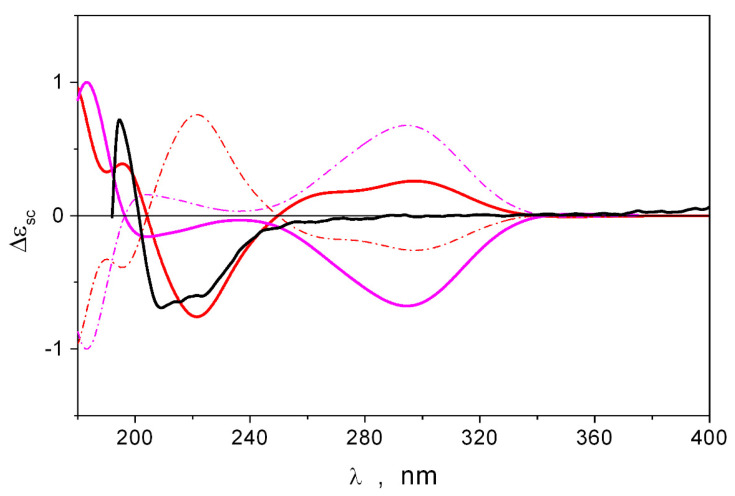
A comparison of the experimental (black line) and theoretical ECD spectra calculated for the 7*S,*11*S* (red), 7*S,*11*R* (magenta), 7*R,*11*R* (red dashdot), and 7*R,*11*S* (magenta dashdot) stereoisomers of **1**.

The calculated ECD spectra of the 7*S,*11*S*, 7*R,*11*R*, 7R,11*S*, and 7*S,*11*R* stereoisomers were compared with the experimental ECD spectrum of compound **2** (Figure 5). The theoretical spectrum of the 7*S,*11*S* stereoisomer describes well the shape of the experimental spectrum in the λ ≤ 260 nm region. Moreover, the mutual correspondence obtained for the 7*S,*11*S* stereoisomer was better than that for the 7*S,*11*R* stereoisomer and significantly better than that for the 7*R,*11*S* and 7*R,*11*R* stereoisomers. In the λ ≥ 260 nm region, the theory predicts the presence of several bands for all stereoisomers, but the best correspondence with the experimental spectrum was observed for the 7*S,*11*S* stereoisomer. Thus, the absolute stereostructure of compound **2** may be determined as 7*S,*11*S*.

The structure of compound **2** is very similar to that of ochramide A from *Aspergillus ochraceus* LCJ11-102 [23] and differs only in the additional hydroxyl groups at C-7 and C-11. Compound **2** was named ochramide E. 

**Figure 6:**
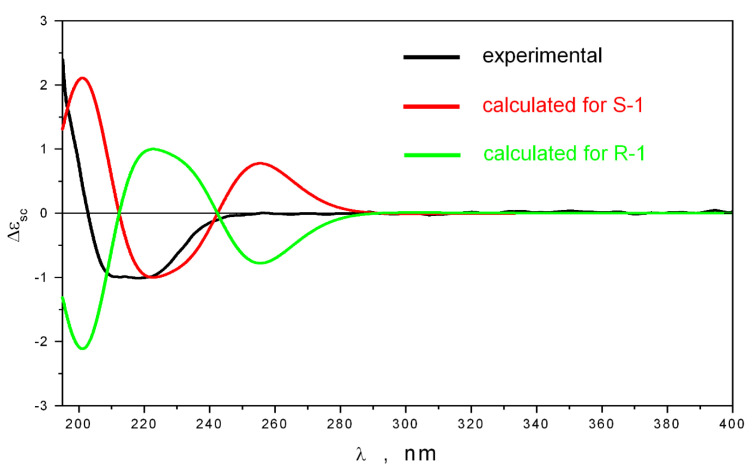
The experimental (black) and calculated ECD spectra for 3*S* (red) and 3*R* (green) stereoisomers of **3**.

Compound **3** had NMR and HRESIMS data that were the same as those for the well known fungal benzofuran sclerolide [17]. However, the configuration of the stereocenter at C-3 has not been described previously. The absolute stereochemistry of **3** was established using quantum chemical calculations of the ECD spectrum. The conformational analysis of **3** was performed using the TDDFT_B3LYP/6-311G(d)_PCM method. This shows that there were only two stable conformations of **3** regarding the rotation of the hydroxyl group around the C(3)–C(4) bond (Figure S31). The experimental and calculated ECD spectra of 3*S* and 3*R* stereoisomers of **3** are compared in Figure 6.

The experimental ECD spectrum contains the positive band at λ~200 nm and a wide negative band in the 205 < λ < 250 nm region. The intensive bands for λ > 250 nm are absent. Contrary to this, the theoretical spectra contain intensive bands in the λ > 250 nm region. Additional calculations were performed to determine the influence of large-amplitude motions on the shape of the theoretically calculated ECD curve. The internal rotations of the 3-O–H and C(8)-H_3_ groups and the rotation of the 7-O–H group (around the C(3)–OH, C(3)–C(8), and C(7)–OH bonds, respectively) were considered (Figure S46). We found that the rotation of the 3-O–H hydroxyl group in the direction of the **1_3** conformation (Figure S45) noticeably changed the intensities of the bands. In contrast, the shape of the ECD spectrum rests were nearly unchanged regarding the rotation of the methyl group. The rotation of the 7-O–H group noticeably changes the intensities of all bands, especially the bands in the λ < 240 nm region. In addition, the broadening of the negative band in the ECD spectrum of the 3*S* stereoisomer of **3** takes place in the region 210 < λ < 250 nm (Figure S47).

In all cases the bands in the λ > 250 nm region in the theoretical spectra maintain their high intensity. We performed additional single-point TDDFT calculations using an extended cc-pvTz basis set, owing to investigating the basis set dependence of the ECD spectrum in the λ > 250 nm region (Figure S48). We found that, in all cases, the theory predicted high rotational powers for transitions in this region. This may mean that a used standard calculation scheme fails to describe the features of the ECD spectrum of **3** in the λ > 250 nm region. 

At the same time, the theory qualitatively correctly describes the shape of experimental UV and ECD spectra in the region 195 < λ < 255 nm (for the 3*S* stereoisomer). In contrast, the ECD spectrum calculated for the 3*R* stereoisomer disagrees with the experimental ECD spectrum for all frequency diapasons. This allowed us to conclude that the absolute configuration of the stereocenter of **3** is 3*S*. 

In addition to the new compounds **1** and **2** and the known sclerolide (**3**), six other known compounds were isolated. Compounds **4**–**9** were identified by comparing their NMR, MS, and ECD data (Figures S14–S44) with those reported for polyketide sclerin (**4**) [17]; indoloterpene alkaloid 10,23-dihydro-24,25-dehydroaflavinine (**5**) [18]; bis-indolyl benzenoid alkaloids kumbicin D (**6**) [19], asterriquinol D dimethyl ether (**7**) [19], and petromurin C (**8**) [19]; and cyclopentenedione asterredione (**9**) [20].

The isolated compounds corresponded to the peaks identified in the HPLC-MS chromatogram (Figure 1, Table A1). Sclerolide (**3**), sclerin (**4**), 10,23-dihydro-24,25-dehydroaflavinine (**5**), petromurin C (**8**), kumbicin D (**6**), and asterredione (**9**) were detected in the HPLC chromatogram as peaks #1, #2, #10, #5, #8, and #3, respectively. Due to insufficient amounts, aspersubrin A (**1**) and ochramide E (**2**) could not be used as reference compounds and were not detected in the HPLC-MS chromatogram.

New compounds **1** and **2** were isolated in very small amounts, which were insufficient for investigating their biological activity. The antimicrobial and cytotoxic activities of compounds **3**–**9** were investigated.

### 2.3. Antimicrobial and Antibiofilm Activities of Isolated Compounds

The effects of compounds **3**–**9** on the growth and biofilm formation of the yeast-like fungus *Candida albicans* and bacteria *Staphylococcus aureus* and *Escherichia coli* were studied. The MIC_50_s are shown in Table 2.

Sclerolide (**3**) half-maximally inhibited the growth of *S. aureus* and *E. coli* at 20.34 and 21.07 µM, respectively. *S. aureus* and *E. coli* biofilm formations were prevented by 50% at 31.14 and 43.51 µM, respectively. The antibacterial activity of sclerolide has not been previously reported, and our data are the first.

Sclerin (**4**) only prevented *C. albicans* biofilm formation by 20.22 ± 5.24% at 100 µM but did not affect its growth and *S. aureus*. and *E. coli* growth and biofilm formation up to 100 µM.

10,23-Dihydro-24,25-dehydroaflavinine (**5**) exhibited high activity against all test strains. The total inhibition (MIC_100_s) of *S. aureus*, *E. coli*, and *C. albicans* growth was observed at 6.85 ± 0.53 µM, 12.45 ± 1.02 µM, and 11.48 ± 0.90 µM, respectively, and the MIC_100_s of *S. aureus*, *E. coli*, and *C. albicans* biofilm formation were 10.45 ± 0.96 µM, 23.08 ± 2.00 µM, and 12.01 ± 1.01 µM, respectively. The half-maximal inhibitory concentrations are listed in Table 2. Compound 5 has been reported to exhibit anti-insect activity [24] and is active against *E. coli* with an MIC_50_ value of 2.0 μg/mL [25]. Thus, the data on the significant effect of 10,23-dihydro-24,25-dehydroaflavinine (**5**) on the growth and biofilm formation of *S. aureus*, *E. coli*, and *C. albicans* strains are novel.

Kumbicin D (**6**) also had significant effects on the growth and biofilm formation of all the tested strains, with an MIC_50_s of 7-11 µM. Earlier, kumbicin D (**6**) was isolated from a culture of the marine sponge-associated fungus *Aspergillus candidus* KUFA 0062 and tested against *S. aureus* ATCC 29213, *E. faecalis* ATCC 29212, methicillin-resistant *S. aureus* (MRSA), and vancomycin-resistant enterococci strains, but it was not active [26]. This may be explained by the peculiarities of the strains used in these studies.

Asterriquinol D dimethyl ether (**7**) at 100 µM inhibited *S. aureus* growth by 42.24 ± 2.07% but did not affect their biofilm formation, as well as *E. coli* and *C. albicans* growth and biofilm formation.

Petromurin C (**8**) half-maximally inhibited the growth of all test strains at 26–52 µM. However, it half-maximally prevented the biofilm formation of *S. aureus* at more than 100 µM and *E. coli* at 83.01 µM, and had a more specific effect on *C. albicans* biofilm formation with an IC50 of 24.12 µM. Earlier studies showed that petromurin C (**8**) did not inhibit the growth of *S. aureus* ATCC 29213 and *E. faecalis* ATCC 29212 but exhibited a synergistic effect with oxacillin against MRSA *S. aureus* 66/1 [26]. Thus, this is the first report of the inhibitory effect of **8** on *C. albicans* biofilm formation.

Asterredione (**9**) at 100 µM only prevented *C. albicans* biofilm formation by 41.18 ± 4.91%, and did not affect bacterial *S. aureus* and *E. coli* growth and biofilm formation.

### 2.4. Cytotoxic Activity of Isolated Compounds ***3***–***9***

**Figure 7:**
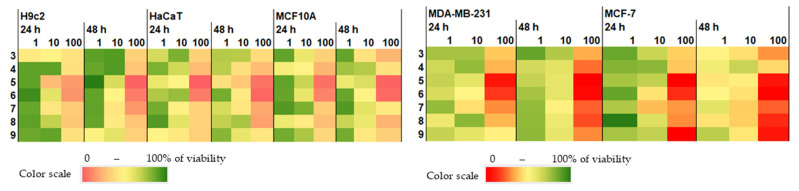
Effects of compounds **3**–**9** on the viability of normal and cancer cells.

The effects of compounds **3**–**9** on the viability of non-cancerous H9c2 cardiomyocytes, HaCaT keratinocytes, MCF-10A breast epithelium cells, and MCF-7 and MDA-MB-231 breast cancer cells were studied, and the results are presented as a heatmap (Figure 7) and in the Table A2.

Sclerolide (**3**) exhibited weak toxicity at concentrations up to 100 µM, decreasing the viability of non-cancerous H9c2 and HaCaT cells by 30.0–37.5% and cancerous MDA-MB-231 and MCF-7 cells by 39.1–46.9% at 100 µM, respectively. Non-cancerous MCF10A cells were more sensitive to compound **3**, which decreased their viability by half at approximately 100 µM. Previously, the high cytotoxicity and genotoxicity of the AcOEt extract of *Sclerotinia sclerotiorum* fungus, which mainly contains scleroderolide, sclerotiorin, sclerominol, sclerolide (**3**), and sclerosporin, have been reported [27], but no cytotoxicity of individual **3** has not been reported. 

The half-maximal concentrations were calculated for compounds **4**–**9** and are listed in Table 3.

Sclerin (**4**) and asterriquinol D dimethyl ether (**7**) were more toxic for non-cancerous cells than for cancerous cells. 10,23-Dihydro-24,25-dehydroaflavinine (**5**) and kumbicin D (**6**) exhibited moderate toxicity to both non-cancerous and cancerous cells. Previously, only weak effects of **6** at 100 µM on HepG2, HT29, HCT116, A549, A375, MCF-7, U251, and T98G cells were reported [26]. The toxic effect of kumbicin D (**6**) on non-cancerous cells was significantly less than its inhibitory influence on *C. albicans* biofilm formation, making kumbicin D (**6**) a very interesting anti-Candida agent.

**Figure 8:**
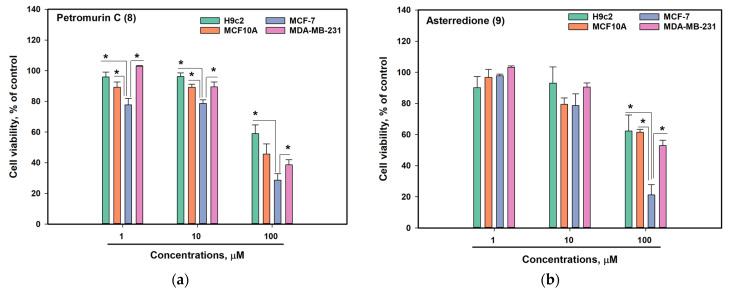
The viability of non-cancerous H9c2 and MCF10A cells and breast cancer MCF-7 and MDA-MB-231 cells after 48 h of treatment with petromurin C (**8**) (**a**) and asterredione (**9**) (**b**). * indicates significance of differences (*p* < 0.05) between variants.

Because the selective effect of petromurin C (**8**) and asterredione (**9**) on MCF-7 breast cancer cells was observed, a detailed analysis of the cytotoxic activities of **8** and **9** was performed (Figure 8). 

Petromurin C (**8**) exhibited moderate effects on cell viability, with a more pronounced impact on the breast cancer MCF-7 cells than on the non-cancerous breast MCF10A cells at all studied concentrations (Figure 8a). Indeed, petromurin C (**8**) was more active against MCF-7 cells even at a concentration of 1 µM. In [26], petromurin C (**8**) also induced a significant decrease in cell viability, with IC_50_ values ranging from 34.8 µM in H29 cells to 94.8 µM in MCF-7 cells, but was not effective against human malignant glioma U251 and T98G cells. Moreover, the anti-leukemic effect of petromurin C (**8**) has been previously described [28]. However, the specific activity of petromurin C (**8**) against breast cancer cells has not been reported.

Asterredione (**9**) at 100 µM for 48 h decreased the viability of non-cancerous H9c2 cells by 38%, HaCaT cells by 23%, and was not toxic for these cell lines at 10 µM. Although the IC_50_s of **9** were calculated to be approximately 50 µM for MCF-7 cancer cells and more than 100 µM for MCF-10A non-cancer cells, **9** at 10 µM did not show selectivity against MCF-7 cells (Figure 8b). Moreover, **9** at 1 µM did not exhibit cytotoxic effects on cancerous cells. In an earlier study, compound **9** has been reported to exhibit moderate cytotoxic activity against non-small cell lung cancer NCIH460, breast cancer MCF-7, and CNS glioma SF-268 cells [20].

Thus, petromurin C (**8**) has demonstrated more promising anticancer properties, and its activity has been studied in detail.

### 2.5. Petromurin C (***8***) Activity Against MCF-7 Breast Cancer

**Figure 9:**
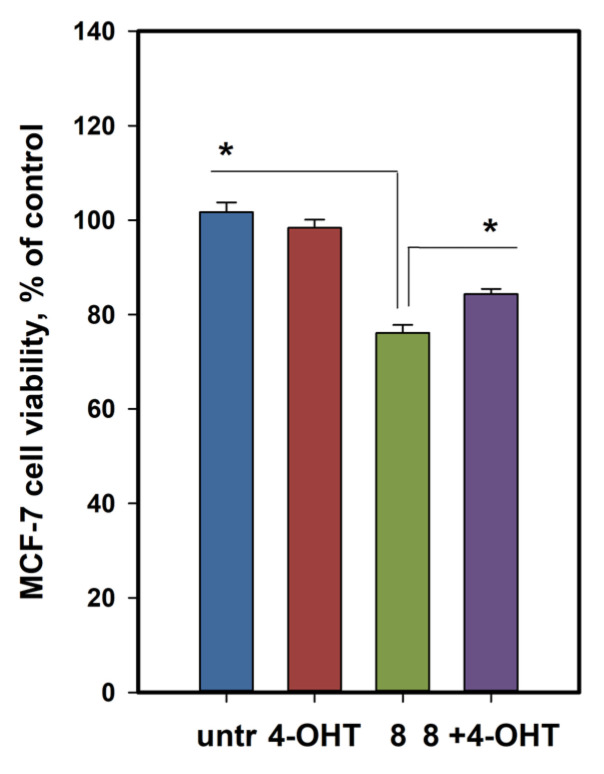
Influence of petromurin C (**8**) on the viability of 4-hydroxytamoxyfen-pretreated MCF-7 cells. * indicates significance of differences (*p* < 0.05) between variants.

Since estrogen-dependent breast cancer MCF-7 cells were proposed to be more sensitive to compound **8**, the estrogen receptor (ER) dependence of petromurin C (**8**) cytotoxicity to estrogen-dependent MCF-7 cells was investigated using 4-hydroxytamoxifen (4-OHT) as an ERs antagonist. MCF-7 cells were pretreated with 4-OHT at a nontoxic concentration of 0.1 µM for 1 h, and **8** at 10 µM was added for 48 h. The measured cell viabilities are shown in Figure 9.

The viability of MCF-7 cells treated with **8** was 76.1 ± 1.6%, whereas that of 4-OHT-pretreated cells treated with **8** was 84.4 ± 1.1%. One-way ANOVA confirmed the significance of the differences between the two groups (*p* = 0.015). Therefore, pretreatment with 4-OHT decreased the effect of **8**, indicating at least the partial involvement of ERs in the anticancer activity of petromurin C (**8**). 

**Figure 10:**
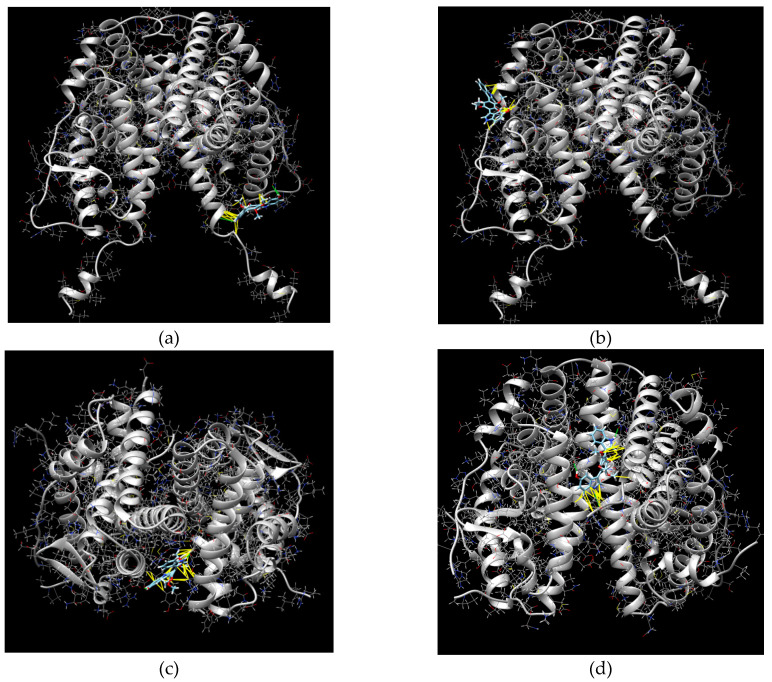
Most calculated complexes of **8** with ERα (PDB ID 1A52) (**a**,**b**) and ERβ (PDB ID 5TOA) (**c**,**d**).

Molecular docking of **8** with ERα and ERβ structures (PDB IDs 1A52 and 5TOA, respectively) was performed. The calculated data are presented in Table 4 and shown in Figure 10.

The LBD of ERα is folded into a three-layered antiparallel α-helical sandwich comprising a central core layer of three helices (H5/6, H9, and H10) sandwiched between two additional layers of helices (H1–4 and H7, H8, H11), and the E_2_ binding cavity is formed by parts of H3 (Met 342 to Leu 354), H6 (Trp 383 to Arg 394), H8 and the preceding loop (Val 418 to Leu428), H11 (Met 517 to Met 528), H12 (Leu 539 to His 547), and the S1/S2 hairpin (Leu 402 to Leu 410) [29]. The two ERs have similar LBDs; however, in the hydrophobic pocket of the LBDs, Leu384 and Met421 of ERα are substituted with Met336 and Ile373, respectively, in ERβ [30].

Therefore, the calculated complex of **8** with ERα was located outside the ligand-binding site of ERα and had only two hydrogenic and three hydrophobic interactions. The calculated complexes of **8** with ERβ had lower ∆G values and were located in the gap between the two monomers. These complexes had two or three hydrogenic bindings and six hydrophobic interactions. Thus, petromurin C (**8**) may affect ERβ dimerization.

**Figure 11:**
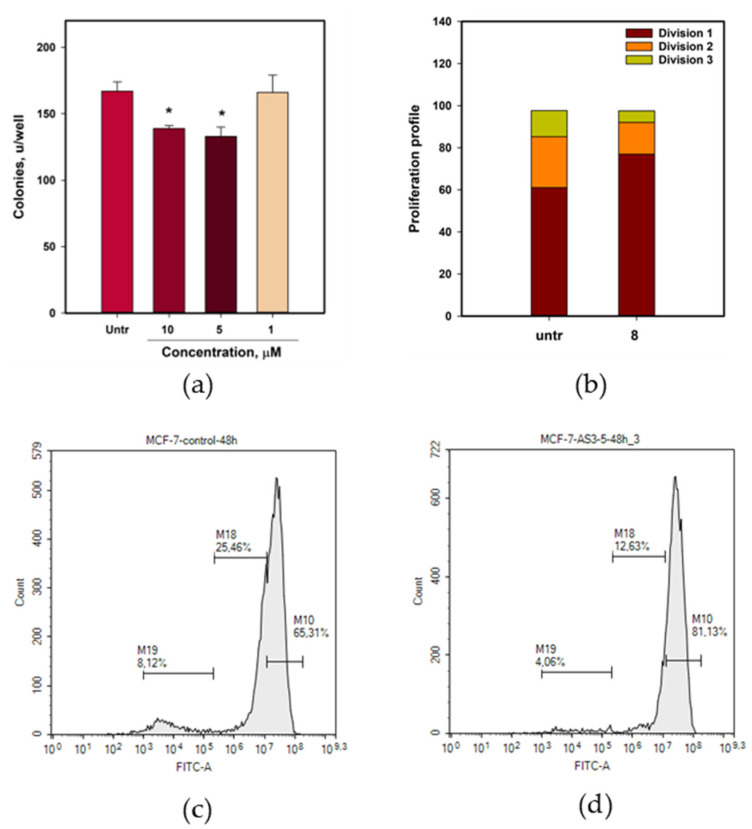
Effect of petromurin C (**8**) on MCF-7 colony formation (**a**). All data are presented as mean ± SEM from three independent experiments. Effect of petromurin C (**8**) on MCF-7 cell proliferation profile in the CFDA SE assay (**b**–**d**). Percentage of cells in each division (**a**) and representative images of untreated (**b**) and treated (**c**) cells in flow cytometry profile. The compound was used at a concentration of 5 µM. * indicates significance of differences (*p* < 0.05) between variants.

The effects of compound **8** on MCF-7 cell colony formation were also investigated (Figure 11a). Compound **8** at 5 and 10 µM inhibited MCF-7 colony formation by 16.8 and 20.4%, respectively.

The effect of compound **8** on MCF-7 cell proliferation was evaluated using CFDA SE staining and flow cytometry (Figure 11b–d). Untreated MCF-7 cells were divided into three groups based on their CFDA fluorescence intensities after 48 h (Figure 11c). The percentages of cells in divisions 1, 2, and 3 were 61.4%, 24.3%, and 12.3%, respectively. Petromurin C (**8**), at a concentration of 5 µM, significantly inhibited MCF-7 cell proliferation (Figure 11d). The percentages of cells in divisions 1–3 were 76.9%, 15.2%, and 5.5%, respectively.

The effect of petromurin C (**8**) on MCF-7 cell cycle progression was studied using flow cytometry (Table 5).

In untreated cells, the percentages of cells in the S phases were stable (47.4–47.9%), while the percentage of G1 phase cells increased from 24.3% to 31.7%, and the percentage of G2/M phase cells decreased from 27.8% to 20.8%. In cells treated with **8** at 5 µM, the differences in cell cycle phase were insignificant after 1 h of treatment, and an increase in the percentage of S phase cells (56.6%) and a decrease in G1 and G2/M phase cells (26.2% and 17.2%, respectively) were detected. In cells treated with **8** at 10 µM, changes in cell cycle progression were detected earlier. The percentage of S-phase cells was 53.1% and 51.8% after 1 and 3 h, respectively. Accordingly, the percentage of G2/M phase cells decreased to 25.9% and 14.9% after 1 and 3h of treatment, respectively. Thus, petromurin C (**8**) may block the transition from the synthetic phase to the G2 phase during cell cycle progression. 

**Figure 12:**
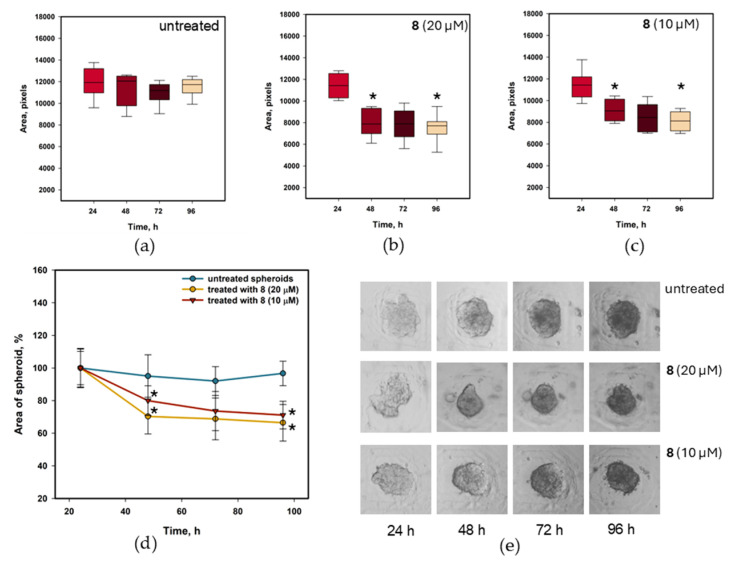
Effect of petromurin C (**8**) on the area of MCF-7 spheroids. The graphs illustrate the changes in area during the observation of untreated spheroids (**a**) and spheroids treated with **8** at 10 µM (**b**) and 20 µM (**c**), and the graph combines all data (**d**). At least 20 of the same spheroids in each variant were analyzed daily, and the data were calculated as the mean ± SEM. Representative images of spheroids during observation (**e**). * indicates significance of differences (*p* < 0.05) between variants.

The anticancer effect of petromurin C (**8**) was investigated using a 3D culture of MCF-7 cells (Figure 12). MCF-7 spheroids were formed in agarose microwells using MSLA-printed stamps [31]. Compound **8** (10 µM and 20 µM) was added 24 h after the cells were seeded. The results of the spheroid area measurements are presented in Figure 12.

The area of untreated spheroids decreased less after 48 h but remained stable thereafter. The images confirmed that this was caused by cell compaction. After 96 h, the spheroids were less compact, which caused degradation.

Treatment with petromurin C (**8**) significantly decreased the area of MCF-7 spheroids. Treatment with 10 µM petromurin C (**8**) decreased the spheroid area by 19.1% (*p* = 0.0422) and 28.9% (*p* = 0.000252) after 24 and 72 h, respectively. Treatment with 20 µM petromurin C (**8**) decreased the spheroid area by 29.8% (*p* = 0.00483) and 32.6% (*p* = 0.000261) after 24 and 72 h, respectively. 

## 3. Discussion

Thus, the *A. subramanianii* 1901NT-1.40.2 strain derived from the Vietnamese marine sponge *Cliona* sp. was found as a source of secondary metabolites with antimicrobial and anticancer activities. 

10,23-Dihydro-24,25-dehydroaflavinine (**5**) is a promising new antibiotic that inhibited the growth and biofilm formation of all test strains, with MIC_50_s less than 10 µM. Moreover, 10,23-dihydro-24,25-dehydroaflavinine (**5**) and kumbicin D (**6**) significantly prevented the formation of *C. albicans* biofilms, and this activity is noteworthy. 

Biofilm formation by the yeast-like fungus *C. albicans* is a significant problem in medical practice because biofilms are more resistant to antibiotics than planktonic forms. The mechanism of biofilm formation includes the adhesion of individual cells to the surface, followed by cell proliferation and filamentation, when cells form elongated protrusions that continue to grow, forming filamentous hyphae (initiation stage); this is followed by the accumulation of an extracellular polysaccharide matrix as the biofilm matures (maturation stage); and in the last stage, the cells are released from the biofilm into the environment, where they can populate other surfaces (scattering stage) [32].

The effect of indole-derived molecules on *C. albicans* biofilm formation may be due to the mimicking of the *C. albicans* autoantibiotic tryptophol (3-β-hydroxyethylindole) structure [33]. Tryptophol, farnesol ((2E,6E)-3,7,11-trimethyldodeca-2,6,10-triene-1-ol), farnesoic acid, and tyrosol (2-[4-hydroxyphenyl]ethanol) are signaling molecules involved in *C. albicans* biofilm formation [34,35]. Tryptophol and farnesol inhibited hyphal formation, whereas tyrosol stimulated the proliferation and formation of hyphae. Some compounds inhibit the formation of *C. albicans* biofilms, probably due to their structural similarity to tryptophol [36]. For example, the synthetic indole derivative MMV688768 blocks hyphal elongation [37]. All indole derivatives from *A. subramanianii* inhibited *C. albicans* biofilm formation in the following order: **6** ≥ **5** > **8** > **9** > **7**. Probably, a vicinal location of indole and prenyl (in **6**) or isopropenyl (in **5**) substituent is a key factor for the significant effects of 10,23-dihydro-24,25-dehydroaflavinine (**5**) and kumbicin D (**6**) on the biofilm formation of *C. albicans*. 

**Figure 13:**
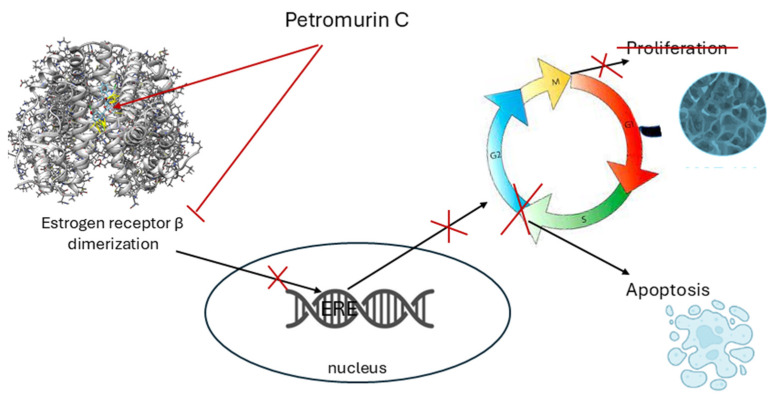
Proposed scheme of petromurin C antiproliferative action.

Some isolated compounds were toxic to both non-cancerous and cancerous cells. Asterredione (**9**) showed selectivity against MCF-7 breast cancer cells at concentrations greater than 10 µM, and petromurin C (**8**) exhibited selective action against MCF-7 cells at concentrations of 10 µM and below. Petromurin C (**8**) significantly decreased the viability of MCF-7 cells and inhibited their proliferation and transition from the S phase to the G2/M phase of the cell cycle in monolayer culture. ERs (ERβ more than ERα) are involved in the anticancer effects of petromurin C. Moreover, the anticancer potential of this fungal metabolite was confirmed in 3D cultured MCF-7 cells. Recently, petromurin C (**8**) was reported to decrease the viability of myeloid leukemia U937 cells and the size (but not amount) of U937 colonies, as well as induce apoptotic cell death via the activation of the intrinsic cell death pathway, concomitant with mitochondrial stress [28]. In addition, petromurin C synergized with the clinically used FLT3 inhibitor gilteritinib at sub-toxic concentrations. We propose that the observed effects are due to the action of petromurin C (**8**) on ERs, as shown in Figure 13. 

ERs controlled cell cycle progression in ER-dependent cells, and the antagonistic action of **8** on ERs due to the prevention of dimerization resulted in cell cycle arrest and proliferation blocking, followed by apoptotic cell death. Interestingly, asterriquinol D dimethyl ether (**7**), which does not have an additional hydroxyl group in the indole moiety compared with **8**, was less active against breast cancer cells and more toxic to H9c2 cells than petromurin C (**8**). Molecular docking calculations showed that this hydroxyl group formed hydrogenic binding with ERα and ERb. Thus, the hydroxyl group in the petromurin C structure is necessary for the interactions of **8** with its molecular targets and for its anticancer activity. 

Earlier, it was reported that some fungal metabolites are ER-targeted antiproliferative agents. The polyketide gispolone, isolated from *Phellinus lonicerinus,* had a pronounced effect on the activity of ERa and ERb and the associated antiproliferative effect against breast cancer cells MCF-7 [38]. Subglutinol A from *Fusarium subglutinans* exerts an antiestrogenic effect by competing with 17b-estradiol for binding to ERa [39]. Petromurin C (**8**) is the first bisindole–benzoquinone compound with ER-dependent anti-breast cancer activity.

According to the GLOBOCAN 2020 database, there were 2.3 million cases of breast cancer in women and approximately 685,000 deaths in 185 countries in 2020, with 70% of all new cases and 81% of all deaths occurring in able-bodied and socially active women aged 50 and older [40]. It is assumed that by 2040, the number of newly diagnosed breast cancer cases will grow by more than 40% and amount to approximately 3 million cases per year; therefore, mortality from breast cancer may increase by more than 50%, from 685,000 in 2020 to 1 million in 2040 [40]. Therefore, the discovery of new anti-breast cancer agents is crucial for drug development, and a detailed investigation of the mechanism of action of petromurin C (**8**) is warranted. 

## 4. Materials and Methods

### 4.1. General Experimental Procedures

Optical rotations were measured on a Perkin Elmer 343 polarimeter (Perkin Elmer, Waltham, MA, USA). UV spectra were recorded using a Shimadzu UV-1601PC spectrometer (Shimadzu Corporation, Kyoto, Japan) in methanol. CD spectra were measured using a Chirascan-Plus CD spectrometer (Leatherhead, UK) in methanol. NMR spectra were recorded on Bruker DPX-300, Bruker DPX-500, Bruker DPX-600, and Bruker DRX-700 spectrometers (Bruker BioSpin GmbH, Rheinstetten, Germany), using TMS as an internal standard. Low-pressure liquid column chromatography was performed using an ODS gel (12 nm, S-75 µM, YMC CO., Kyoto, Japan). HRESIMS spectra were obtained using a Maxis Impact mass spectrometer (Bruker Daltonics GmbH, Rheinstetten, Germany).

Low-pressure liquid column chromatography was performed using silica gel (50/100 μm; Imid Ltd., Krasnodar, Russia). Plates pre-coated with silica gel (5–17 μm, 10 cm × 10 cm, Imid Ltd., Krasnodar, Russia) and 60 RP-18 F254S silica gel (20 cm × 20 cm, Merck KGaA, Darmstadt, Germany) were used for thin-layer chromatography. Preparative HPLC was performed on an HPLC system consisting of a PrimeLine Binary pump (Analytical Scientific Instruments, Inc., El Sobrante, CA, USA) with an RI-101 refractometer (Shoko Scientific Co. Ltd., Yokohama, Japan), and a Shimadzu LC-20 chromatograph (Shimadzu USA Manufacturing, Canby, OR, USA) with a Shimadzu RID-20A refractometer (Shimadzu Corporation, Kyoto, Japan) using semi-preparative columns YMC ODS-AM (YMC Co., Ishikawa, Japan) (5 µm, 10 mm × 250 mm), HyperClone ODS(C-18) (Phenomenex, 5 µm, 4.6 mm × 250 mm, Torrance, CA, USA), and YMC SIL (YMC Co., Ishikawa, Japan) (5 µm, 10 mm × 250 mm) columns.

### 4.2. Fungal Strain

The fungal strain 1901NT-1.40.2 was isolated from a sponge *Cliona* sp. collected at a depth ranging from 8 to 10 m by scuba diving in Nha Trang Bay, Vietnam (12°10′ N, 109°16′ E). The fungus was identified as *Aspergillus subramanianii* using gene sequence analysis of the ITS region and named *Aspergillus subramanianii* 1901NT-1.40.2 (GenBank accession number MN577309) [11]. The strain was preserved at the Marine Microorganism Collection of the Institute of Oceanography, Vietnam Academy of Science and Technology (IO, VAST).

### 4.3. Cultivation of Strain 1901NT-1.40.2

The fungus was cultured in 80 × 500 mL Erlenmeyer flasks, each containing rice (20.0 g), yeast extract (20.0 mg), KH_2_PO_4_ (10 mg), and natural seawater from Nha Trang Bay (40 mL) at 28 °C for three weeks.

### 4.4. UPLC-MS Analysis of Fungal Extracts

HPLC-MS analysis was performed using a Bruker Elute UHPLC chromatograph (Bruker Daltonics, Bremen, Germany) connected to a Bruker Impact II Q-TOF mass spectrometer (Bruker Daltonics, Bremen, Germany). An InfinityLab Poroshell 120 SB-C18 column (2.1 × 150 mm, 2.7 μm; Agilent Technologies, Santa Clara, CA, USA) was used for chromatographic separation. Chromatographic separation and mass spectrometric detection were performed as described previously [41].

### 4.5. Extraction and Isolation

The fungal mycelia and medium were extracted with EtOAc (24.0 L) and evaporated in vacuo to yield a crude extract (27.7 g). The dry residue was dissolved in H_2_O–EtOH (4:1) (200 mL) and extracted successively with n-hexane (3 × 0.25 L), EtOAc (5 × 0.2 L), and butanol-1 (3 × 0.15 L). The ethyl acetate extract was evaporated to dryness (17.3 g) and chromatographed on a silica gel column (5 × 65 cm), which was first eluted with n-hexane–EtOAc from 100% n-hexane with a stepwise gradient of 0% to 100% EtOAc (total volume 60 L). Fractions of 250 mL were collected and combined based on TLC (toluene−isopropanol, 6:1 and 3:1, v/v). Seven fractions were obtained: AS-1-1 (16 mg), AS-1-2 (1.3 g), AS-1-3 (100 mg), AS-1-4 (1.3 g), AS-1-5 (1.2 g), AS-1-6 (1.1 g), and AS-1-7 (4.3 g).

The fraction eluted with 5% EtOAc was purified using column chromatography on a Sephadex LH-20 (column 1 × 65 cm) in a CHCl_3_ solvent system. Eight subfractions were obtained. The first and fifth subfractions were purified by HPLC on a Nautilus 110-5-C18 R column eluting with MeCN to obtain sclerin (**4**) (4 mg) and 10,23-dihydro-24,25-dehydroaflavinine (**5**) (15 mg). 

The fraction eluted with 10% EtOAc was purified by column chromatography on a Sephadex LH-20 (column 1 × 65 cm) in a CHCl_3_ solvent system. Ten subfractions were obtained. The fourth subfraction was purified on an ODS-A column eluted with MeOH–H_2_O (60:40) to yield subfraction AS-5-2 (18 mg), which was purified on a YMC-ODS-AM column eluted with MeCN–H_2_O (40:60) to yield sclerolide (**3**) (1.5 mg). 

The fraction eluted with 20% EtOAc was purified by column chromatography on a Sephadex LH-20 (column 2 × 90 cm) in a CHCl_3_ solvent system. As a result, 12 subfractions were obtained. Asterriquinol D dimethyl ether (**7**) (8 mg) was obtained from the sixth subfraction. Kumbicins D (**6**) (300 mg) was obtained from the twelfth subfraction. The subfraction (AS-10-4) was chromatographed on a silica gel column (1 × 15 cm), which was first eluted with n-hexane–EtOAc from 100% n-hexane using a stepwise gradient from 0% to 100% EtOAc. The subfraction eluted with 75% EtOAc was purified by HPLC on a Heper Clove MeCN–H_2_O (90:10) to yield **1** (1.1 mg). The subfraction (AS-10-9) was chromatographed on a silica gel column (1 × 15 cm), which was first eluted with n-hexane–EtOAc from 100% n-hexane with a stepwise gradient of 0% to 100% EtOAc. The subfractions eluted with 15% and 25% EtOAc were purified by HPLC on a Heper Clove MeOH–H_2_O (50:50) to yield ochramide E (**2**) (0.6 mg) and asterredione (**9**) (0.7 mg). The subfraction (AS-10-9) was chromatographed on a silica gel column (1 × 15 cm), which was first eluted with n-hexane–EtOAc from 100% n-hexane with a stepwise gradient of 0% to 100% EtOAc. The subfraction eluted with 25% EtOAc was purified by HPLC on a Phenomenex Fusion-RP MeOH–H_2_O (60:40) to yield petromurin C (**8**) (3.5 mg). 

### 4.6. Spectral Data of Isolated Compounds

Aspersubrin A (**1**):; ^1^H and ^13^C NMR data, see Figures S2–S7; HRESIMS m/z 359.1102 [M + Na]^+^ (calcd. for C_17_H_20_O_7_Na 359.1101, ∆ −0.3 ppm) (Figure S36, Supplementary Materials).

Ochramide E (**2**): [α]^20^_D_ –145.5 (с 0,01, MeOH);UV (MeOH) λmax(log_ε_) 290 (2.49), 243 (3.30), 195 (3.53), 393 (1.61), 275 (2.47), 217 (3.13); CD (c 0.23 mM, MeOH), λmax (Δε) 195 (7.69), 244 (3.08), 252 (2.28) nm; ^1^H and ^13^C NMR data, see Figures S8–S13; HRESIMS m/z 263.1372 [M + Na]^+^ (calcd. for C_29_H_46_O_3_Na 263.1366, ∆ −2.1 ppm) (Figure S37, Supplementary Materials).

### 4.7. Quantum Chemical Modeling

Theoretical calculations were performed using the B3LYP exchange–correlation functional, the polarization continuum model (PCM), and the 6-311G(d) and cc-pvTz basis sets implemented in the Gaussian 16 package of programs [42]. 

The statistical weights (g*_im_*) of the individual conformations were calculated according to the following equation:

(1) gim=e−ΔGim/RT∑ie−ΔGim/RT

where Δ*G_im_* = *G_i_* – *G_m_* is the relative Gibbs free energies and index “*m*” denotes the most stable conformation. 

The time-dependent density functional theory (TDDFT) was used for calculating UV and ECD spectra. The Gauss-type functions with the bandwidths ζ = 0.34 eV were used for simulating the individual bands in theoretical spectra.

The scaled theoretical and experimental ECD spectra were obtained according to the following equation:

(2) Δεsc(λ)=Δε(λ)Δε(λpeak)

where the denominator |Δε(λ_peak)|_ is a modulo of the peak value for the positive characteristic bands.

### 4.8. Bioassays

#### 4.8.1. Bacterial Strains and Antimicrobial Assays

The Gram-positive bacterial *Staphylococcus aureus* ATCC 21027, Gram-negative bacterial *Escherichia coli* VKPM (B-7935), and yeast-like fungal *Candida albicans* KMM 455 strains were fermented on solid medium Mueller Hinton broth with agar (16.0 g/L) in a Petri dish at 37 °C for 24 h. 

The antimicrobial activity of the compound was tested at concentrations ranging from 100 µM and lower. The effect of the compound on bacterial growth was estimated as described by [43]. Gentamicin and amphotericin B were used as positive controls against *S. aureus*, *E. coli,* and *C. albicans*, and a 1% dimethyl sulfoxide (DMSO) solution in PBS was used as a negative control. The optical density of the bacterial suspension after 18 h was measured at λ = 620 nm. The effect of the compound on biofilm formation for 18 h was tested using MTT reagent (Sigma-Aldrich, St. Louis, MO, USA) in accordance with [44]. The optical density of the obtained solution was measured at λ = 570 nm. A Multiskan FS spectrophotometer (Thermo Scientific Inc., Beverly, MA, USA) was used for both assays. The results were calculated as percentages of the control data and presented as minimal concentrations reduced by 50% of growth or biofilm formation (MIC_50_).

#### 4.8.2. Cell Cultures and Cultivation

Rat cardiomyocyte H9c2 cells were kindly provided by Prof. Dr. Gunhild von Amsberg from the Martini-Klinik Prostate Cancer Center, University Hospital Hamburg-Eppendorf, Hamburg, Germany. The human HaCaT keratinocyte cell line was kindly provided by Prof. N. Fusenig (Cancer Research Centre, Heidelberg, Germany). Non-cancerous human breast epithelial MCF-10A cells were kindly provided by Dr. Irina Zhitnyak (N. N. Blokhin National Medical Research Center of Oncology, Moscow, Russia). Human breast cancer MCF-7 HTB-22 and MDA-MB-231 cells were obtained from the ATTC (Manassas, VA, USA). 

H9c2 and HaCaT cells were cultured in DMEM with 10% fetal bovine serum and 1% penicillin/streptomycin (BioloT, St. Petersburg, Russia). MCF-7 and MDA-MB-231 cells were cultured in MEM (BioloT, St. Petersburg, Russia) with 10% fetal bovine serum (FBS) and 1% penicillin/streptomycin (BioloT, St. Petersburg, Russia). MCF 10A cells were cultured in DMEM F12 medium (BioloT, St. Petersburg, Russia) supplemented with 20% FBS, 20 ng/mL hrEGF (Sci-Store, Moscow, Russia), and 1% penicillin/streptomycin (BioloT, St. Petersburg, Russia). All cells were cultured at 37 °C in a humidified atmosphere containing 5% CO_2_.

H9c2 cells were seeded at a concentration of 3 × 10^3^ cells/well. MCF-10A, MCF-7, and MDA-MB-231 cells were seeded at 5 × 10^3^ cells/well, and HaCaT cells were seeded at 1 × 10^4^ cells/well. The experiments were initiated after 24 h.

#### 4.8.3. Cell Viability Assay

Compounds at concentrations up to 100 µM were added to the wells for 24h or 48 h, and the viability of the cells was measured using an MTT (3-(4,5-dimethylthiazol-2-yl)-2,5-diphenyltetrazolium bromide) assay, which was performed according to the manufacturer’s instructions (Sigma-Aldrich, Munich, Germany). All compounds were dissolved in DMSO, such that the final concentration of DMSO in the cell culture did not exceed 1%. DMSO was used as a control. The results are presented as percentages of the control data.

#### 4.8.4. Competitive Assay with 4-Hydroxytamoxifen

The antagonist of estrogenic receptors, 4-hydroxytamoxifen (Lumiprobe, Moscow, Russia), was dissolved in DMSO at a concentration of 10 mM and stored with protection from light. The probe was then added to the cells at a final concentration of 1 µM. The investigated compounds were added to the cells after 1 h. Then the viability of MCF-7 cells was measured after 48 h using MTT assay (Section 4.8.3).

#### 4.8.5. Molecular Docking of Petromurin C (**8**) with Estrogen Receptors

The PDB files of estrogenic receptors α (PDB ID 1A52) and β (PDB ID 5TOA) were obtained from the RCSB Protein Data Bank (https://www.rcsb.org, accessed on 20 November 2024) and prepared for docking using the PrepDock package of UCFS Chimera 1.16. The chemical structures of the compounds were prepared for docking using ChemOffice and verified using the PrepDock package of UCFS Chimera 1.16 software. Docking was conducted on the SwissDock online server (http://old.swissdock.ch/docking, Date of the access 20 November 2024) using the EADock DSS docking software [45]. The algorithm involves the generation of many binding modes in the vicinity of all target cavities (blind docking), the estimation of their CHARMM energies via the Chemistry at Harvard Macromolecular Mechanics (CHARMM) algorithm [46] to evaluate the binding modes with the most favorable energies from the Fast Analytical Continuum Treatment of Solvation (FACTS) [47], and finally, the clustering of these binding modes [48].

The predicted building models were visualized and analyzed using the UCFS Chimera 1.16 software. Docking parameters, such as Gibb’s free energy (ΔG, kcal/mol), full fitness score (FF, kcal/mol), hydrogen bonding (H-bond), and hydrophobic interactions, were used to analyze the target/ligand complexes, as described in [49].

#### 4.8.6. The Breast Cancer MCF-7 Cell Colony Formation Assay

The effect of the compound on colony formation by MCF-7 cells was assessed using a clonogenic assay [50]. The concentration of MCF-7 cells was 0.33 × 10^3^/mL. The cells were incubated for 10 d, fixed with methanol (25 min), stained with 0.5% crystal violet solution (25 min), and washed with PBS. The grown colonies were counted using a BIO-PRINT-Cx4 Edge-Fixed Pad-Container (Vilber, Collegien, France). The results are presented as colony inhibition compared to that of the control.

#### 4.8.7. Proliferation Assay

MCF-7 cells at a concentration of 1.2 × 10^4^ cells/well were seeded in a 12-well plate for 24 h. After adhesion, the cells were stained with (5,6)-carboxyfluorescein succinimidyl ester (CFDA SE) dye (LumiTrace CFDA SE kit; Lumiprobe, Moscow, Russia). Thereafter, the compound (5 µM) was added to the wells.

After 48 h of incubation, the cells were washed twice with PBS, scraped, and collected. The intensity of CFDA fluorescence was analyzed using a NovoCyte flow cytometer (Agilent, Austin, TX, USA), and the data were analyzed as previously described [9].

#### 4.8.8. Cell Cycle Investigation

After incubation, the cells were trypsinized, harvested, washed with PBS, and fixed with ice-cold 70% ethanol in a dropwise manner prior to storage at −20 °C overnight. The cells were then washed with PBS, incubated with 200 μg/mL RNAse (PanReac, AppliChem, Darmstadt, Germany) and 20 μg/mL of propidium iodide (Sigma-Aldrich, St. Louis, MO, USA) for 30 min at 37 °C, and DNA content was analyzed using a NovoCyte flow cytometer (Agilent, Austin, TX, USA). The proportion of cells in each cell cycle phase was expressed as a percentage.

#### 4.8.9. Three-Dimensional Culture of MCF-7 Cells and Treatment with Petromurin C (**8**)

MCF-7 cell spheroids were formed in agarose microwells as described previously [31]. Briefly, agarose (2.8%) heated to 60 °C was added to each well of a 96-well plate, and MSLA-printed stamps with microcylinders were inserted and then removed after agarose solidification. Each well contained 34 microwells with a diameter of 300 μm. MCF-7 cells at a concentration of 2 × 10^4^ cells/well were added to each well and gently centrifuged to precipitate the cells that had gathered in the microwells. Petromurin C (**8**) was added after 24 h, and observations were performed daily using an MIB-2-FL fluorescence microscope (Lomo Microsystems, St. Petersburg, Russia) The spheroid area was calculated using CellProfiler software v. 4.2.8 [51,52]. In total, at least 20 of the same spheroids in each variant were analyzed daily and data were calculated as mean ± SEM. 

#### 4.8.10. Statistical Data Evaluation

All bioassays were conducted with three biological replicates and with three technical replicates. Data are expressed as mean ± standard error of the mean (SEM). Student’s t-test was performed using SigmaPlot 14.0 (Systat Software Inc., San Jose, CA, USA) to determine the statistical significance. Differences were considered statistically significant at *p* < 0.05.

## 5. Conclusions

Thus, the *A. subramanianii* 1901NT-1.40.2 strain is a source of new triterpenoid aspersubrin A, new pyrazine alkaloid ochramide E, known polyketides sclerolide and sclerin, indoloterpene alkaloid 10,23-dihydro-24,25-dehydroaflavinine, and a number of known bis-indole alkaloids. 10,23-Dihydro-24,25-dehydroaflavinine (**5**) is interesting as an antimicrobial agent. Petromurin C (**8**) significantly decreased the viability of MCF-7 cells and inhibited their proliferation in monolayer cultures, and its anticancer potential was confirmed in 3D cultured MCF-7 cells. ERs (ERβ more than ERα) are involved in the anticancer effect of petromurin C.

## Figures and Tables

**Table 1:** NMR data (500 MHz, CDCl_3_, *δ* in ppm) for compounds **1** and **2**.

Position	2		1	
	δ_С_, mult	δ_H_ (*J* in Hz)	δ_С_, mult	δ_H_ (*J* in Hz)
1			28.3, CH_2_	**a:** 2.33 dt, (3.5, 13.6) **b:**1.14, m
2	158.1, C		37.9, CH_2_	**a:** 1.82, m **b:**1.38, m
3	155.5, C		70.9, CH	3.67, m
4			31.5, CH_2_	**a:** 1.51, m **b:** 1.89, m
5	120.9, CH	7.25, s	139.1, C	
6	139.3, C		122.2, CH	5.46 d, (6.1)
7	73.8, CH	4.39, d (6.0)	29.8, CH_2_	**a:** 2.00, m **b:** 1.91, m
8	31.9, CH	2.29, m	38.8, CH	1.74, m
9	20.1, CH_3_	1.08, d (6.8)	136.1, C	
10	15.8, CH_3_	0.76, d (6.7)	41.2, C	
11	75.1, CH	4.70, (3.7)	121.8, CH	5.51 d, (6.1)
12	34.6, CH	2.00, m	42.6, CH_2_	**a:** 2.38, m **b:** 2.16, m
13	18.8, CH_3_	0.95, d (6.8)	42.1, C	
14	17.3, CH_3_	0.99, d (6.7)	51.8, CH	2.20, m
15			23.4, CH_2_	**a:** 1.80, m **b:** 1.38, m
16			28.5, CH_2_	**a:** 2.00, m **b:** 1.36, m
17			56.4, CH	1.32, m
18			11.9, CH_3_	0.53, s
19			59.4, CH_2_	**a:** 3.40, m **b:** 3.74, d (10.8)
20			36.1, CH	1.45, m
21			18.6, CH_3_	0.95, d (6.5)
22			34.6, CH_2_	**a:** 1.62 m **b:** 1.18 m
23			31.4, CH_2_	**a:** 2.13 m **b:** 1.89 m
24			152.8, C	
25			40.0, CH	2.38, m
26			77.1 CH_2_	**a:** 3.22, m **b:** 3.39, m
27			17.4, CH_3_	1.06, d (6.7)
28			108.5, CH_2_	4.77, m
29			58.9, CH_3_	3.34, s

**Table 2:** Antimicrobial and antibiofilm activities of **3**–**9**.

Compound	*S. aureus*	*E. coli*	*C. albicans*
	Growth inhibition, MIC_50_, µM		
**3**	20.34 ± 2.58	21.07 ± 2.11	>100
**4**	>100	>100	>100
**5**	3.12 ± 0.30	4.92 ± 0.45	7.01 ± 0.54
**6**	9.38 ± 0.77	11.42 ± 1.02	10.44 ± 1.07
**7**	>100	>100	>100
**8**	26.74 ± 1.98	52.14 ± 2.01	34.14 ± 2.1
**9**	>100	>100	>100
Gentamycin	4.52 ± 0.98	5.03 ± 0.77	-
Amphotericin B	-	-	1.18 ± 0.15
	Biofilm formation, MIC_50_, µM		
**3**	31.14 ± 2.34	43.51 ± 4.03	>100
**4**	>100	>100	>100
**5**	3.08 ± 0.30	11.04 ± 0.95	10.01 ± 1.00
**6**	7.98 ± 0.57	10.34 ± 0.98	8.36 ± 0.95
**7**	>100	>100	>100
**8**	>100	83.01 ± 6.42	24.12 ± 3.07
**9**	>100	>100	>100
Gentamycin	4.34 ± 0.32	5.34 ± 0.50	-
Amphotericin B	-	-	1.38 ± 0.14

**Table 3:** Cytotoxicity (IC_50_, µM) of some isolated compounds.

Cell Line	Time, h	Compound					
		4	5	6	7	8	9
H9c2	24	>100	9.8 ± 1.3	72.2 ± 4.1	>100	>100	>100
	48	57.2 ± 2.4	57.2 ± 3.0	65.4 ± 2.1	69.9 ± 2.3	>100	>100
HaCaT	24	≥ 100	52.8 ± 1.1	>100	>100	>100	>100
	48	≥100	25.3 ± 0.8	49.4 ± 1.2	80.5 ± 1.8	80.5 ± 1.8	>100
MCF10A	24	93.4 ± 3.5	61.5 ± 1.3	65.7 ± 1.9	>100	≥100	>100
	48	>100	57.1 ± 2.8	55.6 ± 2.3	>100	91.0 ± 4.1	>100
MDA-MB-231	24	>100	62.9 ± 3.1	74.4 ± 0.6	>100	>100	>100
	48	94.1 ± 3.2	61.3 ± 2.1	58.2 ± 2.0	85.1 ± 1.5	79.9 ± 1.2	≥100
MCF-7	24	>100	58.6 ± 4.2	62.2 ± 2.2	>100	>100	51.3 ± 2.6
	48	>100	53.5 ± 1.1	47.3 ± 1.7	79.1 ± 2.4	61.5 ± 1.2	55.1 ± 3.9

**Table 4:** Molecular docking calculations of petromurin C complexes with estrogen receptors.

Complex	∆G, kcal/mol	H-Bond	Hydrophobic Interactions
ERα (PDB ID 1A52)	−7.21	Asn348, Lys529	Val533, Gly344, and Lys529
	−6.788	Leu320, Trp393	Ile326, Trp393, Leu320
ERβ (PDB ID 5TOA)	−8.54	Glu389, Met410	Ala427, Leu430, Leu426, Met410, Tyr411, Ala382
	−8.26	Glu389, Met410, Asn431	Ala427, Ser423, Leu430, Leu426, Tyr411, Ala382

**Table 5:** MCF-7 cell cycle phase disturbance after petromurin C (**8**) treatment.

Time, h	Treatment	Cell Disturbance, %		
		G1 Phase	S Phase	G2/M Phase
1	Untreated	24.31 ± 1.27	47.91 ± 2.06	27.80 ± 0.79
	**8** at 5 µM	22.69 ± 0.42	45.52 ± 1.05	29.91 ± 1.95
	**8** at 10 µM	21.37 ± 2.18	53.15 ± 1.53 ^#^	25.96 ± 0.62 ^#^
3	Untreated	31.72 ± 0.30	47.49 ± 1.44	20.80 ± 1.37
	**8** at 5 µM	26.20 ± 1.17 *	56.60 ± 1.25 *	17.20 ± 1.23 *
	**8** at 10 µM	33.82 ± 0.98	51.79 ± 0.86 *	14.95 ± 1.08 *

^#^ indicates significance of differences (*p* < 0.05) between untreated and treated cells after 1 h of treatment; * indicates significance of differences (*p* < 0.05) between untreated and treated cells after 3 h of treatment.

## Data Availability

The original contributions presented in this study are included in the article/supplementary materials. Further inquiries can be directed to the corresponding author/s.

## Supplementary Materials

The following supporting information can be downloaded at: https://www.mdpi.com/article/10.3390/md23090353/s1, Figures S1–S26: The NMR spectra of compounds **1**–**9**; Figures S27–S34: ECD spectra of compounds **2**–**9**; Figures S35–S43: HRESIMS spectra of compounds **1**–**9**; Figures S44–S48: The additional data on ECD calculations.

## Appendix A

**Table A1:** The list of annotated compounds in the *Aspergillus subramanianii* 1901NT-1.40.2 fungal strain extract.

Peak	RT, min	Measured *m/z*	MF	Calculated *m/z*	Error, ppm	Name, ^Identification Level^ and (Number of Isolated Compound)	MQScore	Ref	Structure
#1	6.42	223.0971	C_12_H_14_O_4_	223.097583 [M + H]^+^	−2.17	Sclerolide ^a^ (**3**)		[17]	
#2	7.70	235.0970	C_13_H_14_O_4_	235.096485 [M + H]^+^	2.19	Sclerin ^a^ (**4**)		[17]	
#3	12.18	415.1291	C_24_H_18_N_2_O_5_	415.129945 [M + H]^+^	−2.04	Asterredione ^a^ (**9**)		[20]	
#4	12.50	318.2779	C_21_H_35_NO	318.279141 [M + H]^+^	−3.89	2-Benzyl-1-methyl-5-nonylpyrrolidin-3-ol ^b^	0.82	[13]	
#5	12.87	445.1742	C_26_H_24_N_2_O_5_	445.175798 [M + H]^+^	−3.58	Petromurin C ^a^ (**8**)		[19]	
#6	13.90	429.1824	C_26_H_24_N_2_O_4_	429.181981 [M + H]^+^	0.98	Asterriquinol D dimethyl ether ^a,b^ (**7**)	0.73	[19]	
#7	15.05	427.2452	C_24_H_36_O_5_	427.246593 [M + Na]^+^	3.26	Lovastatin ^b^		[14]	
		405.2634		405.2634648 [M + H]^+^	0.16				
		422.2891		422.291197 [M + NH_4_]^+^	4.97				
#8	15.01	437.1864	C_28_H_24_N_2_O_3_	437.185969 [M + H]^+^	0.99	Kumbicin D ^a^ (**6**)		[19]	
#9	15.49	1202.8488	C_62_H_111_N_11_O_12_	1202.848645 [M + H]^+^	0.13	Cyclosporine ^b^	0.83	[15]	
#10	16.18	406.3091	C_28_H_39_NO	406.311538 [M + H]^+^	−3.30	10,23-Dihydro-24,25-dehydroaflavinine ^a^ (**5**)		[18]	
#11	19.83	411.3265	C_28_H_44_O_3_	411.3257 [M − H_2_O + H]^+^	−1.94	Ergosterol peroxide ^a^		[16]	

^a^—identification was made by comparing the RT, MS, and MS/MS data with those of authentic standards; ^b^—structure was annotated by comparing the obtained MS/MS data with available data in the GNPS database or in the literature.

## Appendix B

**Table A2:** The effect of compounds **3**–**9** on the cell viability (% of control).

Compound	Time, h	Concentrations, µM		
		1.0	10.0	100.0
	H9c2			
**3**	24	81.1 ± 3.2	88.7 ± 4.4	74.6 ± 8.3
	48	102.0 ± 5.5	102.7 ± 6.7	62.5 ± 4.6
**4**	24	101.9 ± 5.8	101.9 ± 6.1	73.5 ± 4.4
	48	101.4 ± 5.6	102.6 ± 7.4	56.2 ± 0.9
**5**	24	101.9 ± 2.9	50.5 ± 1.8	33.6 ± 0.3
	48	105.6 ± 3.4	94.0 ± 3.0	10.1 ± 0.2
**6**	24	104.0 ± 12.9	92.9 ± 6.1	30.9 ± 2.9
	48	99.7 ± 3.7	88.6 ± 2.3	21.6 ± 2.2
**7**	24	101.5 ± 1.3	76.9 ± 2.8	59.0 ± 5.3
	48	102.8 ± 7.6	76.3 ± 3.9	32.8 ± 1.4
**8**	24	100.9 ± 1.7	95.9 ± 2.3	57.4 ± 8.5
	48	101.6 ± 2.2	79.8 ± 4.6	55.8 ± 5.5
**9**	24	101.1 ± 6.6	101.7 ± 3.2	73.1 ± 5.1
	48	90.2 ± 7.1	93.0 ± 10.4	62.2 ± 10.4
	HaCaT			
**3**	24	94.4 ± 5.9	100.2 ± 3.0	63.0 ± 8.6
	48	96.8 ± 1.0	96.6 ± 4.1	69.4 ± 3.5
**4**	24	100.2 ± 6.6	94.7 ± 5.0	49.5 ± 6.0
	48	96.4 ± 1.4	72.1 ± 11.1	51.5 ± 2.1
**5**	24	92.3 ± 5.0	88.3 ± 8.6	7.8 ± 0.3
	48	72.2 ± 4.3	56.8 ± 4.8	16.8 ± 0.2
**6**	24	97.2 ± 5.7	98.6 ± 3.5	21.5 ± 3.3
	48	100.5 ± 3.3	80.0 ± 0.1	11.6 ± 3.5
**7**	24	100.7 ± 3.8	85.6 ± 7.6	64.1 ± 3.2
	48	94.2 ± 8.7	78.1 ± 6.4	40.1 ± 2.9
**8**	24	102.7 ± 7.7	93.1 ± 6.9	64.3 ± 1.9
	48	95.8 ± 1.9	97.7 ± 1.4	36.8 ± 7.0
**9**	24	88.7 ± 3.0	79.2 ± 2.1	62.0 ± 6.9
	48	99.5 ± 2.3	92.6 ± 11.2	77.1 ± 1.5
	MCF10A			
**3**	24	101.7 ± 1.3	92.2 ± 3.5	62.5 ± 2.2
	48	101.6 ± 5.1	74.4 ± 2.4	47.5 ± 7.0
**4**	24	90.8 ± 7.8	88.6 ± 5.2	47.0 ± 3.2
	48	98.0 ± 4.0	96.8 ± 2.1	73.5 ± 1.1
**5**	24	101.7 ± 4.4	95.4 ± 2.3	16.1 ± 8.6
	48	92.0 ± 0.9	93.9 ± 6.4	9.9 ± 0.2
**6**	24	101.6 ± 3.1	92.4 ± 3.6	23.9 ± 6.2
	48	98.4 ± 4.5	87.4 ± 2.7	13.7 ± 3.2
**7**	24	102.7 ± 5.1	100.2 ± 3.8	59.6 ±2.1
	48	97.7 ± 1.4	93.1 ± 3.6	78.9 ± 5.7
**8**	24	91.9 ± 9.5	81.4 ± 1.0	52.0 ± 4.0
	48	95.3 ± 3.4	92.1 ± 2.1	45.7 ± 6.6
**9**	24	100.7 ± 0.9	97.5 ± 3.5	66.3 ± 3.6
	48	96.7 ± 5.1	79.4 ± 4.3	61.5 ± 1.9
	MDA-MB-231			
**3**	24	99.8 ± 1.3	100.6 ± 5.7	68.6 ± 8.3
	48	107.6 ± 2.8	99.0 ± 5.1	60.9 ± 3.6
**4**	24	94.8 ± 4.2	102.7 ± 4.9	63.1 ±1.6
	48	94.3 ± 3.9	91.8 ± 7.5	47.1 ± 6.2
**5**	24	96.3 ± 7.5	91.3 ± 6.1	21.1 ± 0.9
	48	103.2 ± 2.4	102.3 ± 4.1	10.6 ± 0.2
**6**	24	93.6 ± 4.8	84.5 ± 6.7	32.7 ± 5.2
	48	100.6 ± 2.2	90.8 ± 1.2	14.7 ± 0.6
**7**	24	102.7 ± 1.6	75.9 ± 4.8	53.6 ± 4.2
	48	99.8 ± 3.7	82.9 ± 6.3	43.5 ± 2.5
**8**	24	89.4 ± 2.4	103.6 ± 2.2	63.7 ±4.8
	48	102.9 ± 0.5	89.5 ± 3.1	38.6 ± 1.7
**9**	24	96.3 ± 3.2	106.3 ± 9.9	82.5 ± 1.3
	48	103.2 ± 0.9	90.5 ± 2.7	52.9 ± 3.4
	MCF-7			
**3**	24	107.2 ± 8.9	95.6 ± 2.4	70.2 ±5.5
	48	83.8 ± 3.1	76.0 ± 2.6	55.1 ± 1.0
**4**	24	104.3 ± 4.6	103.4 ± 5.4	68.3 ± 4.5
	48	88.5 ± 4.7	95.9 ± 3.5	74.1 ± 4.5
**5**	24	100.5 ± 0.9	97.0 ± 2.6	10.0 ± 1.0
	48	78.5 ± 9.9	81.6 ± 3.3	21.2 ± 0.1
**6**	24	107.6 ± 6.5	77.7 ± 7.5	29.9 ± 0.8
	48	84.6 ± 9.4	76.8 ± 3.8	12.1 ± 2.7
**7**	24	99.4 ± 4.4	65.4 ± 2.9	56.0 ± 4.5
	48	91.5 ± 6.3	70.0 ± 5.2	43.9 ± 3.5
**8**	24	105.4 ± 5.9	92.8 ± 3.0	53.7 ± 6.8
	48	77.7 ± 3.7	70.6 ± 8.5	28.6 ± 4.3
**9**	24	100.5 ± 7.0	97.0 ± 11.2	10.0 ± 8.1
	48	97.8 ± 1.1	78.6 ± 7.6	21.2 ± 6.6
