# Supplementary material for: Secondary Metabolites of the Marine Sponge-Derived Fungus Aspergillus subramanianii 1901NT-1.40.2 and Their Antimicrobial and Anticancer Activities
Source: Mar Drugs. 2025 Aug 30;23(9):353. doi: 10.3390/md23090353 (PMC12471299; doi:10.3390/md23090353)

Supplementary Materials

Secondary metabolites of the marine sponge-derived fungus *Aspergillus subramanianii* 1901NT-1.40.2 and their antimicrobial and anticancer activities

Olga O. Khmel [1,2], Anton N. Yurchenko [1], Phan Thi Hoai Trinh [3], Ngo Thi Duy Ngoc [3], Vo Thi Dieu Trang [3], Huynh Hoang Nhu Khanh [3], Alexandr S. Antonov [1], Konstantin A. Drozdov [1], Roman S. Popov [1], Natalya Y. Kim[1], Dmitrii V. Berdyshev [1], Ekaterina A. Chingizova [1], Ekaterina S. Menchinskaya [1], and Ekaterina A. Yurchenko [1,*]

G.B. Elyakov Pacific Institute of Bioorganic Chemistry Far Eastern Branch of Russian Academy of Science, prospect 100-letiya Vladivostoka, 159, Vladivostok 690022, Russia; yurchenkoan@piboc.dvo.ru (A.N.Y.); antonov_as@piboc.dvo.ru (A.S.A.); drovsh@yandex.ru (K.A.D.); popov_rs@piboc.dvo.ru (R.S.P.); kim_ny@piboc.dvo.ru (N.Y.K.); berdyshev@piboc.dvo.ru (D.V.B.); chingizova_ea@piboc.dvo.ru (A.R.C.); ekaterinamenchinskaya@gmail.com (E.S.M);

1 Far Eastern Federal University, 10 Ajax Bay, Russky Island, Vladivostok 690922, Russia; khmel.oo@dvfu.ru (O.O.K.)

2 Institute of Oceanography, Vietnam Academy of Science and Technology, Nha Trang 650000, Vietnam; phanhoaitrinh84@gmail.com (P.T.H.T.); duyngoc@io.vast.vn (N.T.D.N); votrang@io.vast.vn (V.T.D.T); khanhhuynh@io.vast.vn (H.H.N.K)

* Correspondence: eyurch@piboc.dvo.ru (E.A.Y.)

Figure S1. ^{1}H NMR spectrum (500 MHz, $CDCl_3$) for Compound 1 5

Figure S2. ^{13}C NMR spectrum (125.5 MHz, $CDCl_3$) of Compound 1 9

Figure S3. DEPT-135 spectrum (500 MHz, $CDCl_3$) of Compound 1 10

Figure S4. HSQC spectrum (500 MHz, $CDCl_3$) of Compound 1 11

Figure S5. ^{1}H-^{1}H COSY spectrum (500 MHz, $CDCl_3$) of Compound 1 12

Figure S6. HMBC spectrum (500 MHz, $CDCl_3$) of Compound 1 13

Figure S7. ^{13}C NMR spectrum (125.5 MHz, $CDCl_3$) of Compound 2 14

Figure S8. DEPT-135 spectrum (500 MHz, $CDCl_3$) of Compound 2 15

Figure S9. ^{1}H NMR spectrum (500 MHz, $CDCl_3$) of Compound 2 16

Figure S10. ^{1}H-^{1}H COSY spectrum (500 MHz, $CDCl_3$) of Compound 2 17

Figure S11. HSQC spectrum (500 MHz, $CDCl_3$) of Compound 2 18

Figure S12. HMBC spectrum (500 MHz, $CDCl_3$) of Compound 2 19

Figure S13. ^{1}H NMR spectrum (500 MHz, $CDCl_3$) for Compound 3 20

Figure S14. ^{13}C NMR spectrum (125.5 MHz, $CDCl_3$) of Compound 3 20

Figure S15. ^{1}H NMR spectrum (500 MHz, $CDCl_3$) for Compound 4 21

Figure S16. ^{13}C NMR spectrum (125.5 MHz, $CDCl_3$) of Compound 4 21

Figure S17. ^{1}H NMR spectrum (500 MHz, CD_3OD) for Compound 5 22

Figure S18. ^{13}C NMR spectrum (125.5 MHz, CD_3OD) of Compound 5 22

Figure S19. ^{1}H NMR spectrum (500 MHz, DMSO) for Compound 6 23

Figure S20. ^{13}C NMR spectrum (125.5 MHz, DMSO) of Compound 6 23

Figure S21. ^{1}H NMR spectrum (500 MHz, DMSO) for Compound 7 24

Figure S22. ^{13}C NMR spectrum (125.5 MHz, DMSO) of Compound 7 24

Figure S23. ^{1}H NMR spectrum (500 MHz, $acenote_{d6}$) for Compound 8 25

Figure S24. ^{13}C NMR spectrum (125.5MHz, $acenote_{d6}$) of Compound 8 25

Figure S25. ^{1}H NMR spectrum (500 MHz, $CDCl_3$) for Compound 9 26

Figure S26. ^{13}C NMR spectrum (125.5 MHz, $CDCl_3$) of Compound 9 26

Figure S27. CD spectrum Compound 2 27

Figure S28. CD spectrum Compound 3 27

Figure S29. CD spectrum Compound 4 28

Figure S30. CD spectrum Compound 5 28

Figure S31. CD spectrum Compound 6 29

Figure S32. CD spectrum Compound 7 29

Figure S33. CD spectrum Compound 8 30

Figure S34. CD spectrum Compound 9 30

Figure S35. HR (+)ESI MS spectrum of 1 31

Figure S36. HR (+)ESI MS spectrum of 2 31

Figure S37. HR (+)ESI MS spectrum of 3 32

Figure S38. HR (+)ESI MS spectrum of 4 32

Figure S39. HR (+)ESI MS spectrum of 5 33

Figure S40. HR (+)ESI MS spectrum of 6 33

Figure S41. HR (+)ESI MS spectrum of 7 34

Figure S42. HR (+)ESI MS spectrum of 8 34

Figure S43. HR (-)ESI MS spectrum of 9 35

Figure S44. The main conformations of 7*S*,11*S*- and 7*S*,11*R*- stereoisomers of **2**. 36

Figure S45. The potential energy profile for internal rotation of the 3-O–H group around C(3)–OH bond for compound 3. 36

Figure S46. The ECD spectra, calculated for 3*S* stereoisomer of compound 3 as a function of different large-amplitude motions. 37

Figure S47. ECD spectra (Δε) and potential energy function (V) for the internal rotation of OH_{α} - group in 3*S* stereoisomer of compound 3. 38

Figure S48. The calculated basis set dependence of ECD spectra of 3S stereoisomer of compound 3. 38

Figure S1. ^{1}H **NMR spectrum (500 MHz,** $CDCl_3$**) for Compound 1**

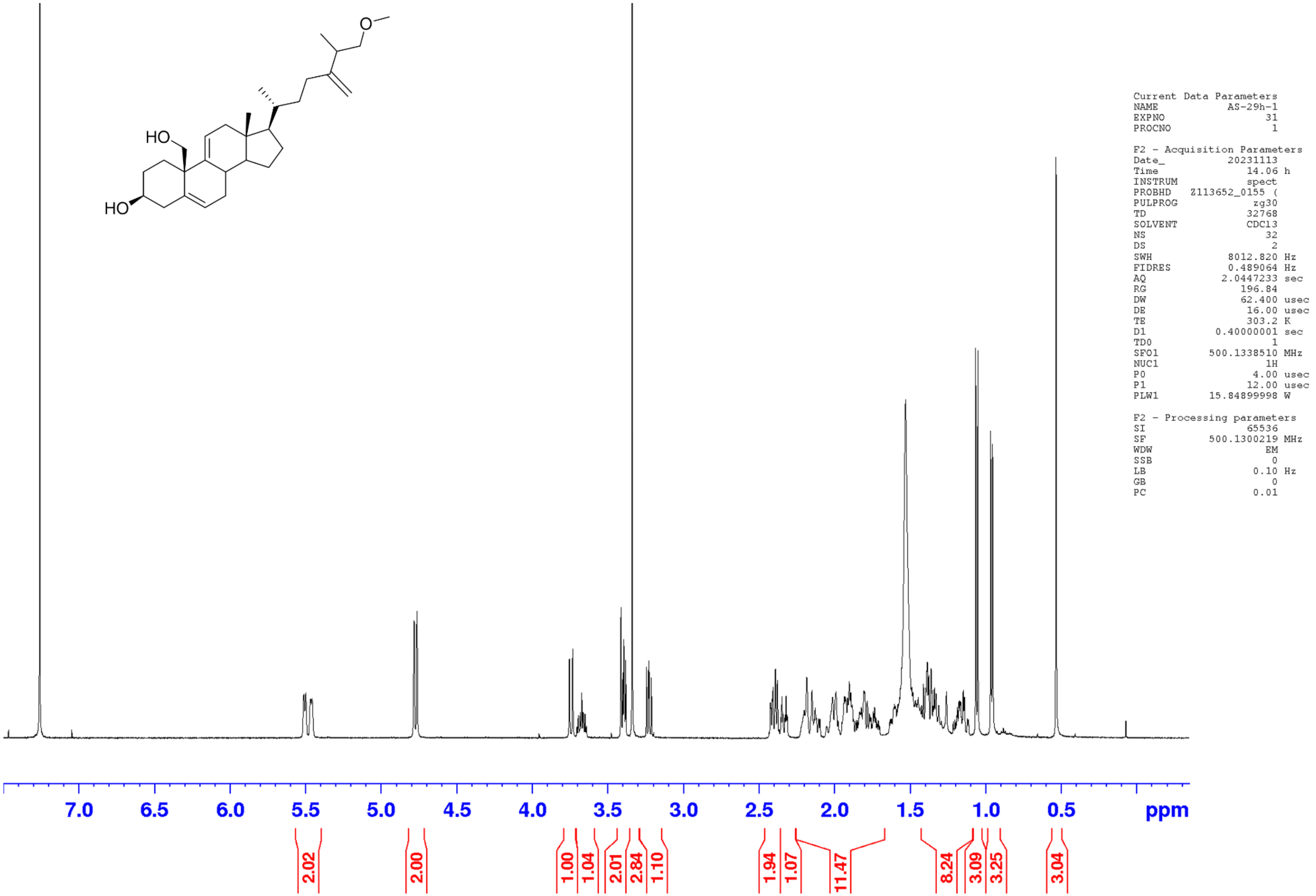

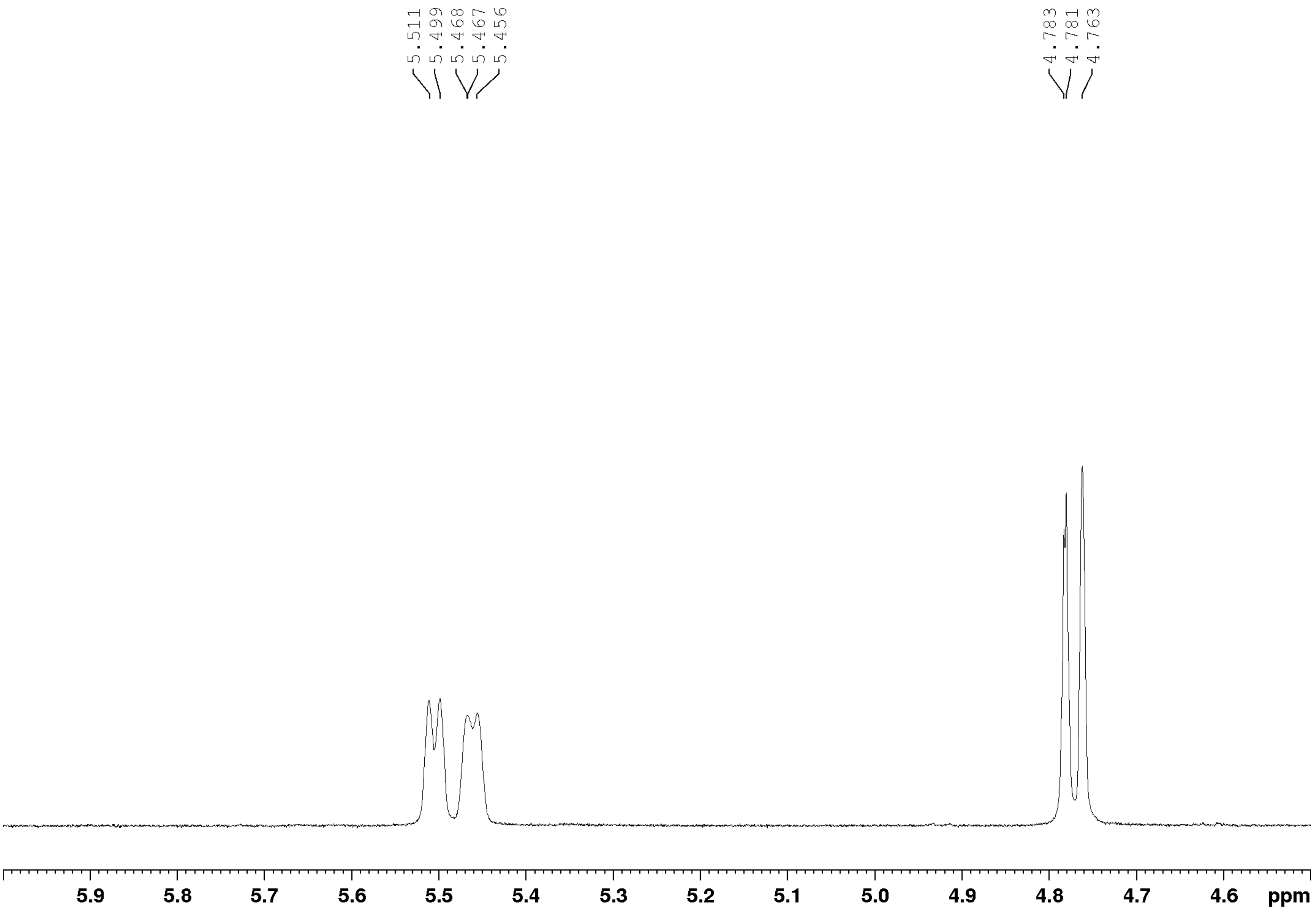
5.511
5.499
5.468
5.467
5.456
4.783
4.781
4.763
5.9
5.8
5.7
5.6
5.5
5.4
5.3
5.2
5.1
5.0
4.9
4.8
4.7
4.6
ppm

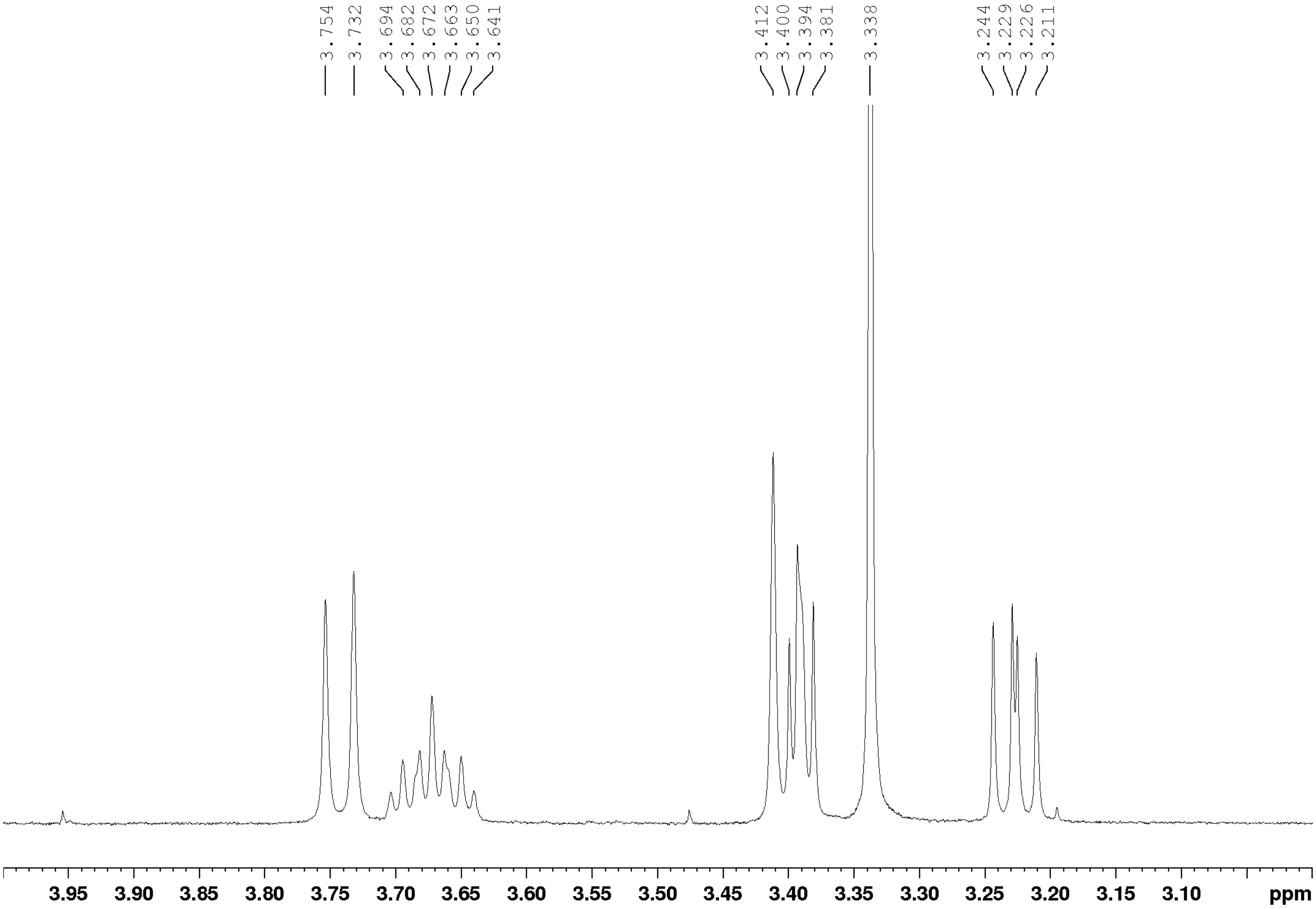
3.754
3.732
3.694
3.682
3.672
3.663
3.650
3.641
3.412
3.400
3.394
3.381
3.338
3.244
3.229
3.226
3.211
3.95 3.90 3.85 3.80 3.75 3.70 3.65 3.60 3.55 3.50 3.45 3.40 3.35 3.30 3.25 3.20 3.15 3.10 ppm

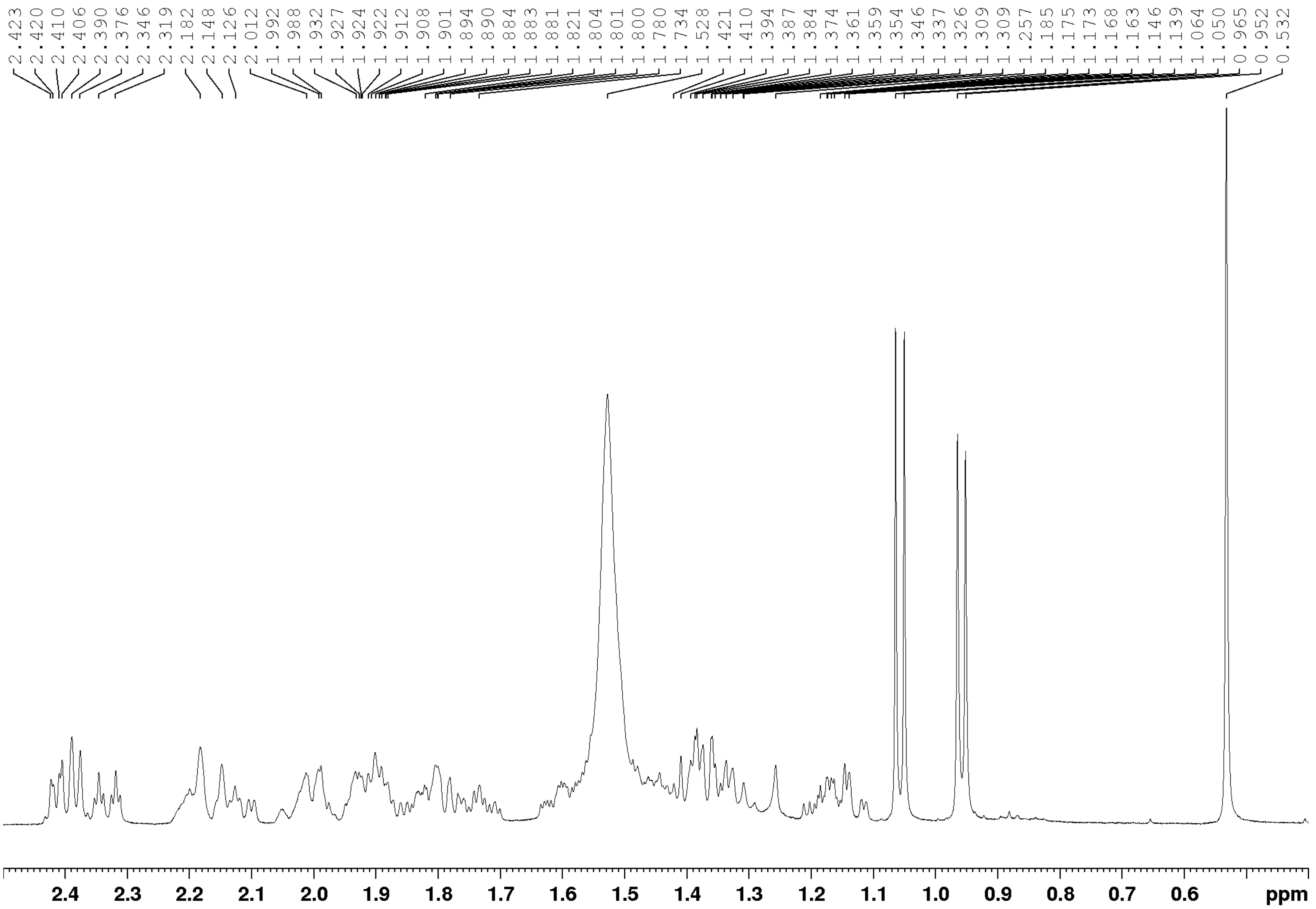
2.423
2.420
2.410
2.406
2.390
2.376
2.346
2.319
2.182
2.148
2.126
2.012
1.992
1.988
1.932
1.927
1.924
1.922
1.912
1.908
1.901
1.894
1.890
1.884
1.883
1.881
1.821
1.804
1.801
1.800
1.780
1.734
1.528
1.421
1.410
1.394
1.387
1.384
1.374
1.361
1.359
1.354
1.346
1.337
1.326
1.309
1.309
1.257
1.185
1.175
1.173
1.168
1.163
1.146
1.139
1.064
1.050
0.965
0.952
0.532
2.4
2.3
2.2
2.1
2.0
1.9
1.8
1.7
1.6
1.5
1.4
1.3
1.2
1.1
1.0
0.9
0.8
0.7
0.6
ppm

Figure S2. ^{13}C NMR spectrum (125.5 MHz, $CDCl_3$) of Compound 1

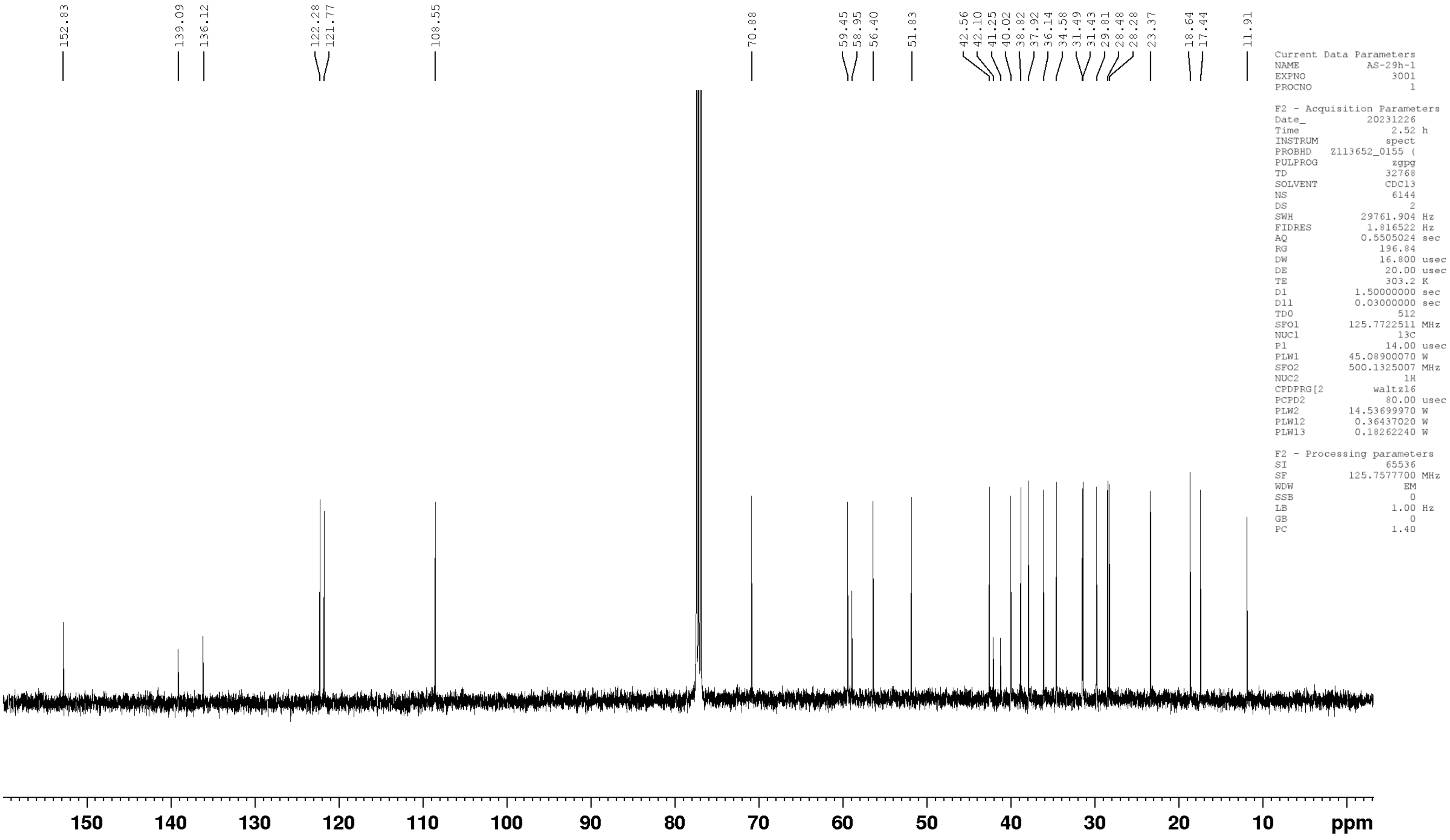

Figure S3. DEPT-135 spectrum (500 MHz, $CDCl_3$) of Compound 1

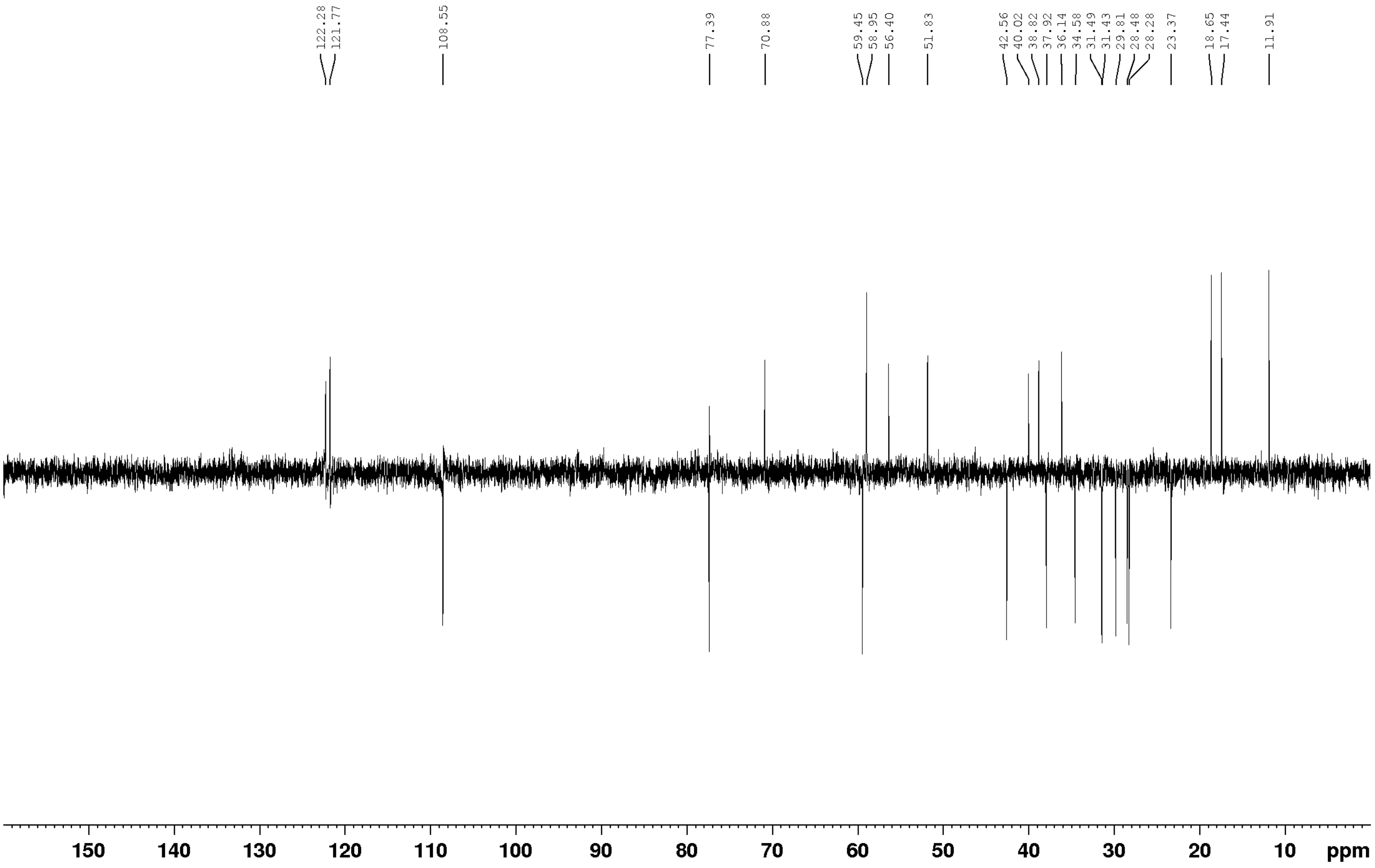

Figure S4. HSQC spectrum (500 MHz, $CDCl_3$) of Compound 1

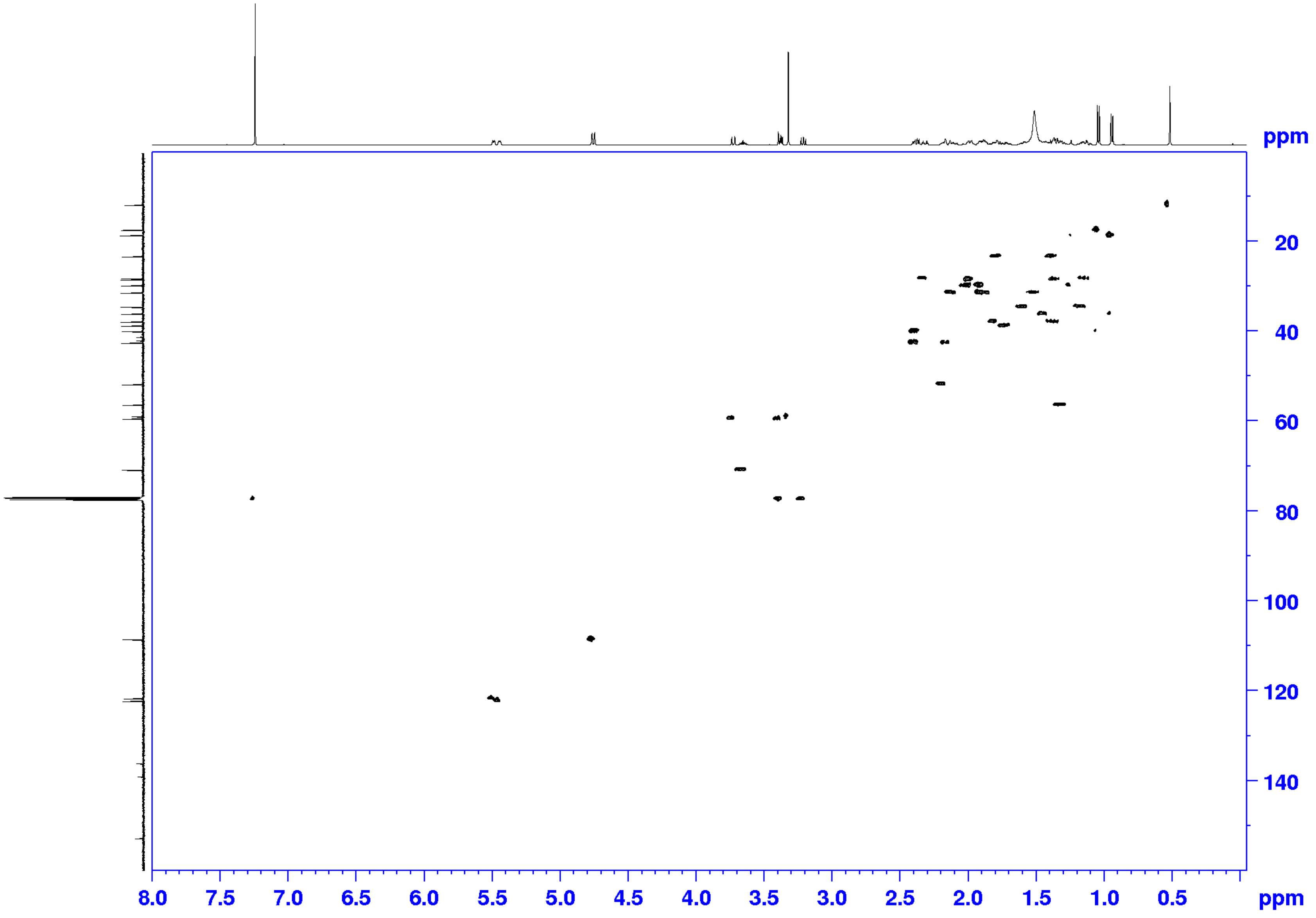

Figure S5. ^{1}H-^{1}H COSY spectrum (500 MHz, $CDCl_3$) of Compound 1

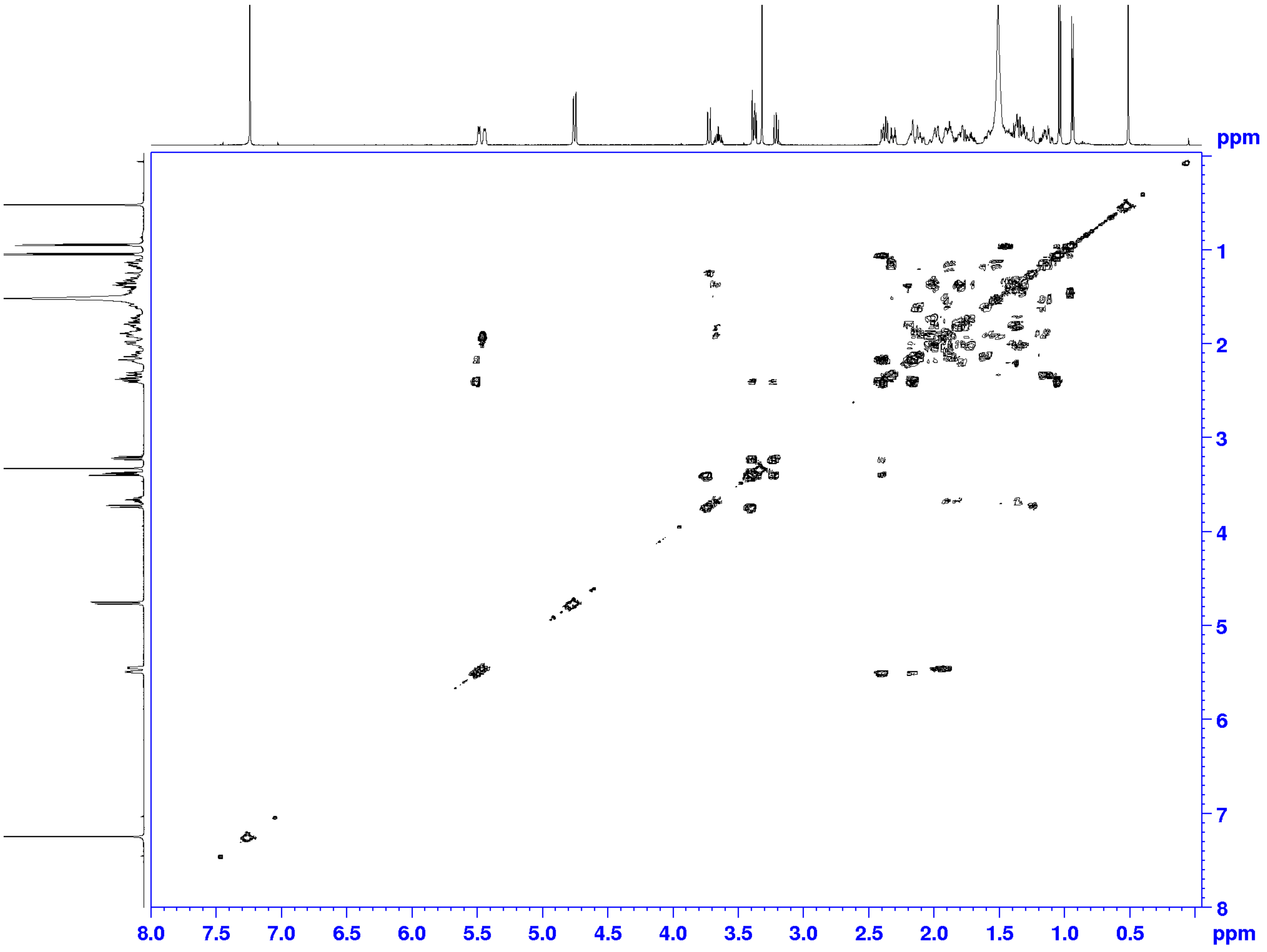

Figure S6. HMBC spectrum (500 MHz, $CDCl_3$) of Compound 1

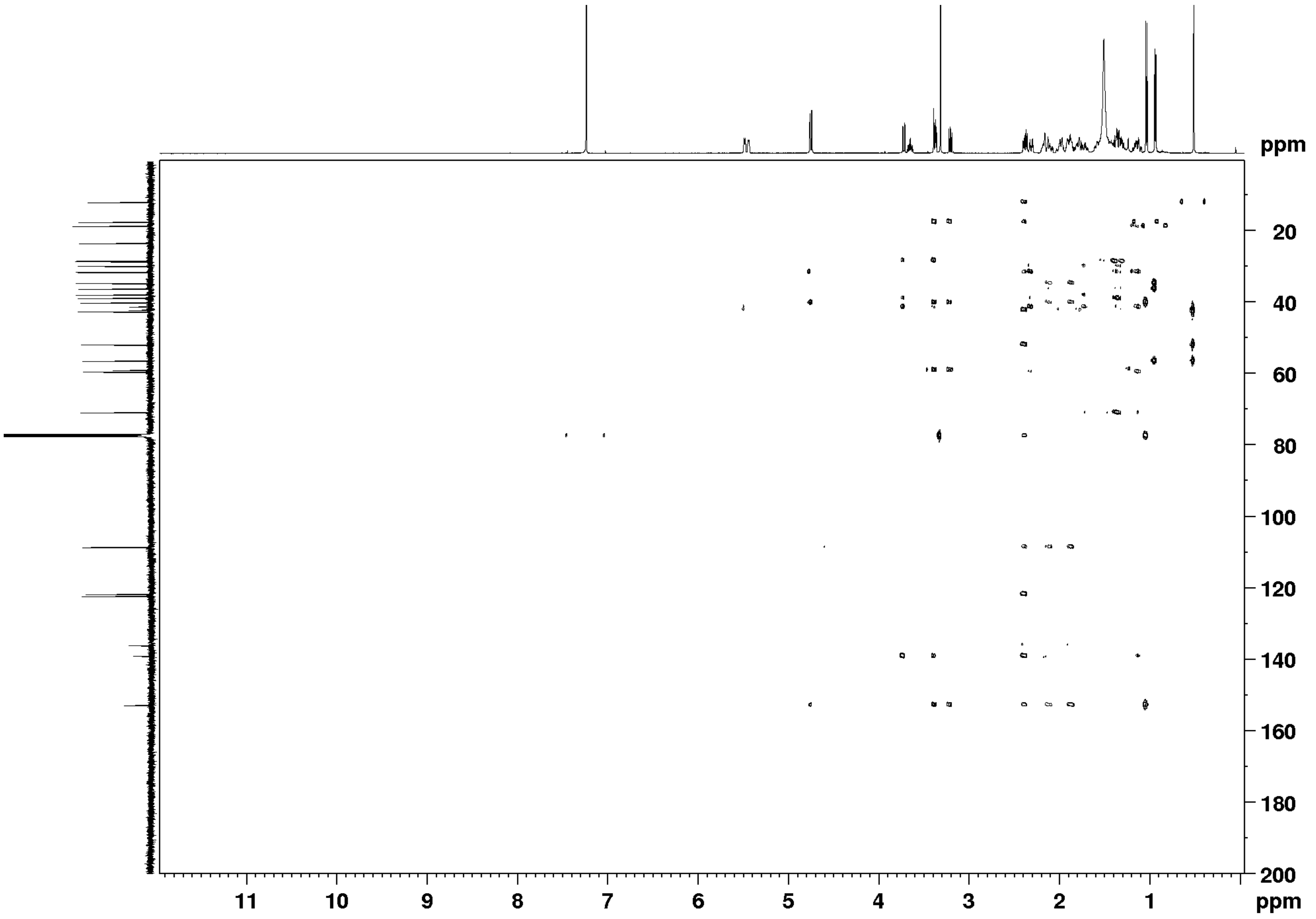

Figure S7. ^{13}**C NMR spectrum (125.5 MHz, CDCl$_3$) of Compound 2**

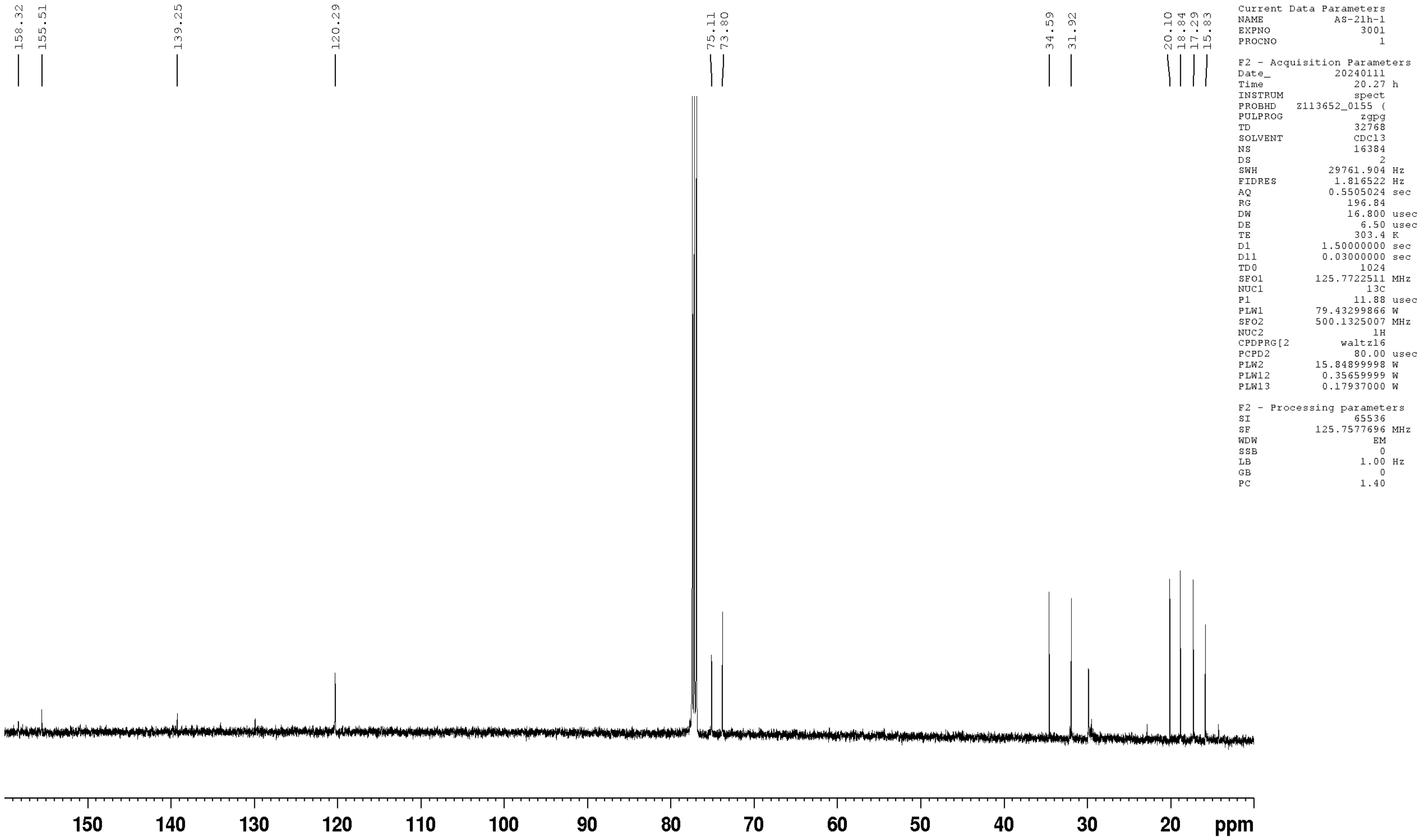

Figure S8. DEPT-135 spectrum (500 MHz, $CDCl_3$) of Compound 2

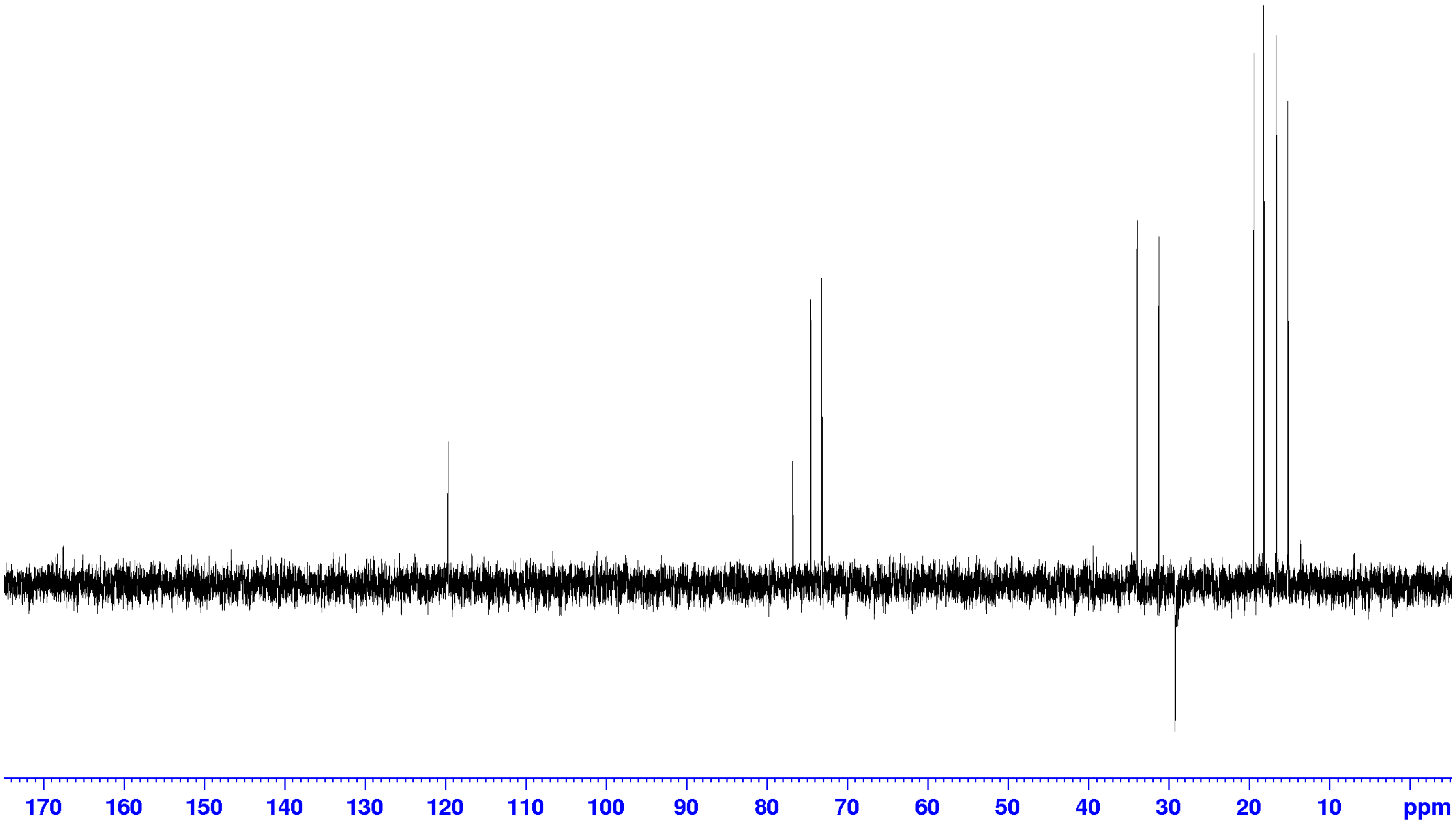

Figure S9. ^{1}H NMR spectrum (500 MHz, $CDCl_3$) of Compound 2

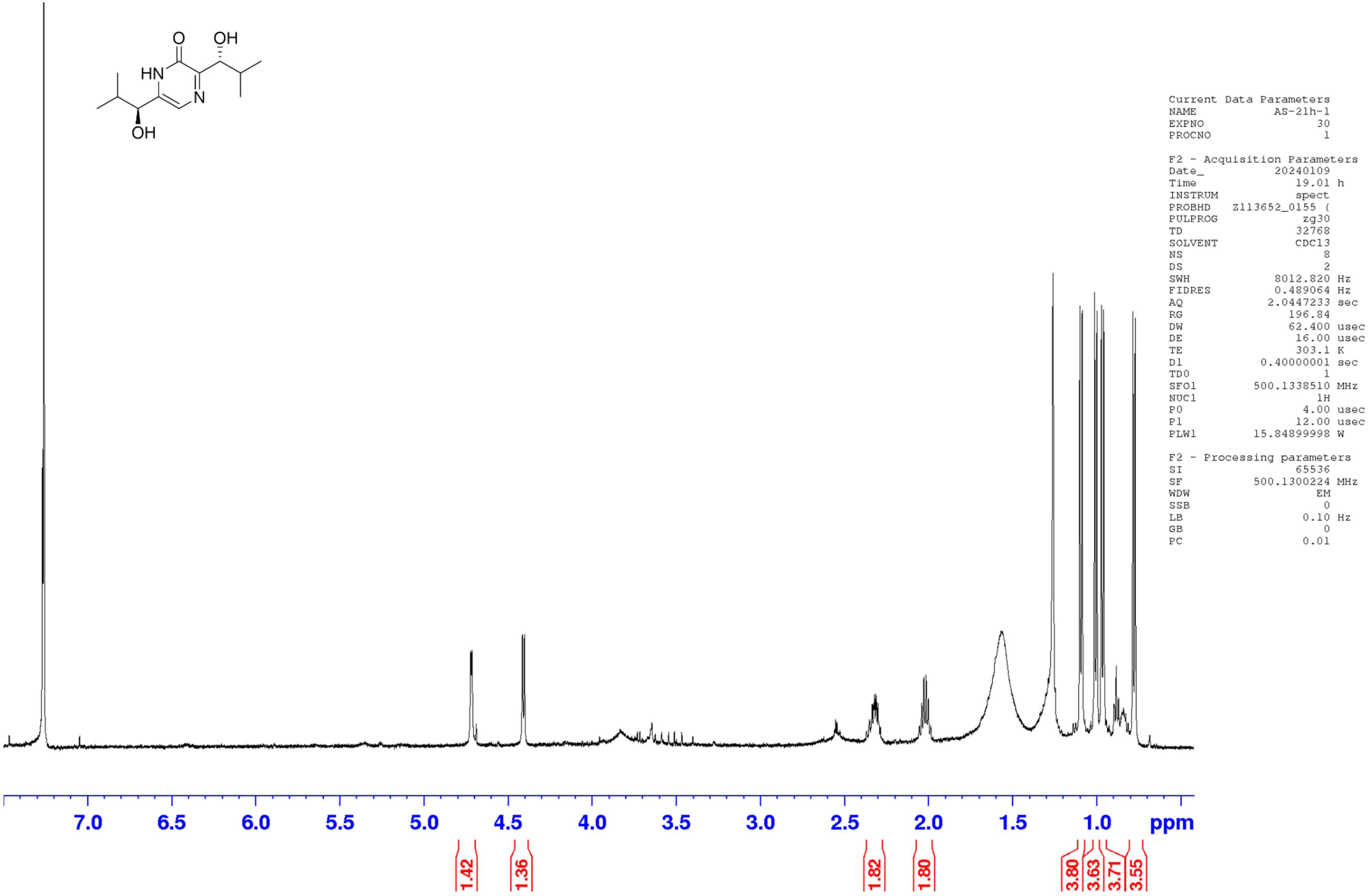

Figure S10. ^{1}H-^{1}H COSY spectrum (500 MHz, $CDCl_3$) of Compound 2

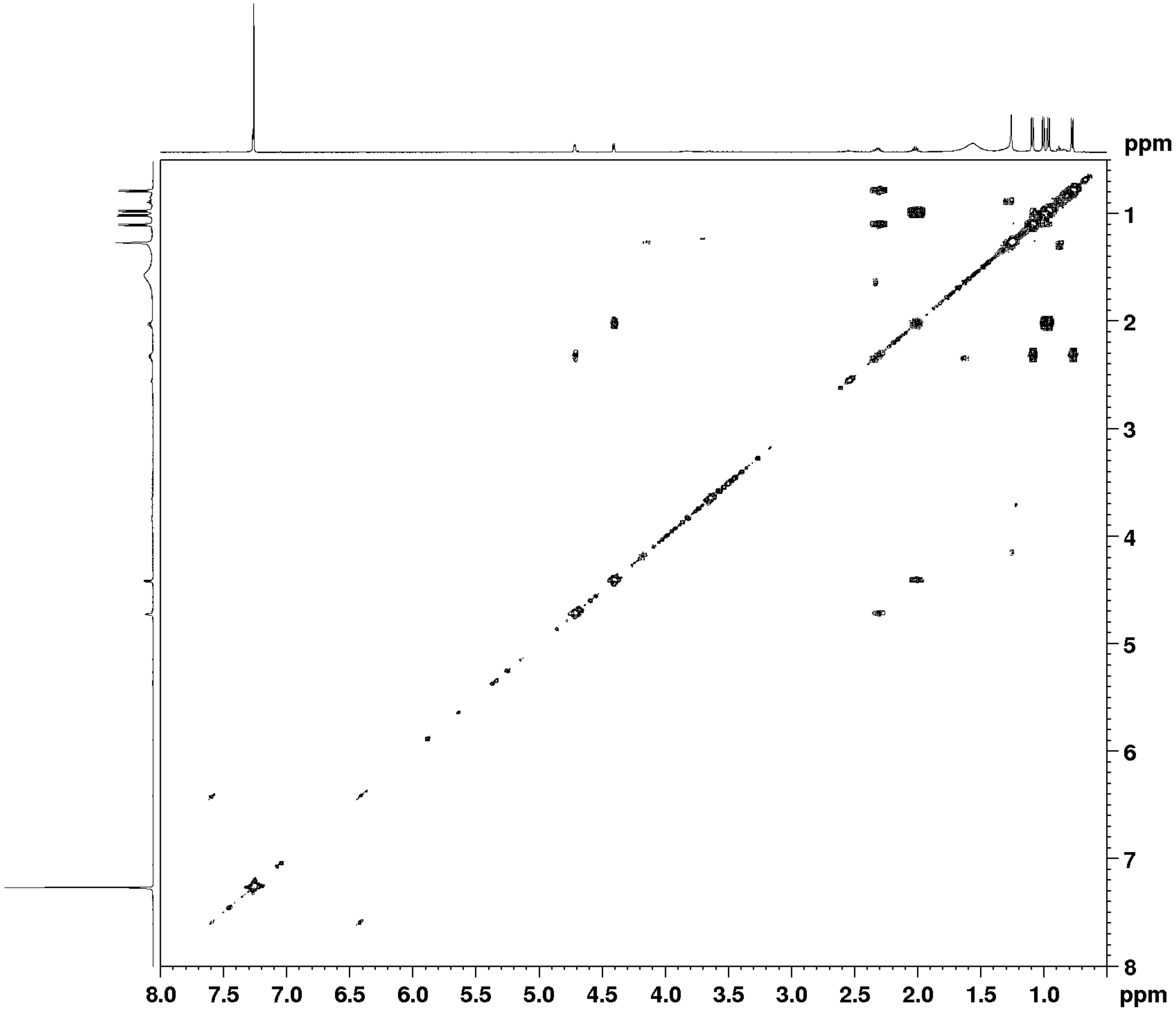

Figure S11. HSQC spectrum (500 MHz, $CDCl_3$) of Compound 2

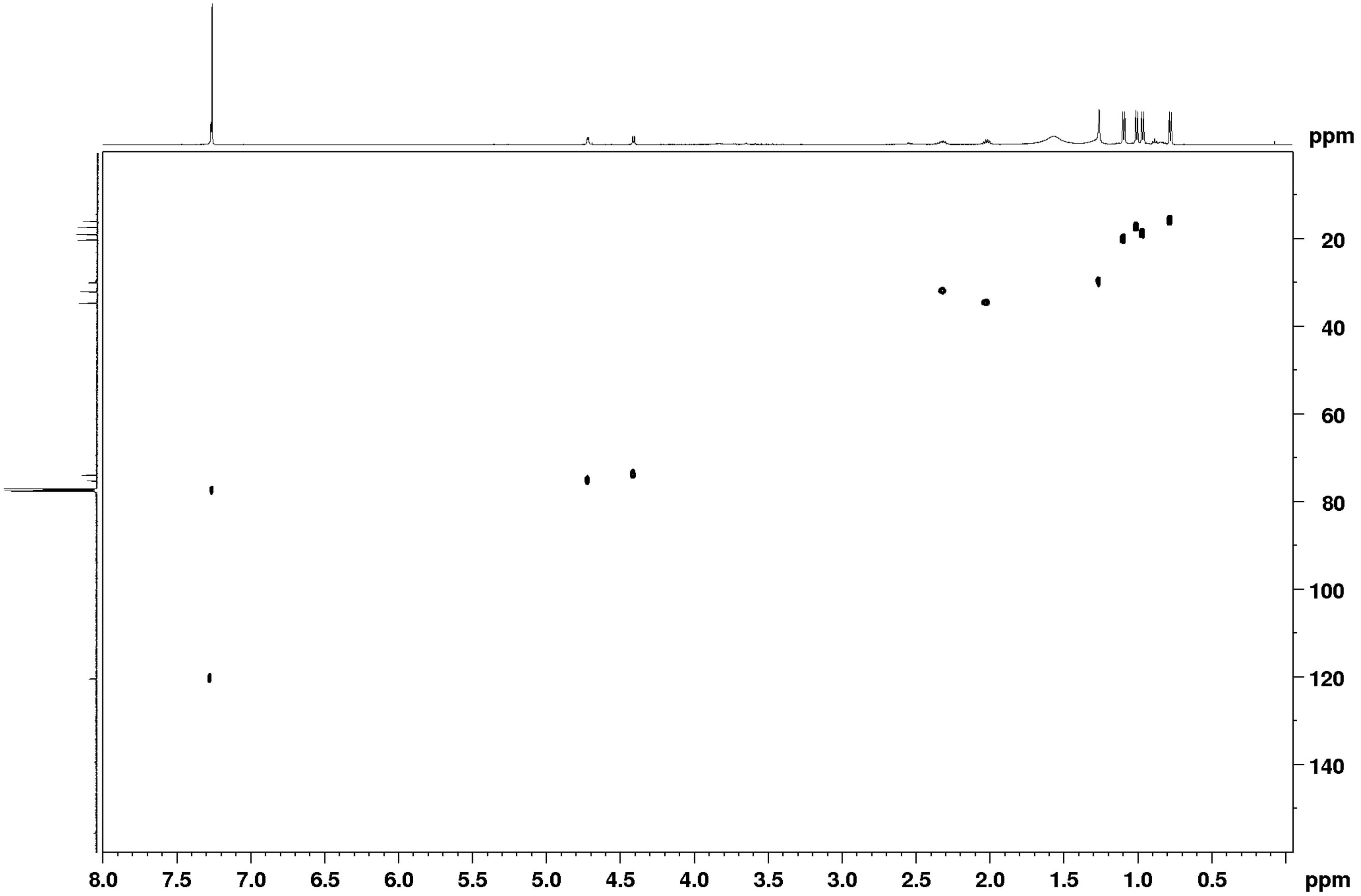

Figure S12. HMBC spectrum (500 MHz, $CDCl_3$) of Compound 2

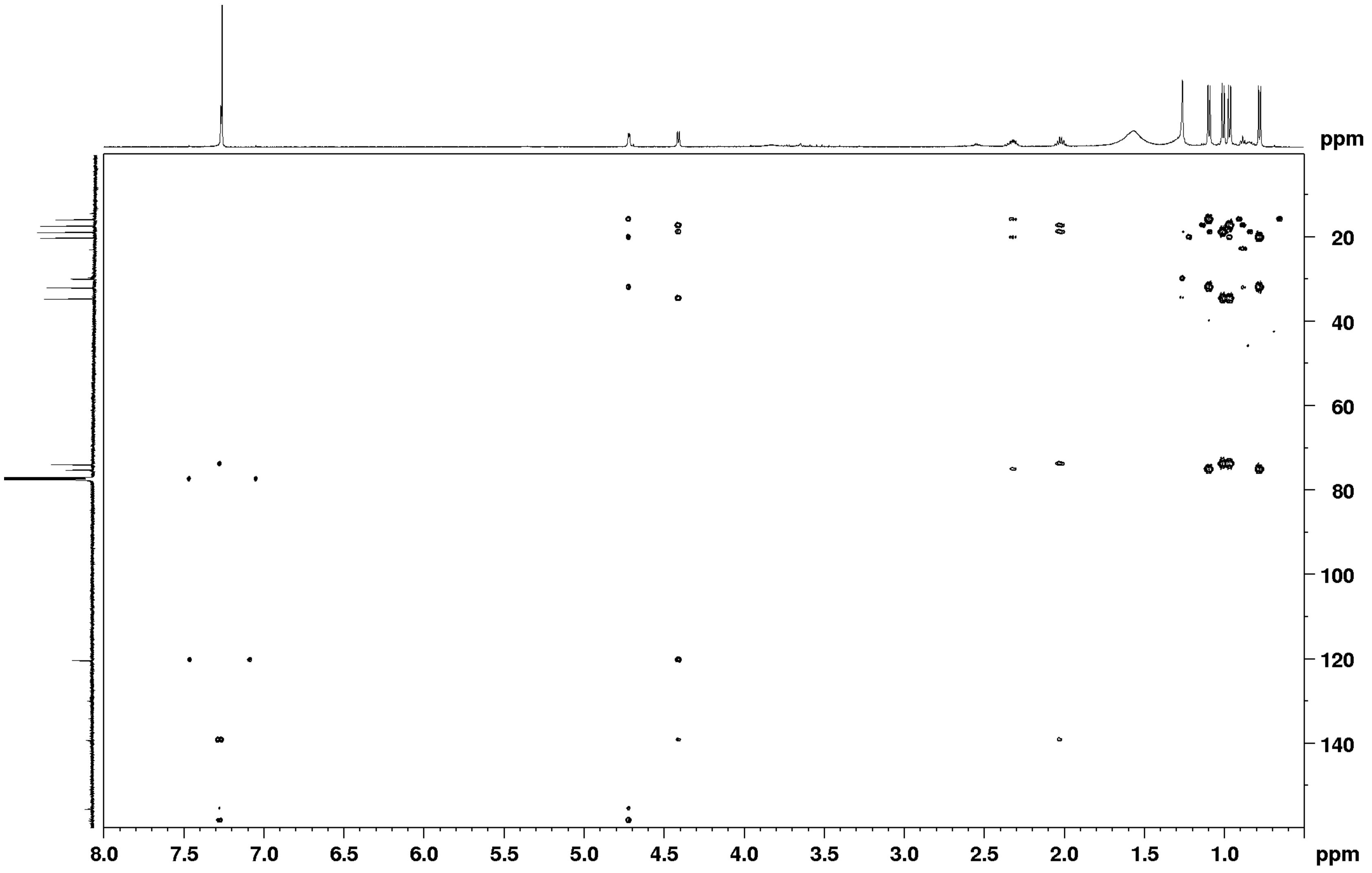

Figure S13. ^{1}H NMR spectrum (500 MHz, $CDCl_3$) for Compound 3

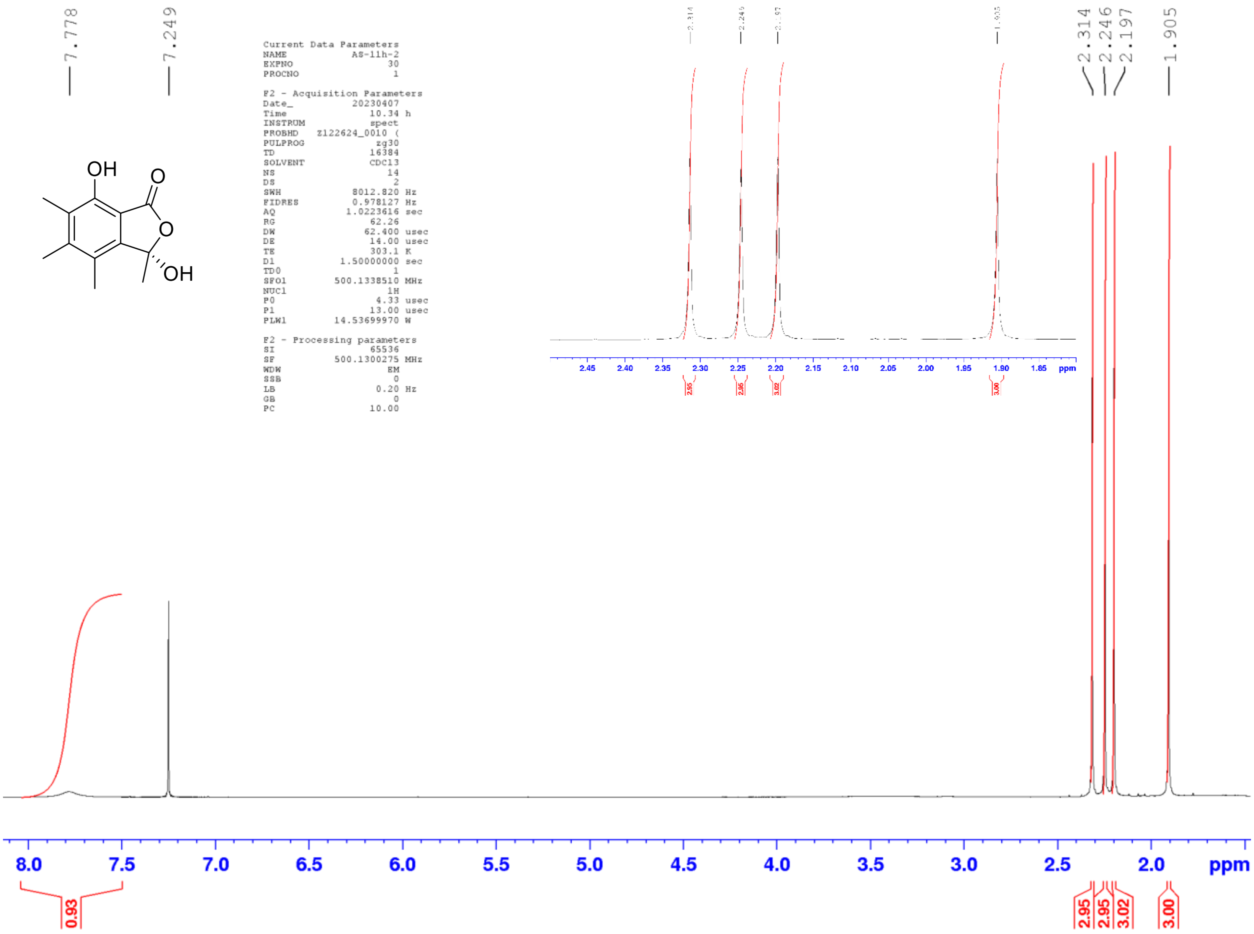

Figure S14. ^{13}C NMR spectrum (125.5 MHz, $CDCl_3$) of Compound 3

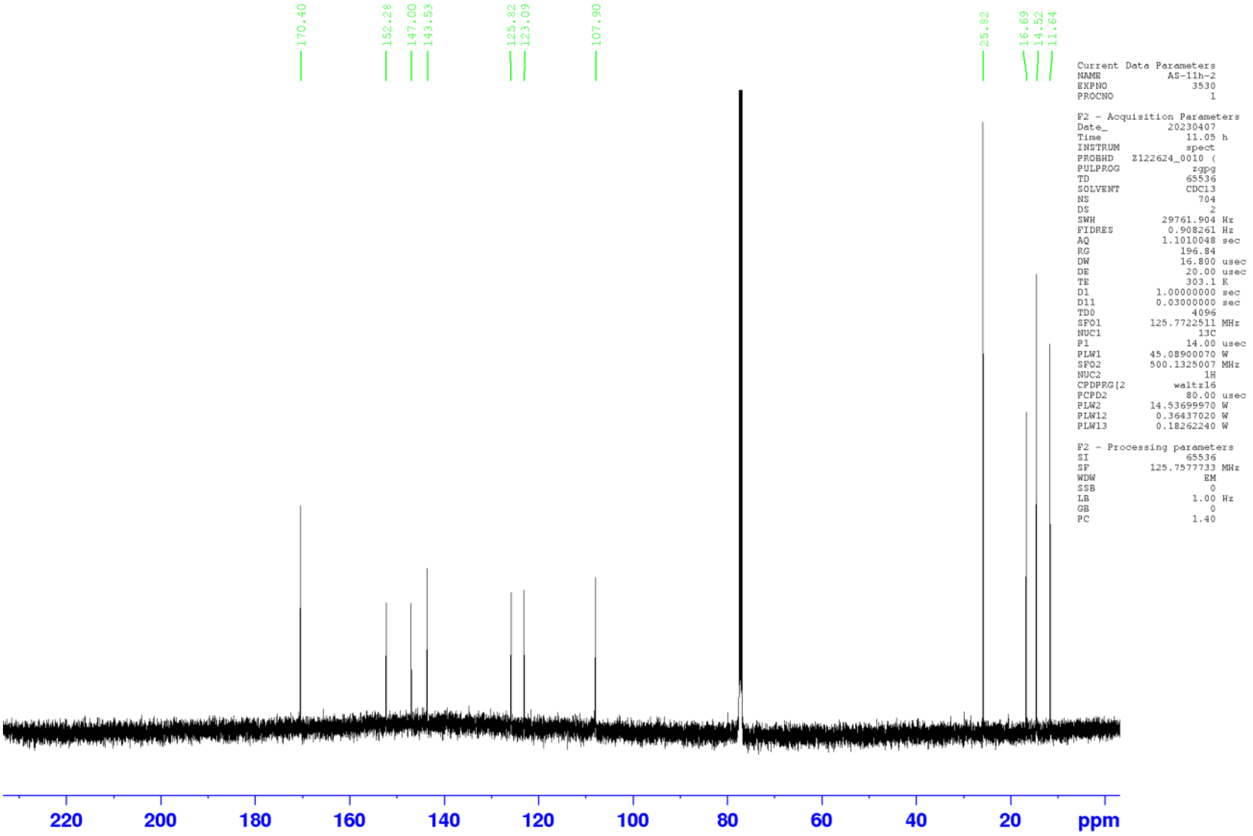

Figure S15. ^{1}H NMR spectrum (500 MHz, $CDCl_3$) for Compound 4

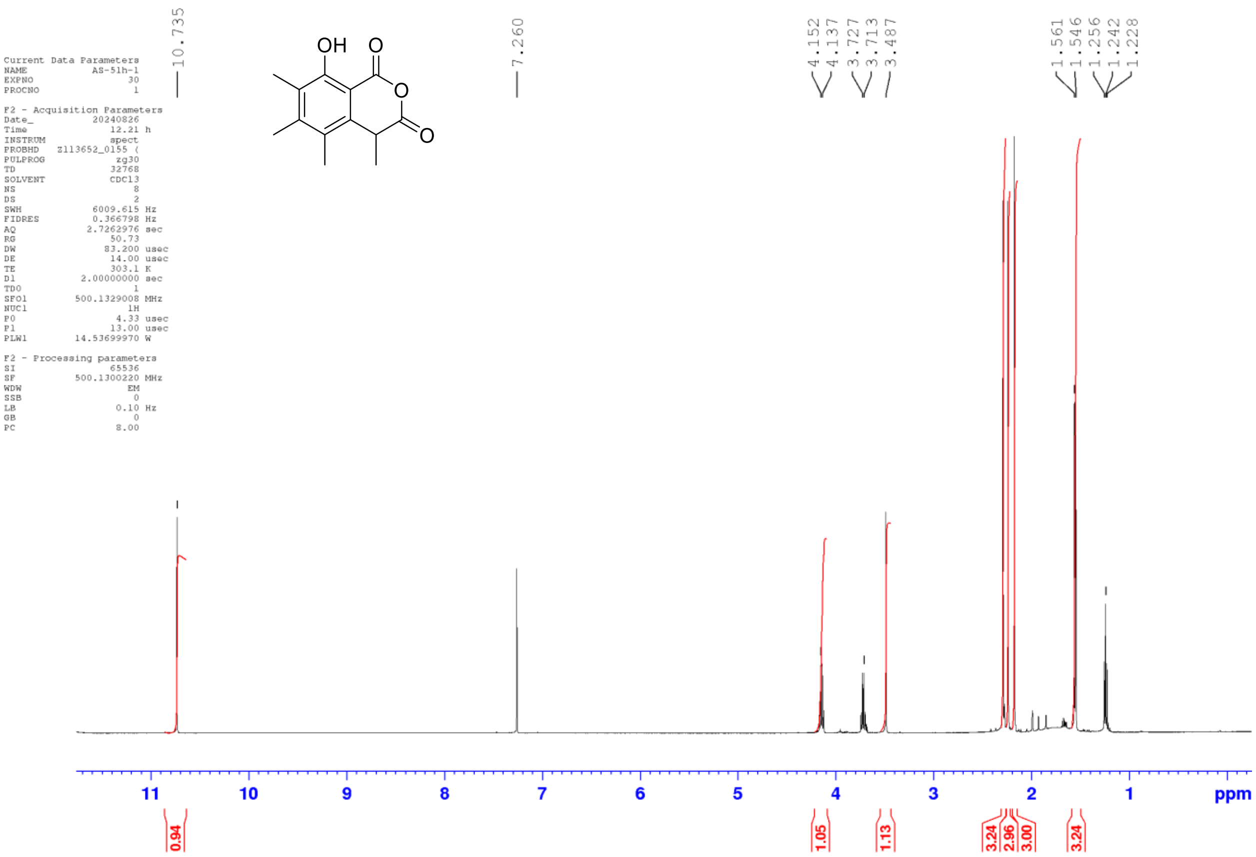

Figure S16. ^{13}C NMR spectrum (125.5 MHz, $CDCl_3$) of Compound 4

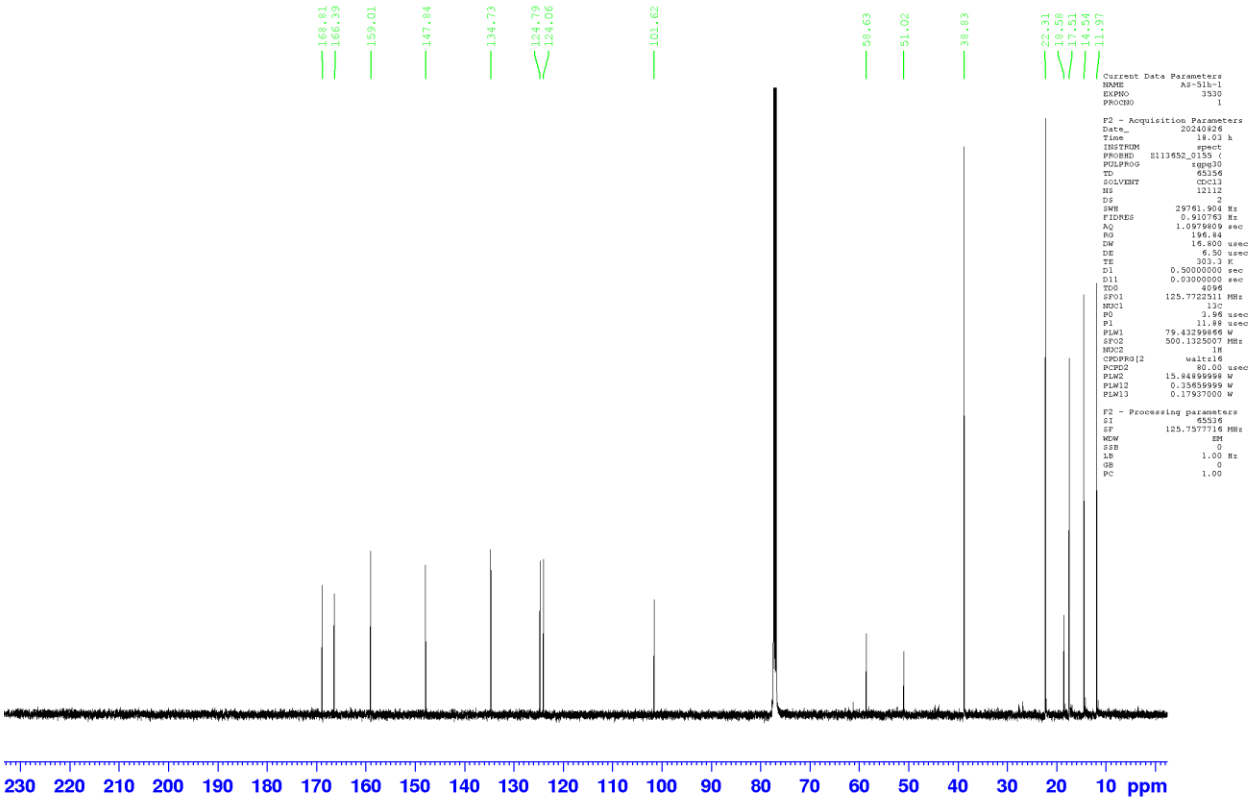

Figure S17. ^{1}H NMR spectrum (500 MHz, CD_3OD) for Compound 5

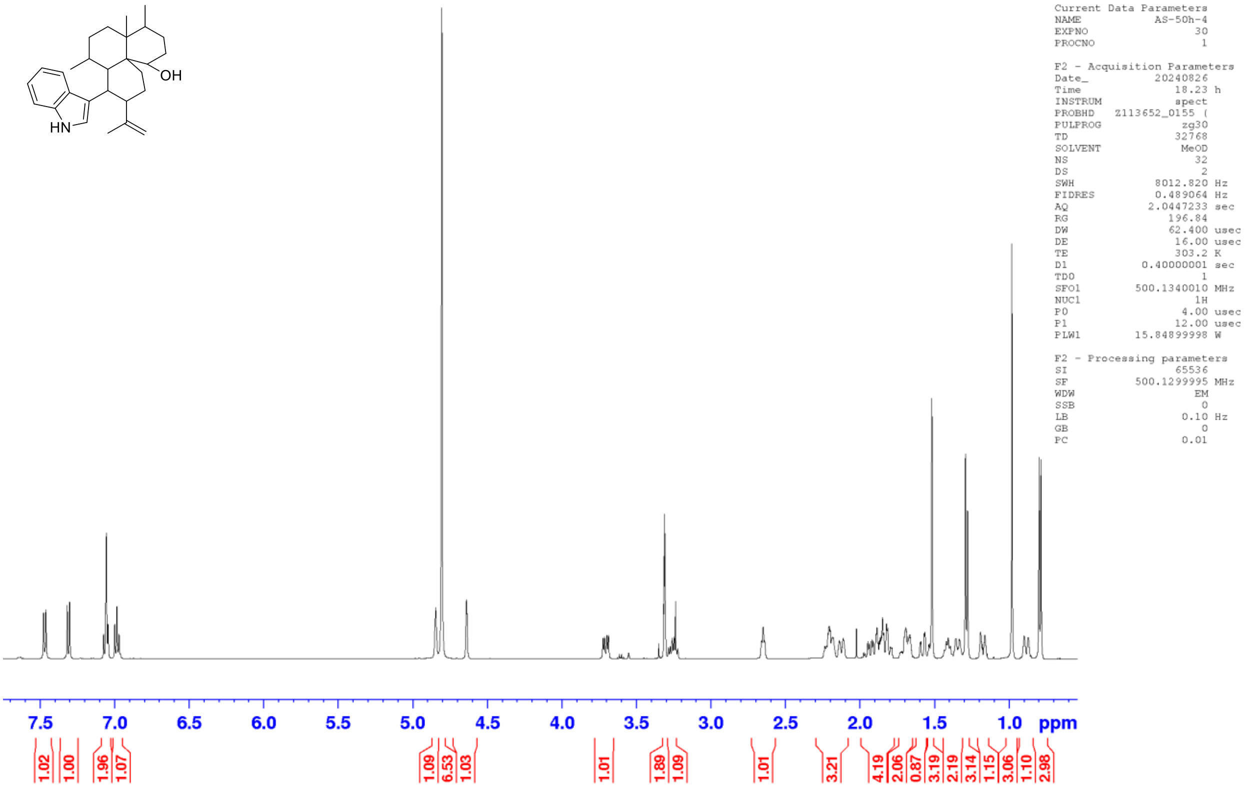

Figure S18. ^{13}C NMR spectrum (125.5 MHz, CD_3OD) of Compound 5

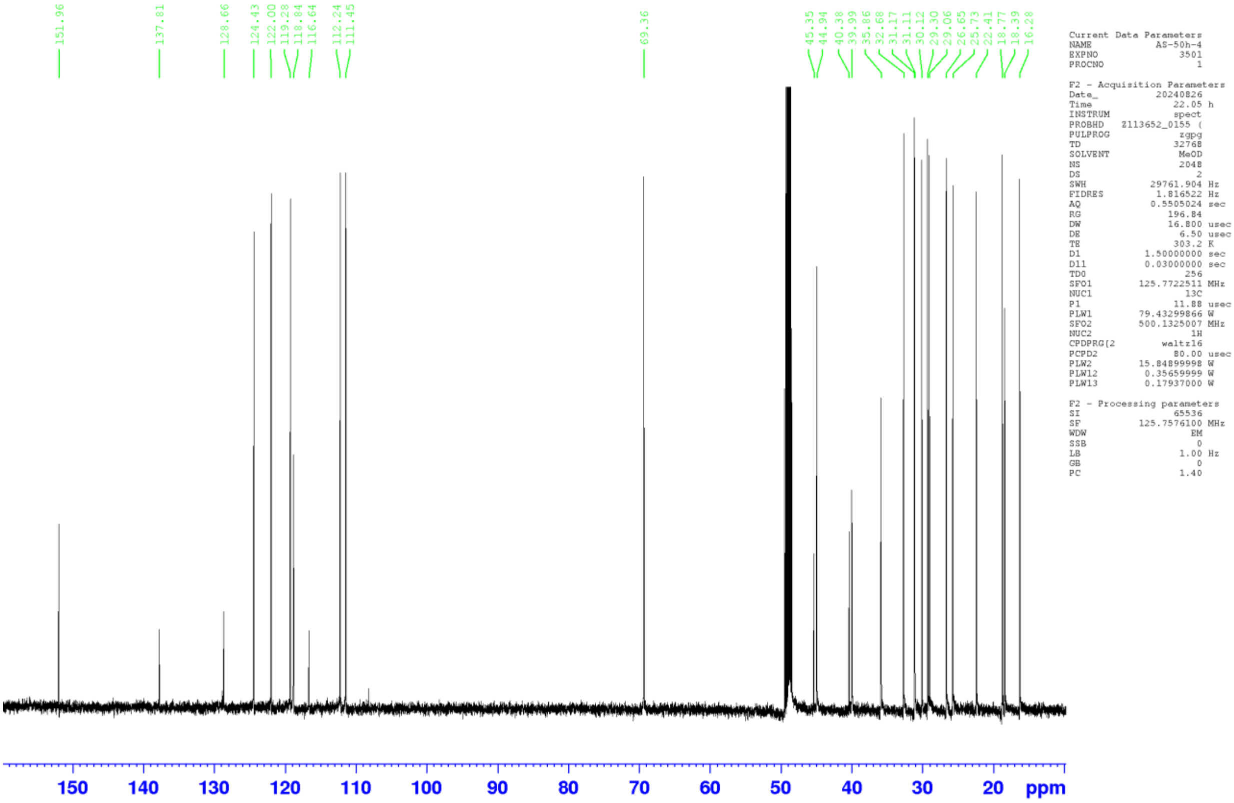

Figure S19. ^{1}**H NMR spectrum (500 MHz, DMSO) for Compound 6**

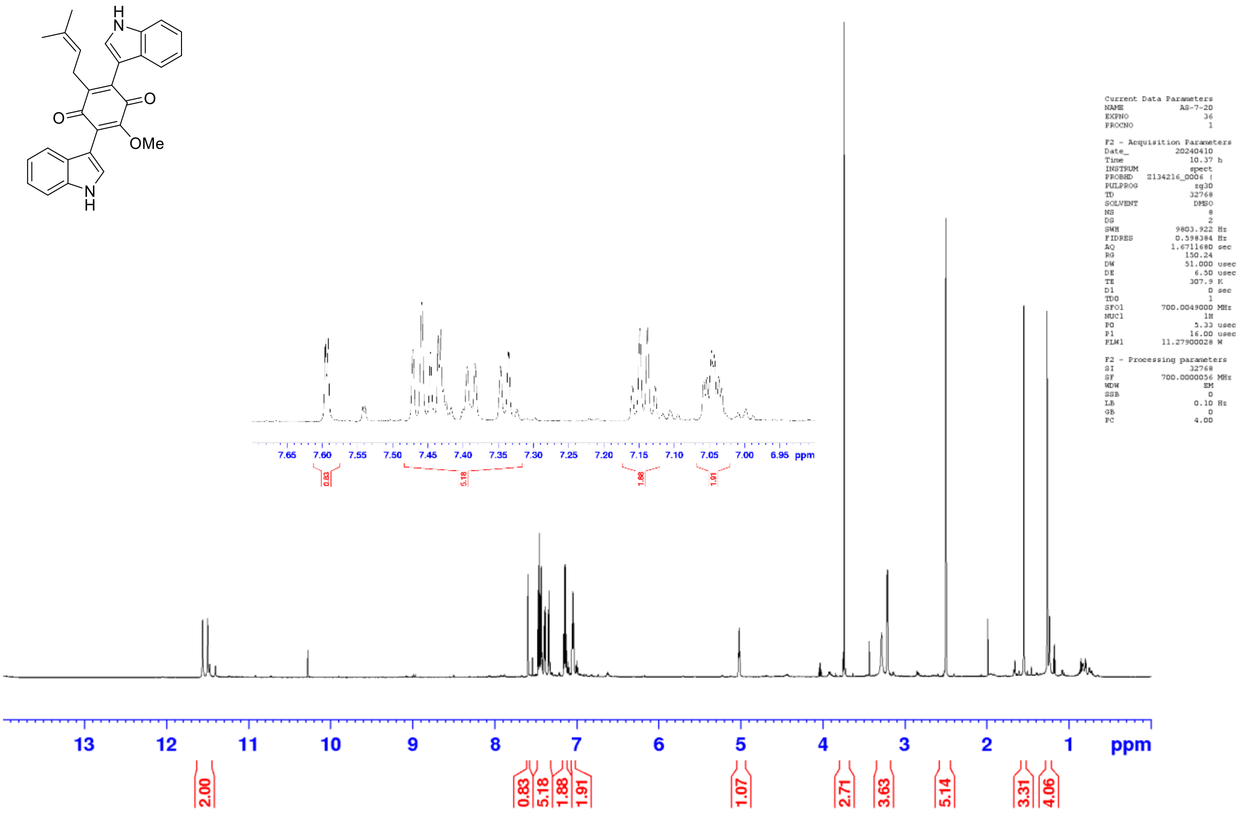

Figure S20. ^{13}**C NMR spectrum (125.5 MHz, DMSO) of Compound 6**

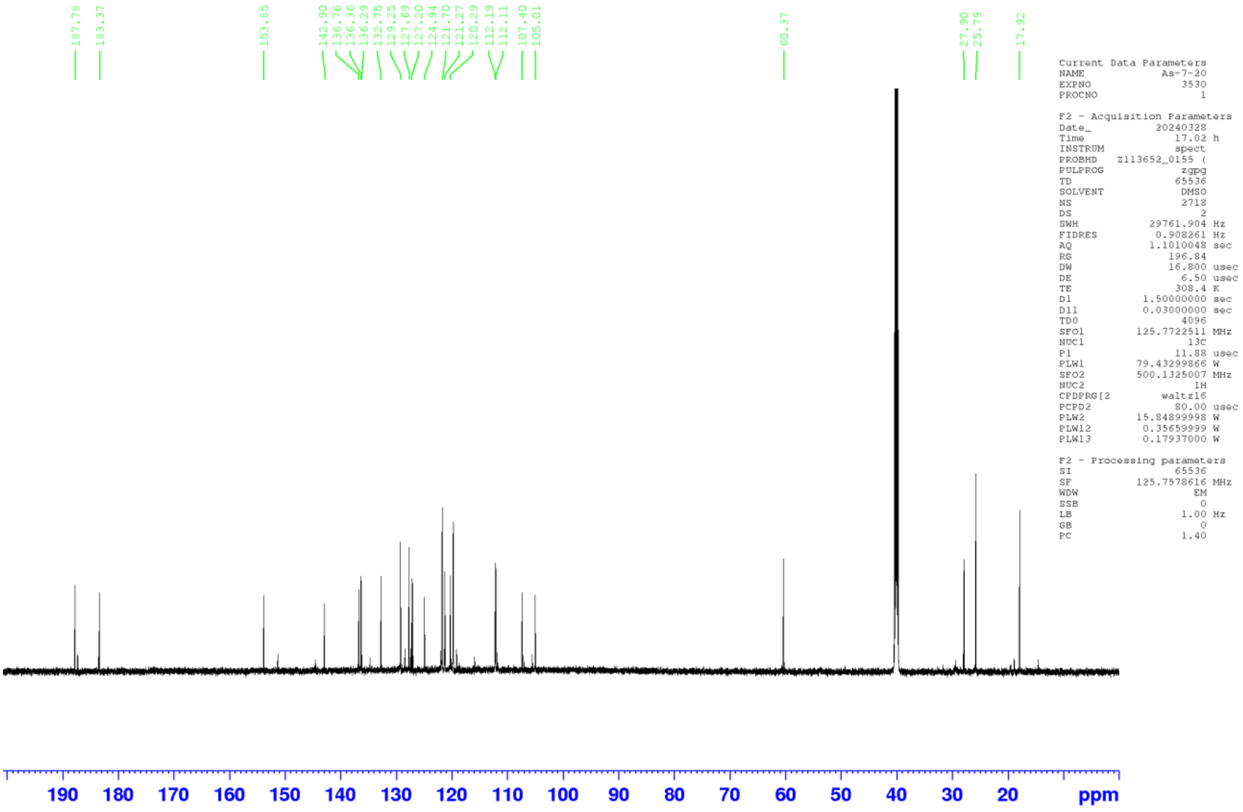

Figure S21. ^{1}H NMR spectrum (500 MHz, DMSO) for Compound 7

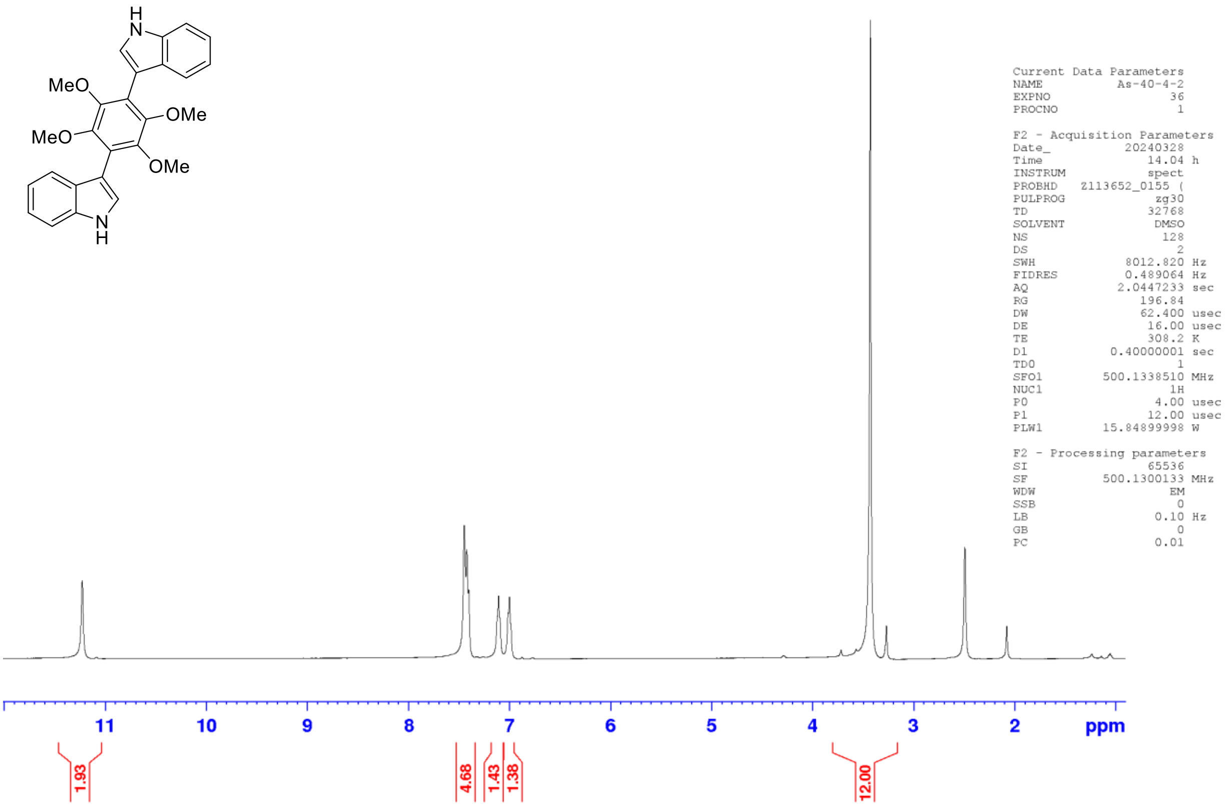

Figure S22. ^{13}C NMR spectrum (125.5 MHz, DMSO) of Compound 7

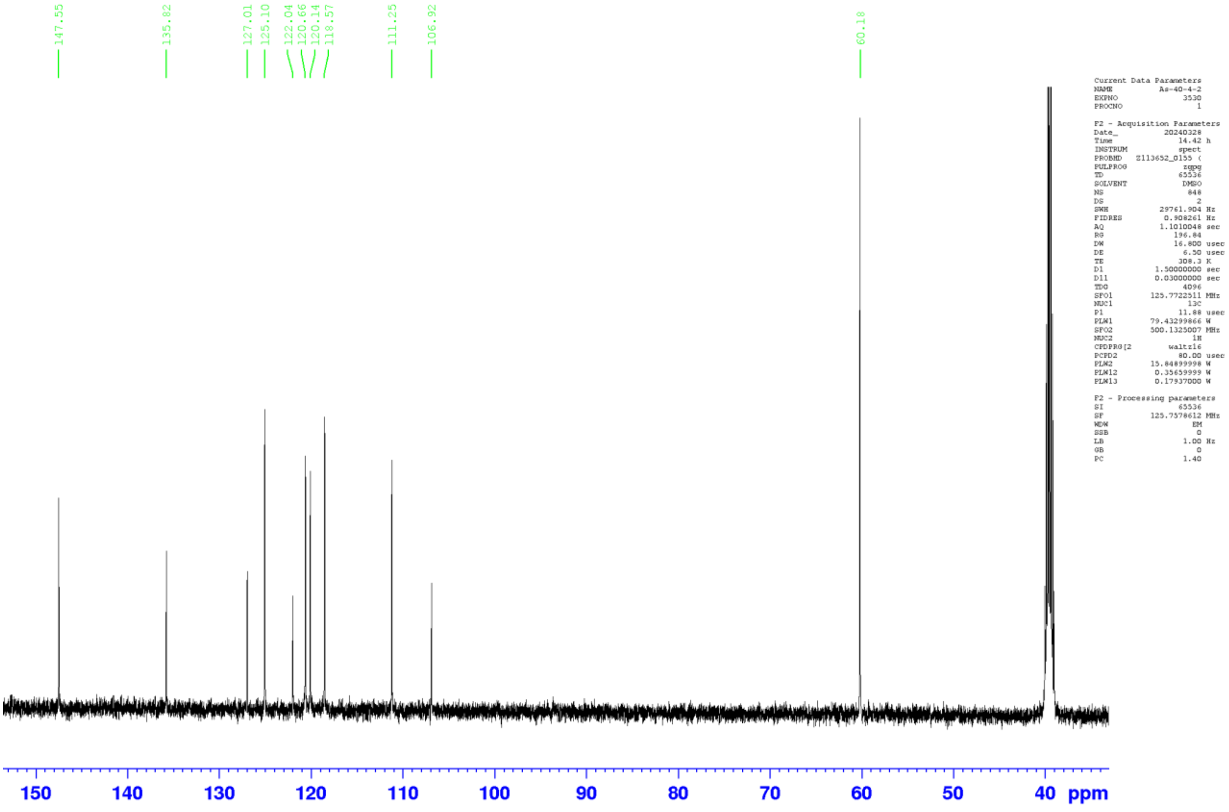

Figure S23. ^{1}**H NMR spectrum (500 MHz, acenote$_{d6}$) for Compound 8**

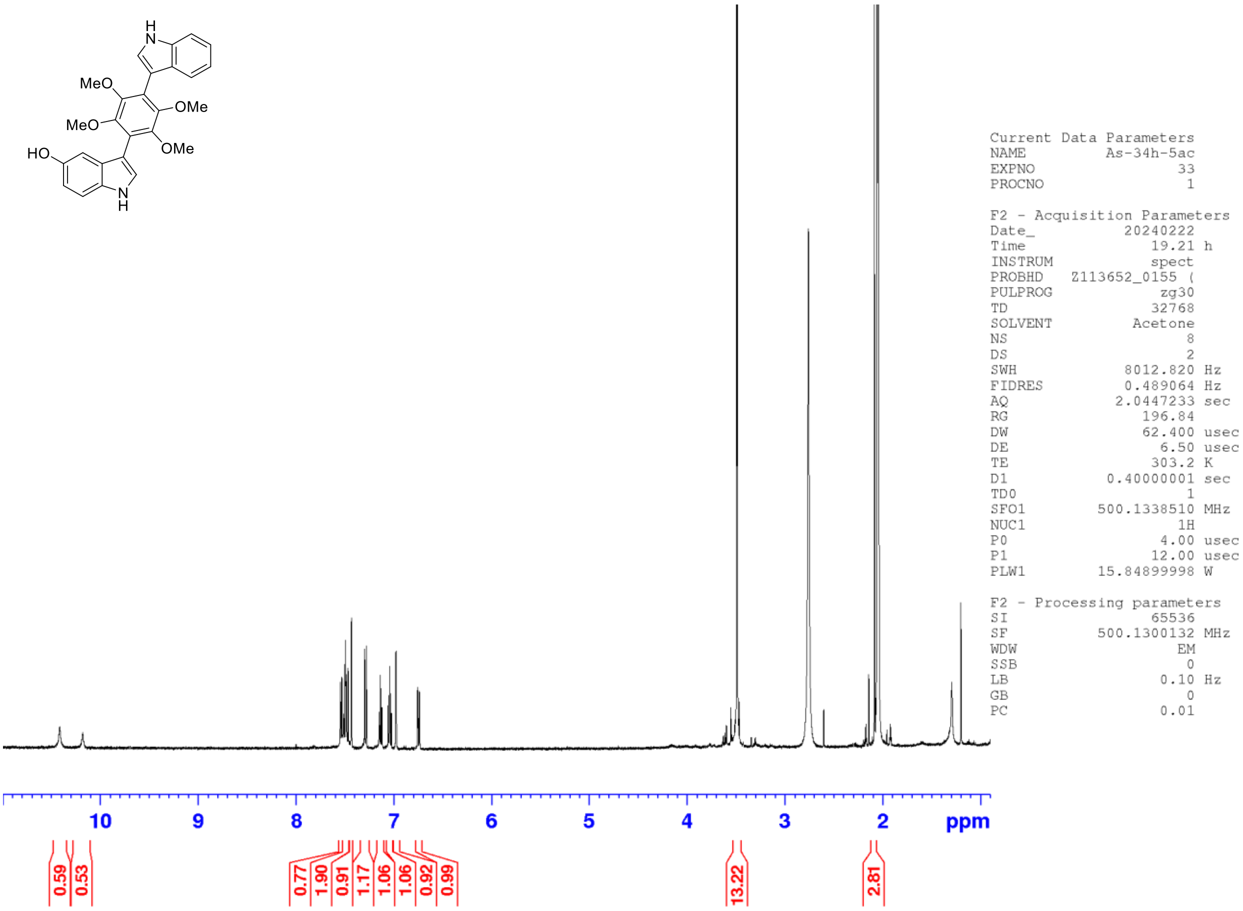

Figure S24. ^{13}**C NMR spectrum (125.5MHz, acenote$_{d6}$) of Compound 8**

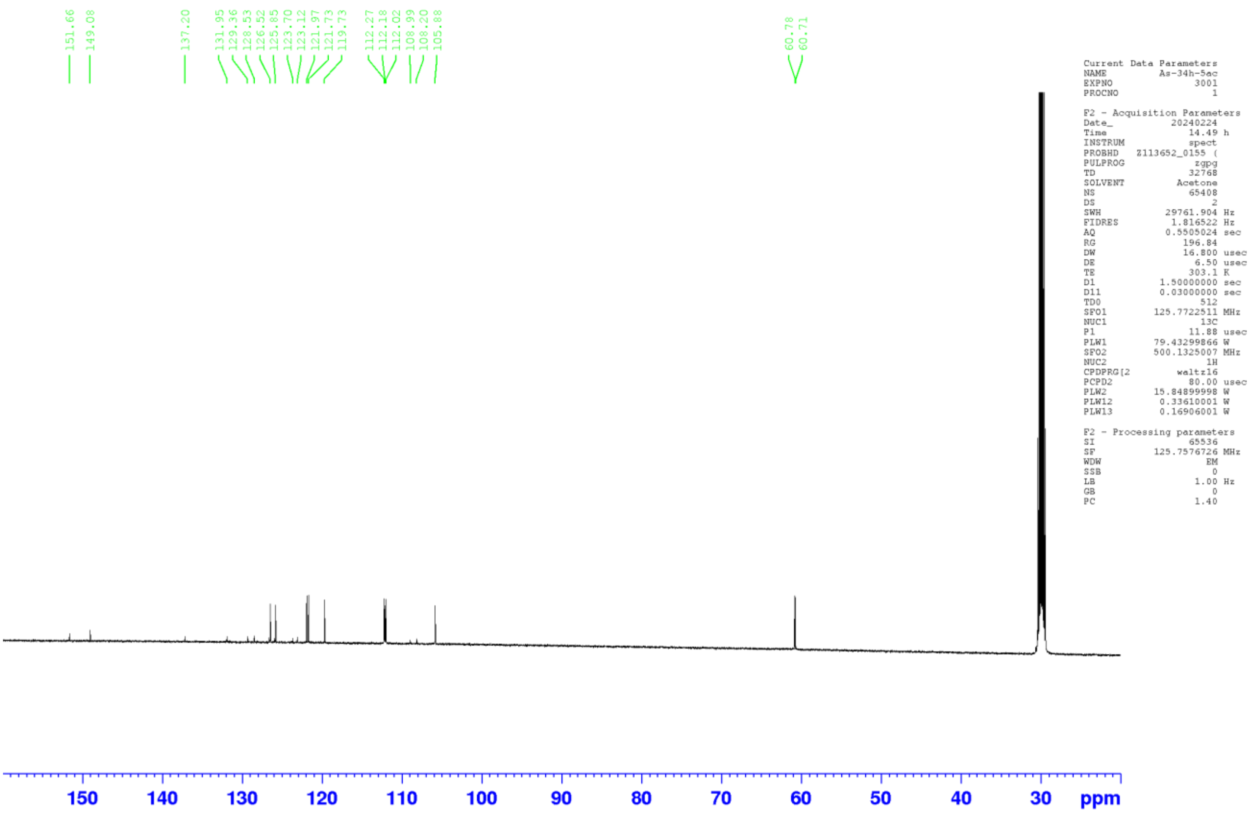

Figure S25. ^{1}H NMR spectrum (500 MHz, $CDCl_3$) for Compound 9

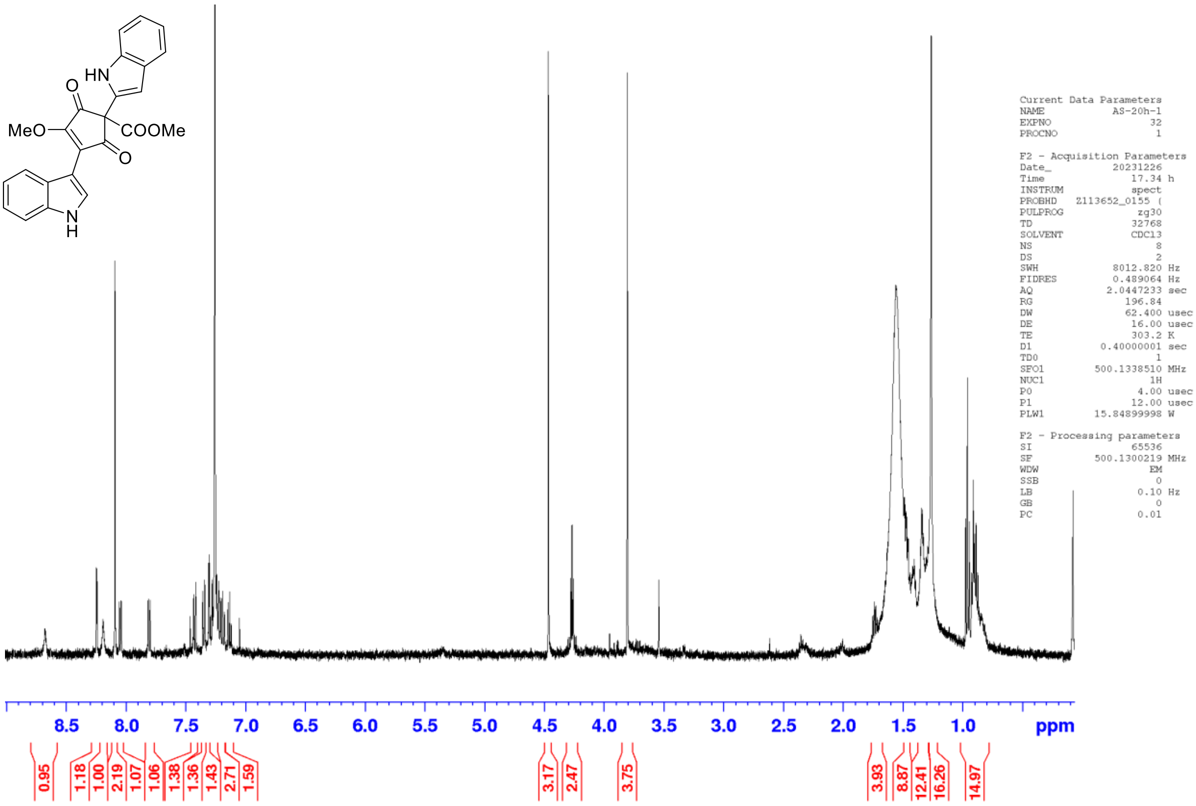

Figure S26. ^{13}C NMR spectrum (125.5 MHz, $CDCl_3$) of Compound 9

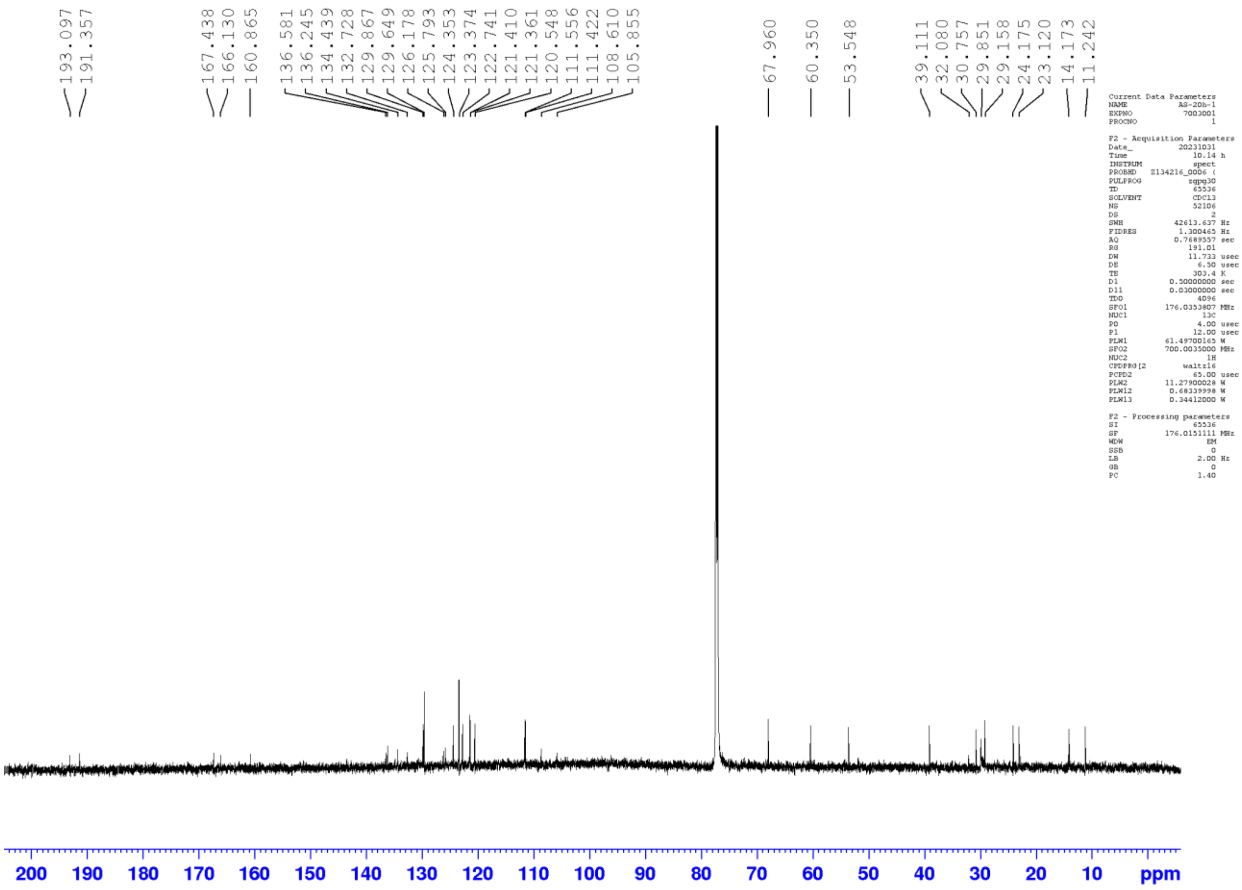

Figure S27. CD spectrum Compound 2

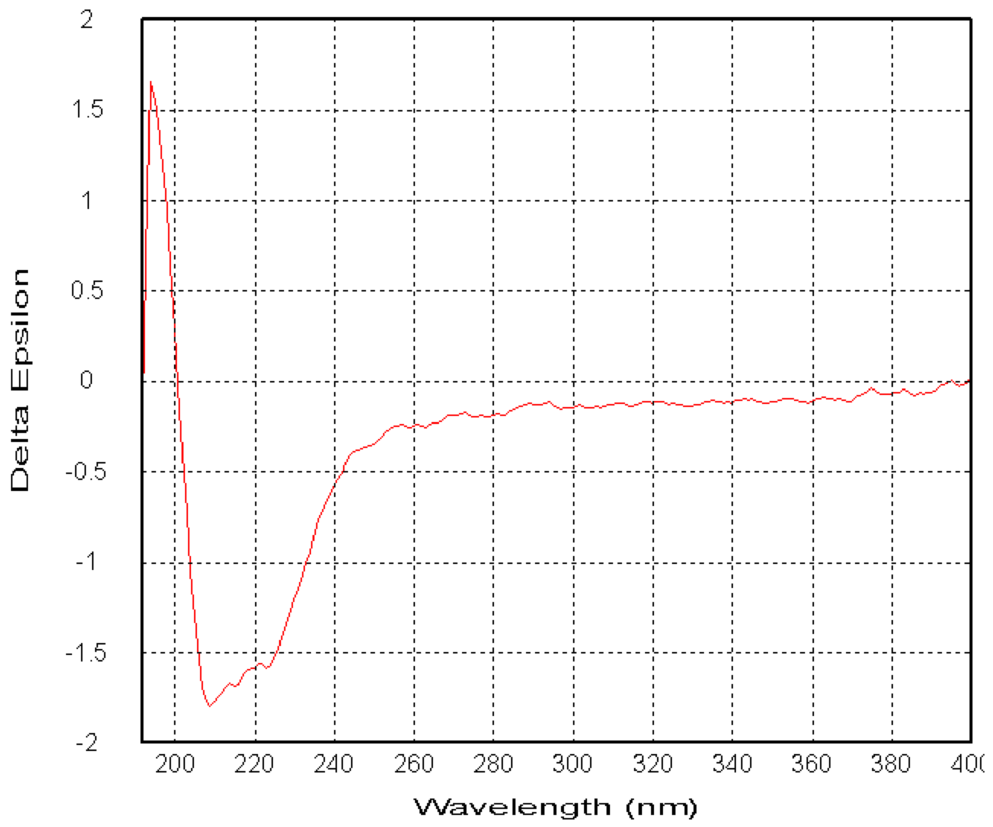

Figure S28. CD spectrum Compound 3

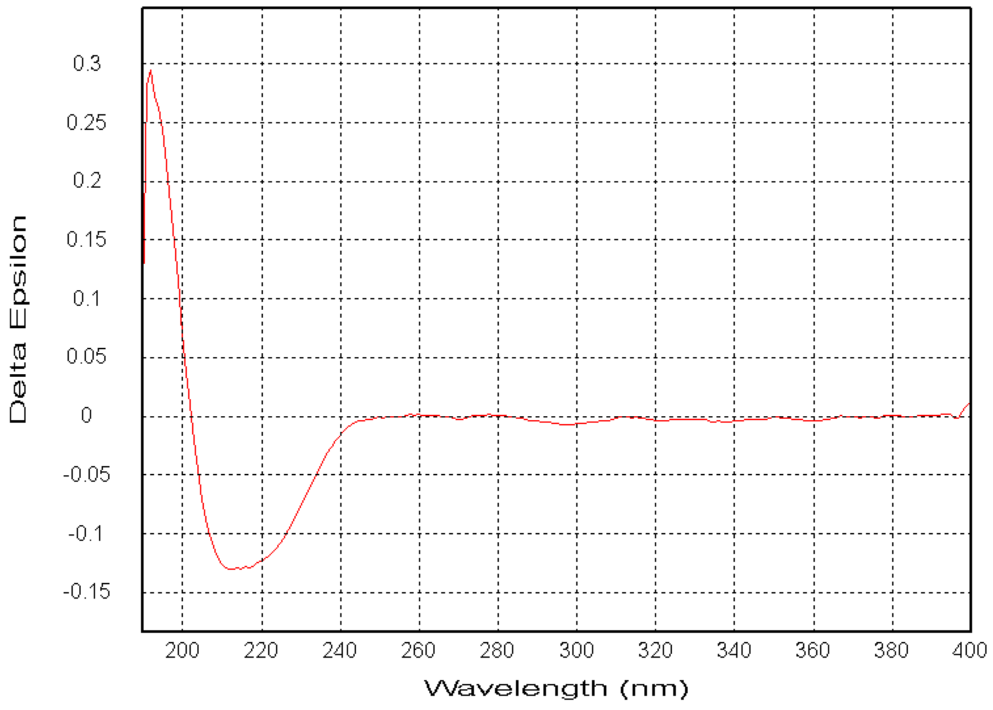

Figure S29. CD spectrum Compound 4

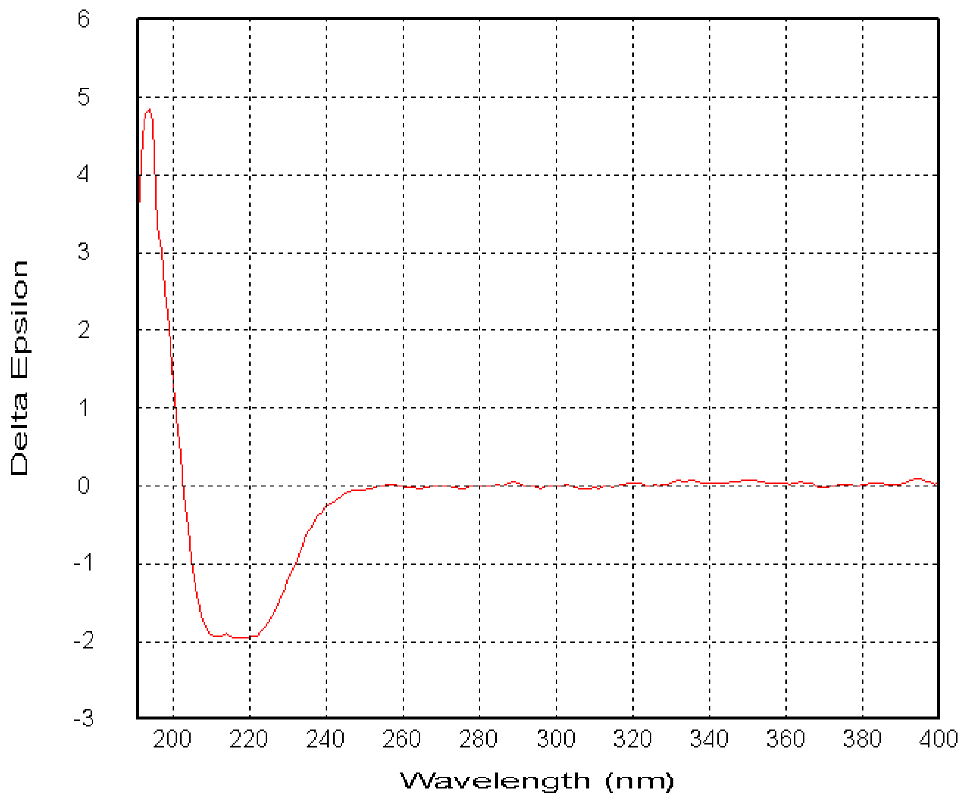

Figure S30. CD spectrum Compound 5

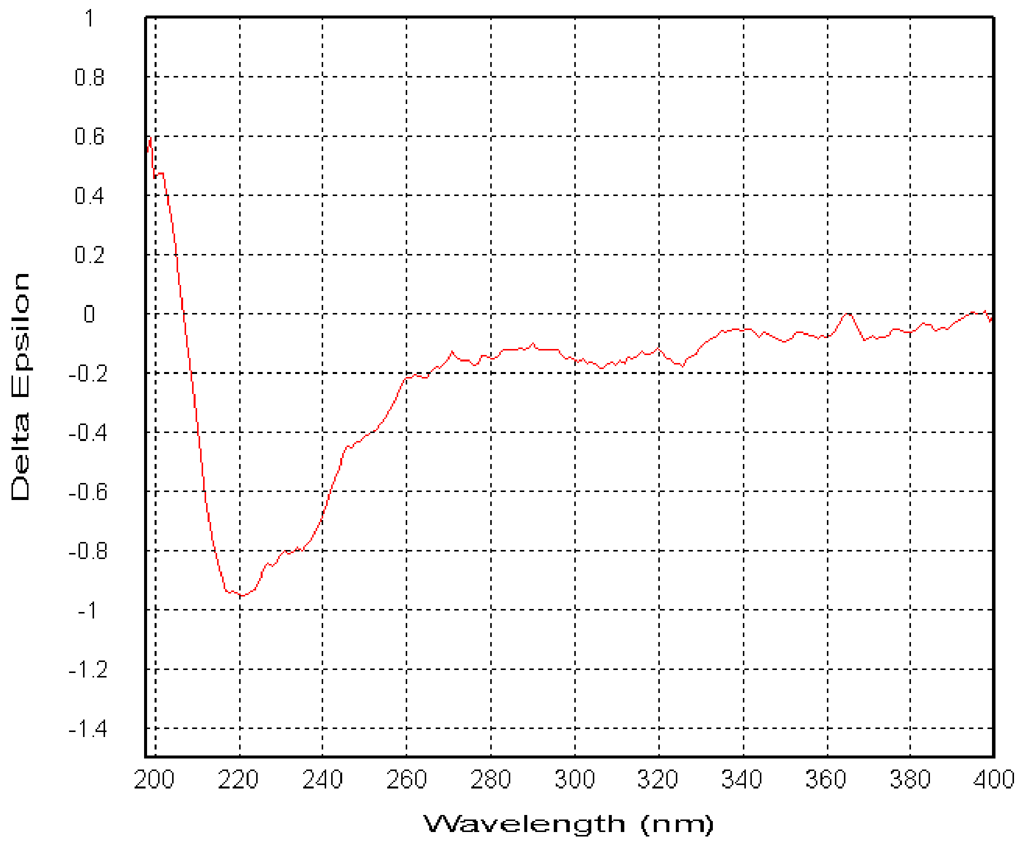

Figure S31. CD spectrum Compound 6

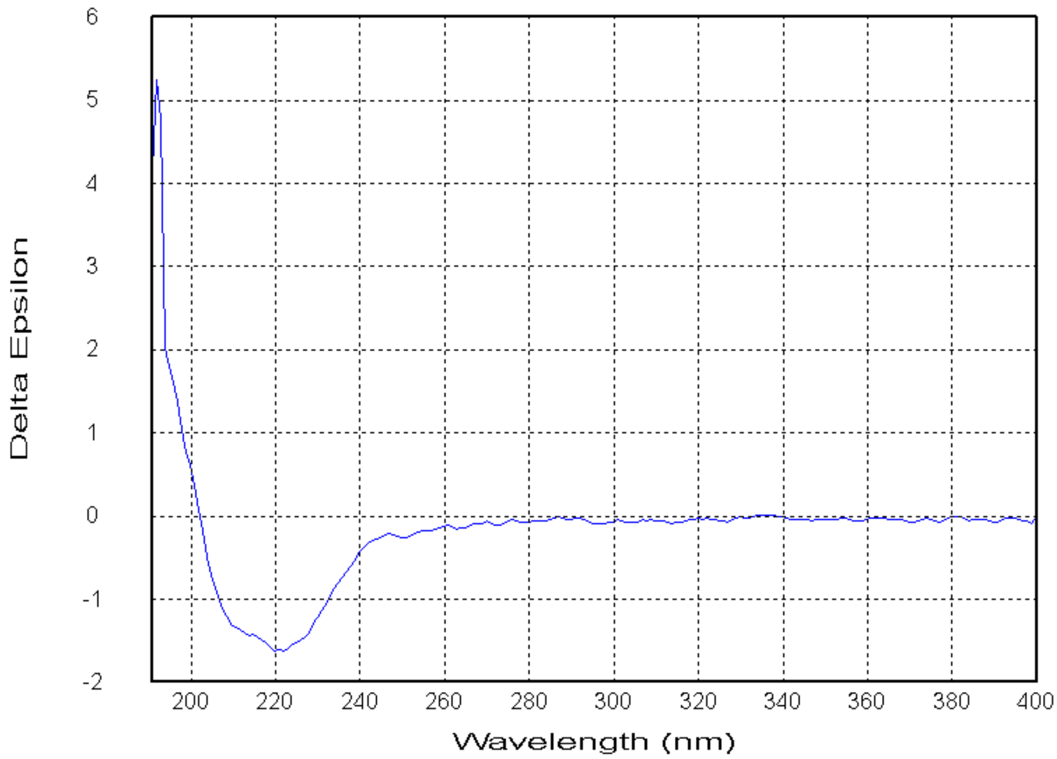

Figure S32. CD spectrum Compound 7

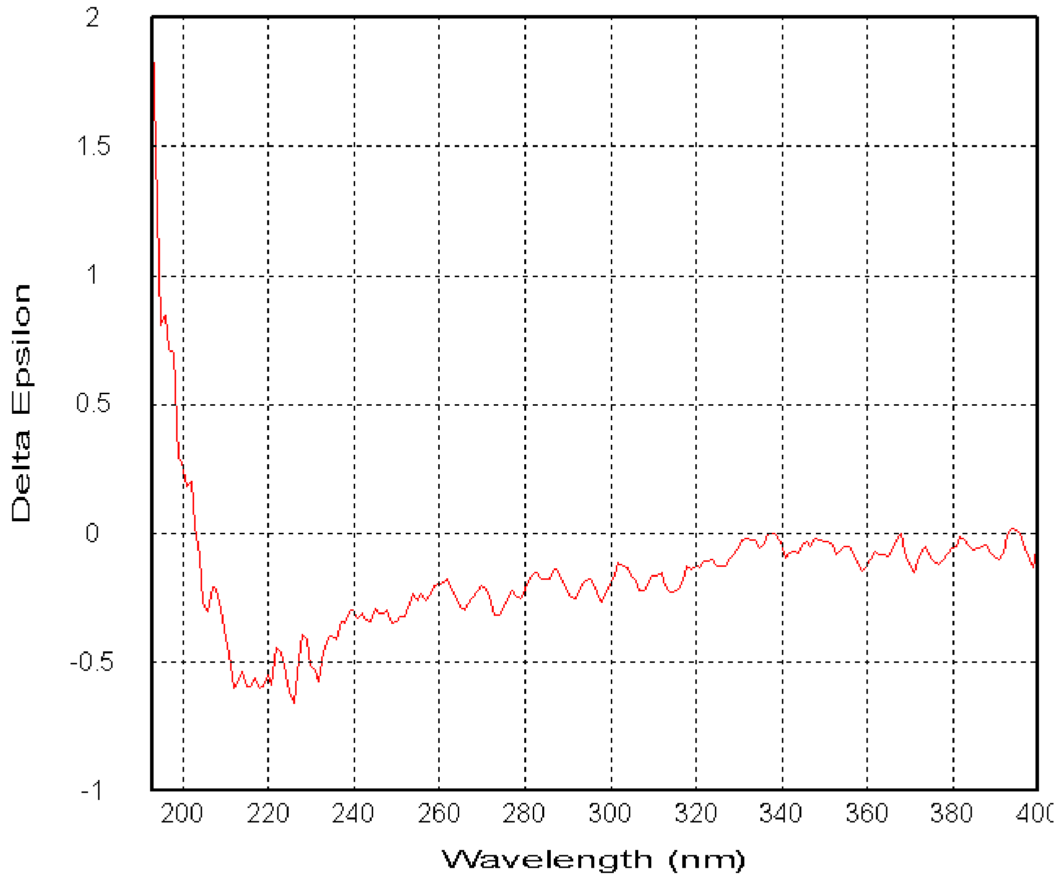

Figure S33. CD spectrum Compound 8

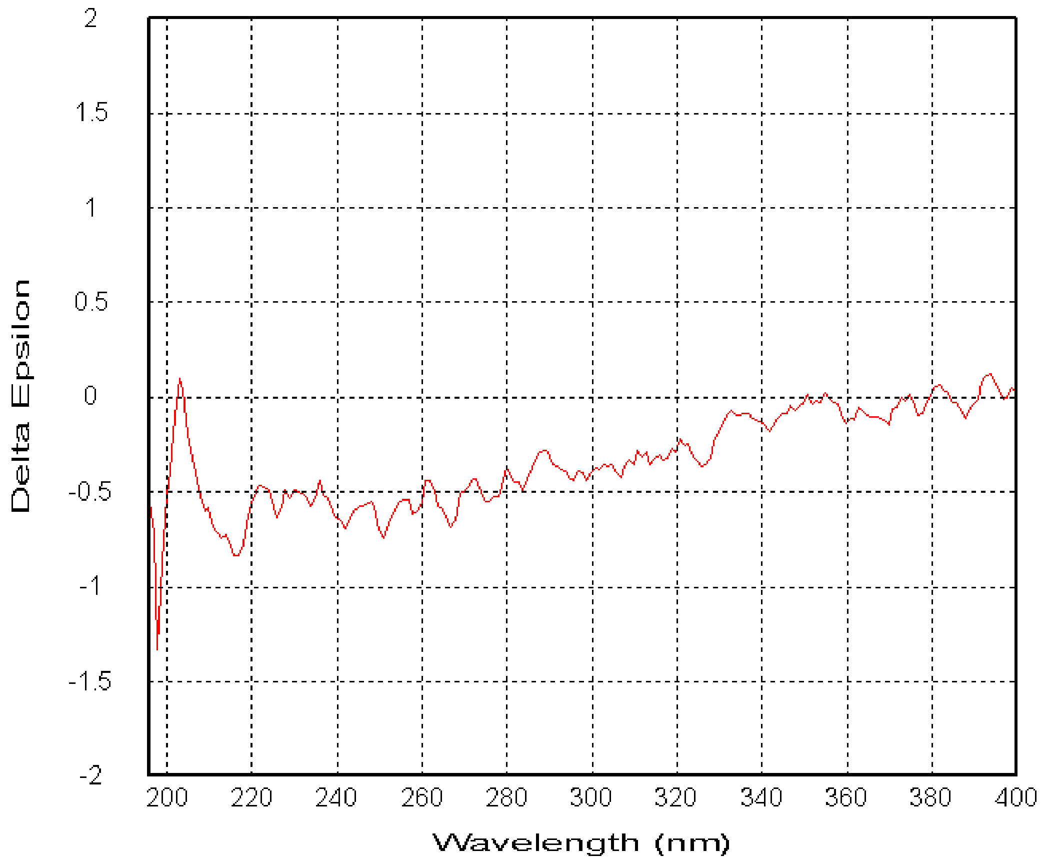

Figure S34. CD spectrum Compound 9

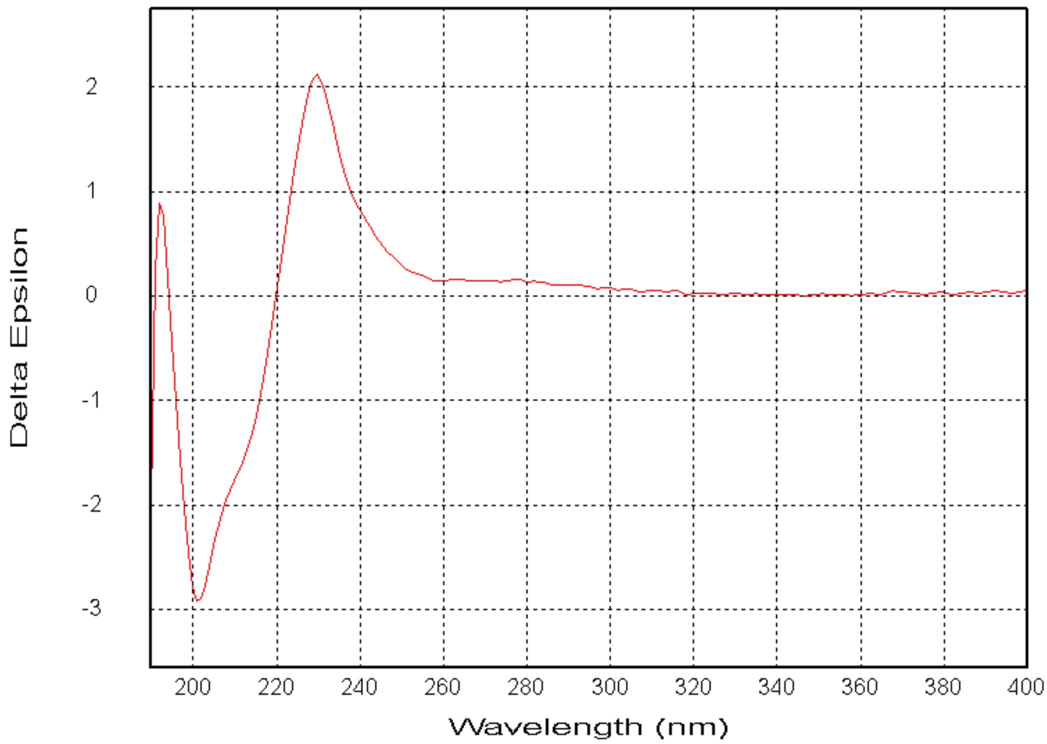

Figure S35. HR (+)ESI MS spectrum of 1

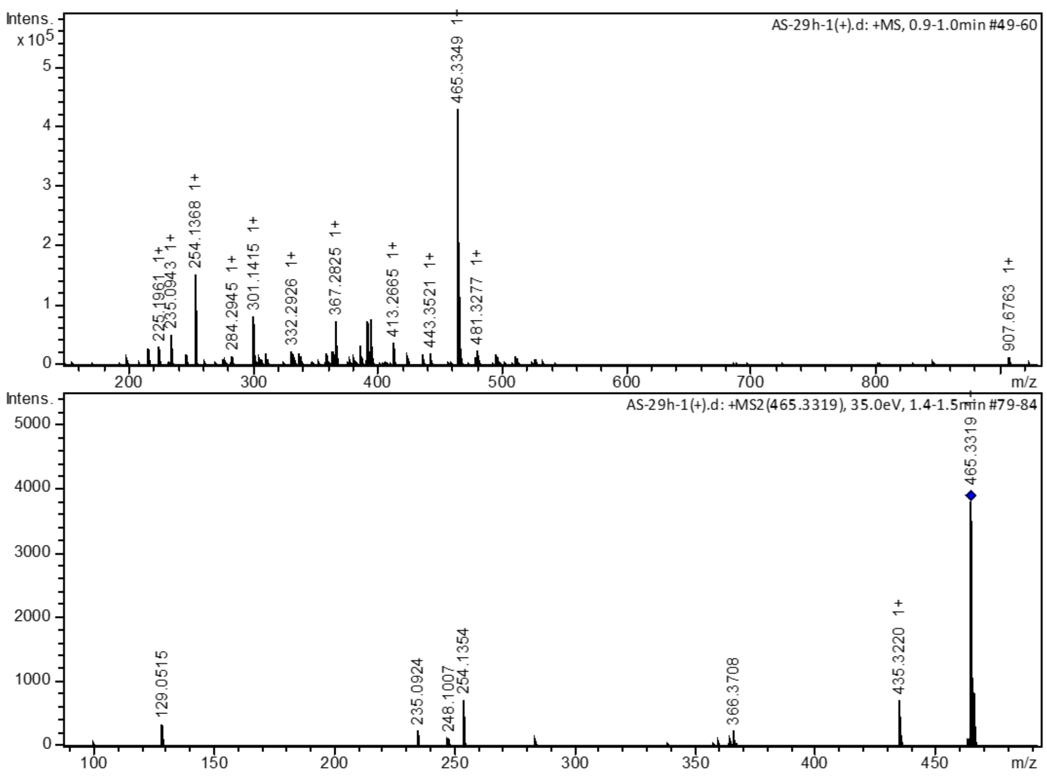

Figure S36. HR (+)ESI MS spectrum of 2

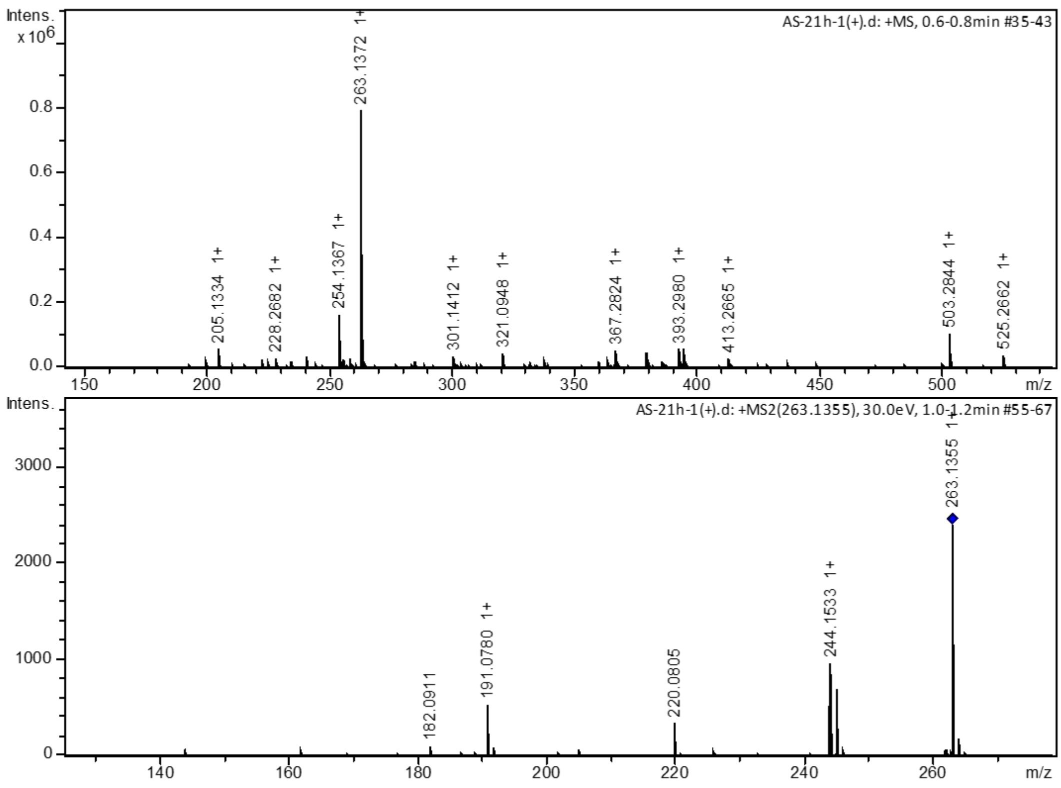

Figure S37. HR (+)ESI MS spectrum of 3

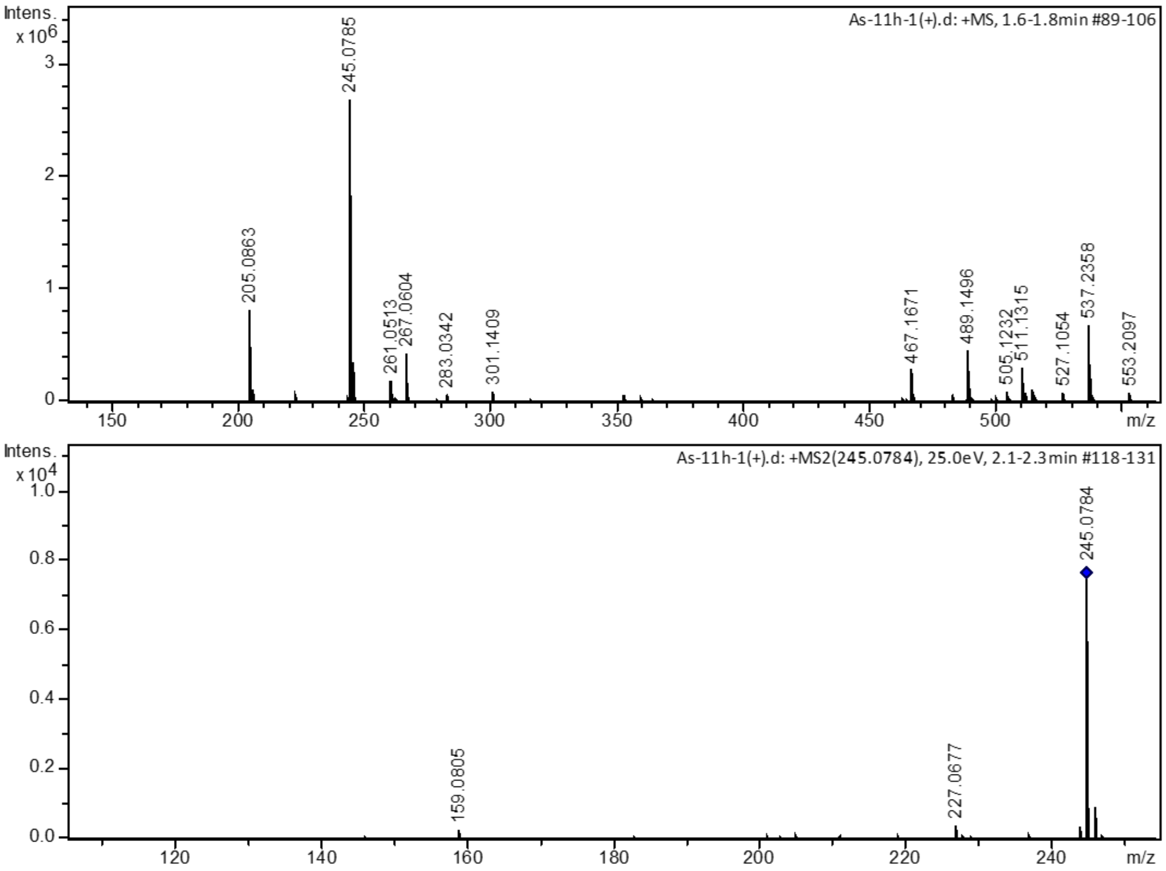

Figure S38. HR (+)ESI MS spectrum of 4

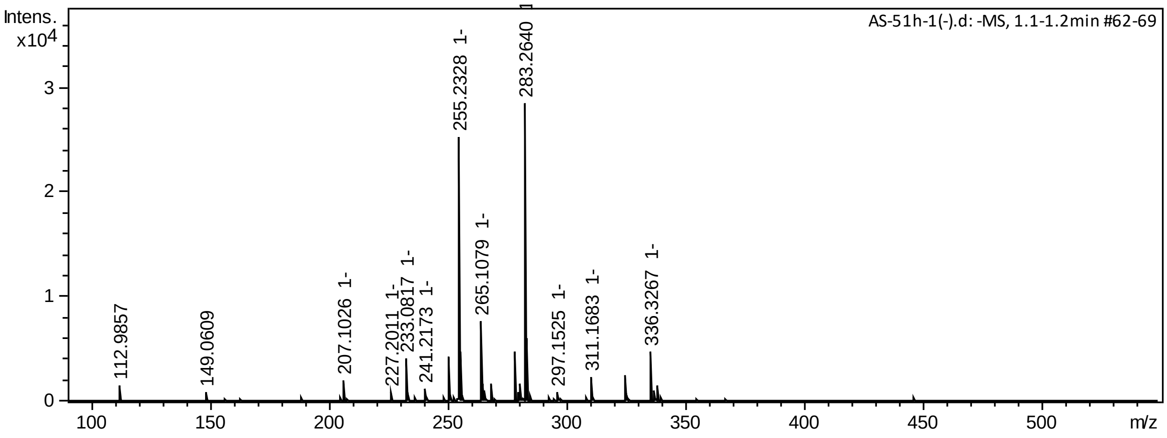

Figure S39. HR (+)ESI MS spectrum of 5

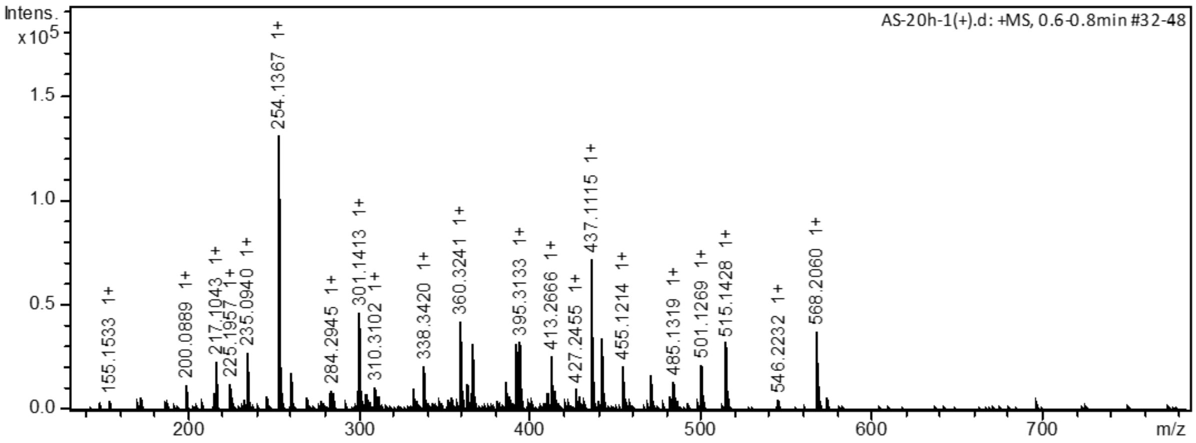

Figure S40. HR (+)ESI MS spectrum of 6

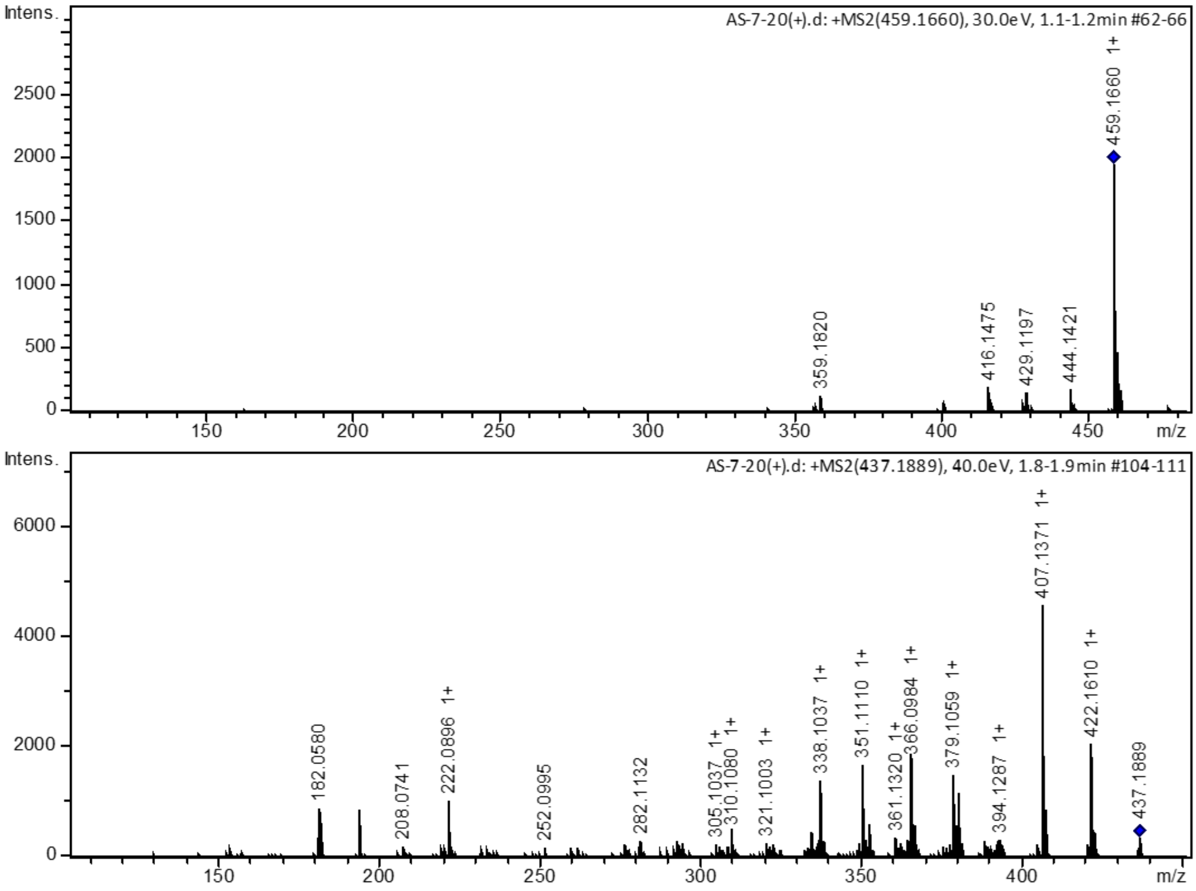

Figure S41. HR (+)ESI MS spectrum of 7

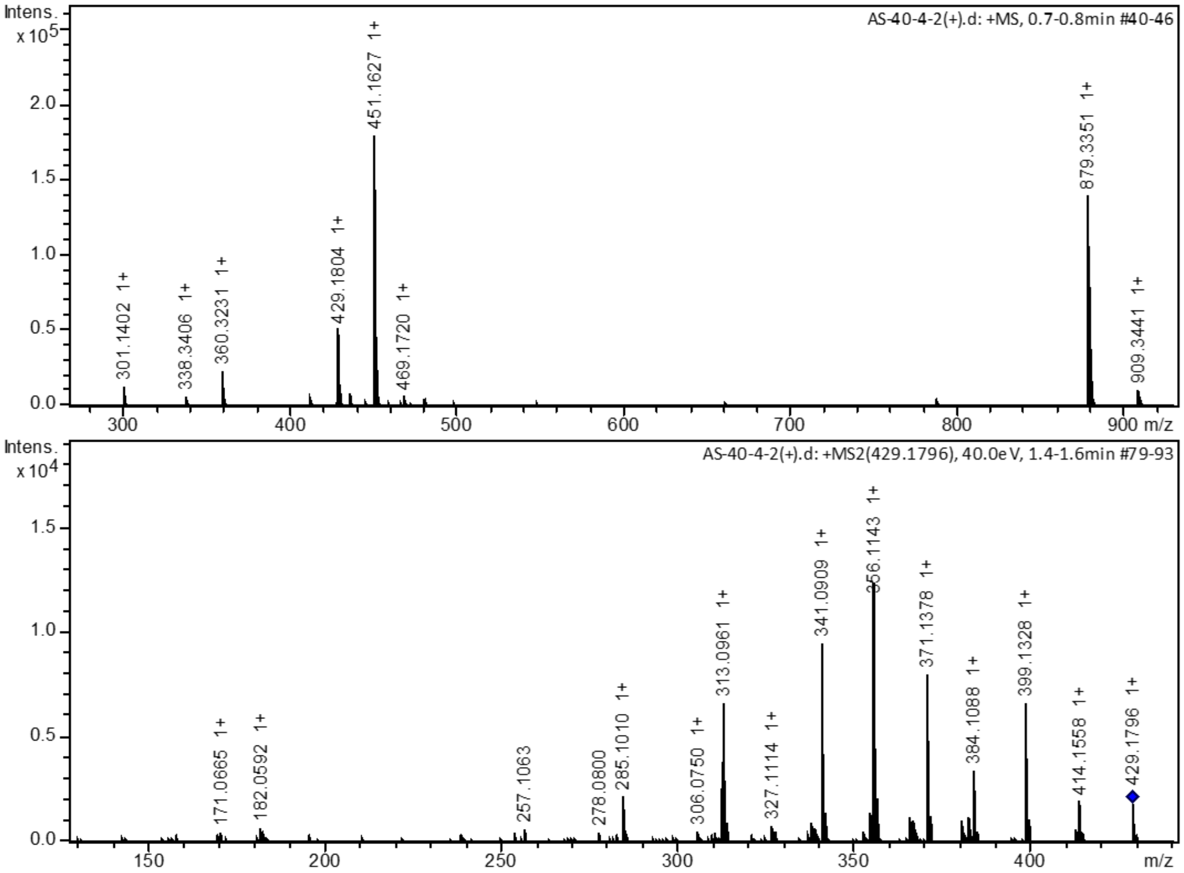

Figure S42. HR (+)ESI MS spectrum of 8

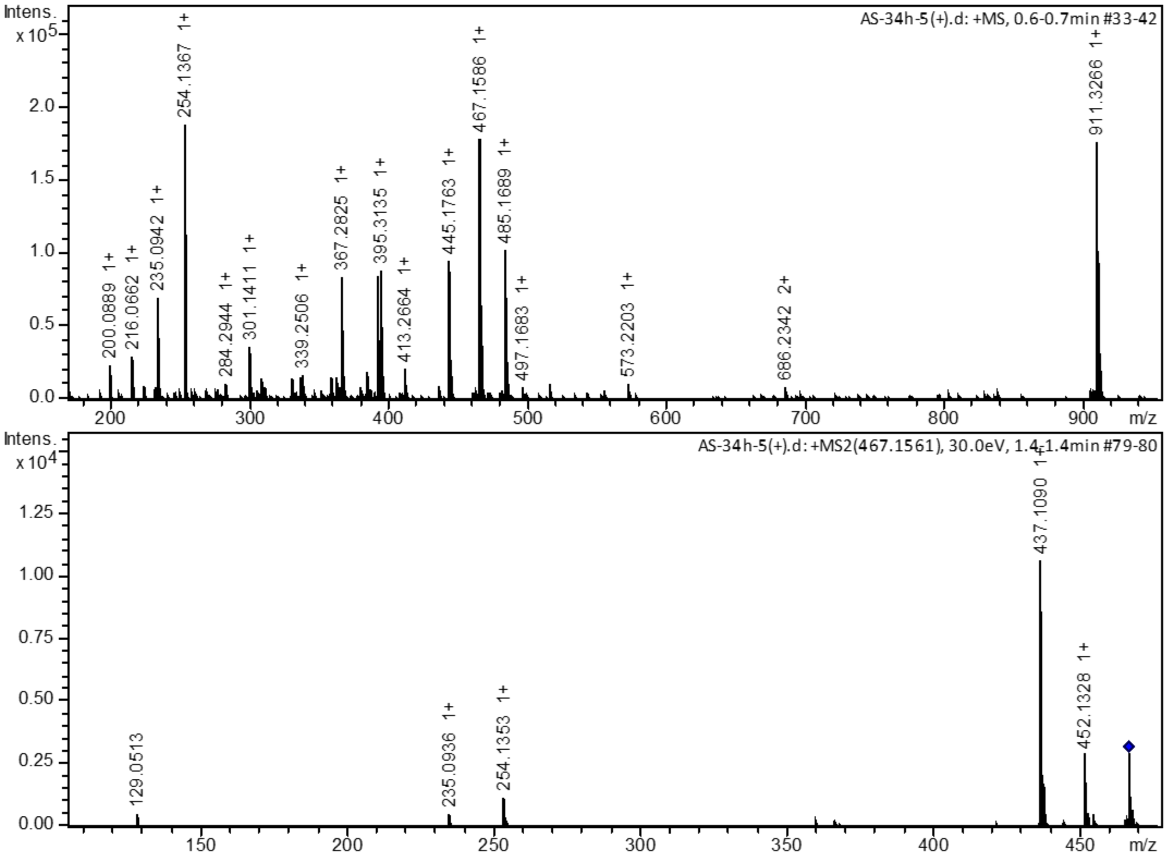

Figure S43. HR (-)ESI MS spectrum of 9

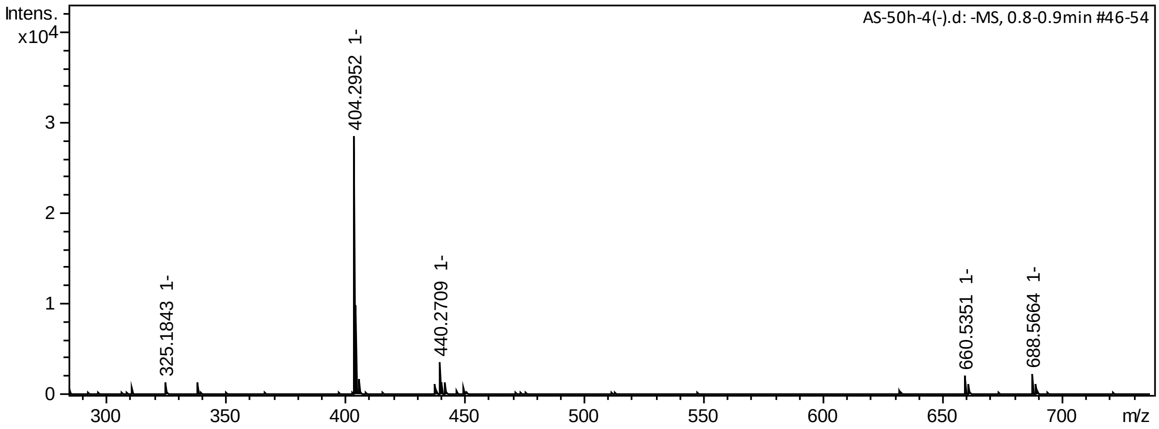

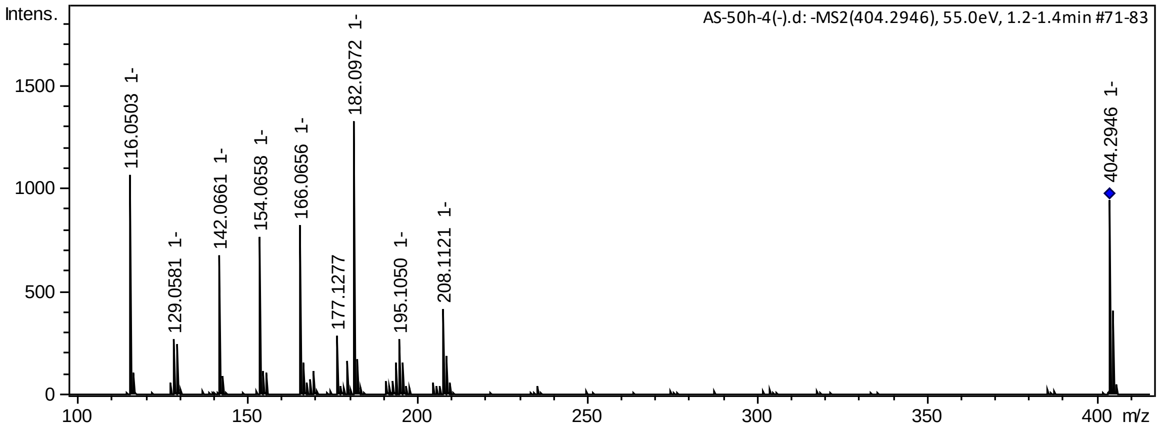

Figure S44. The main conformations of 7*S*,11*S*- and 7*S*,11*R*- stereoisomers of **2**

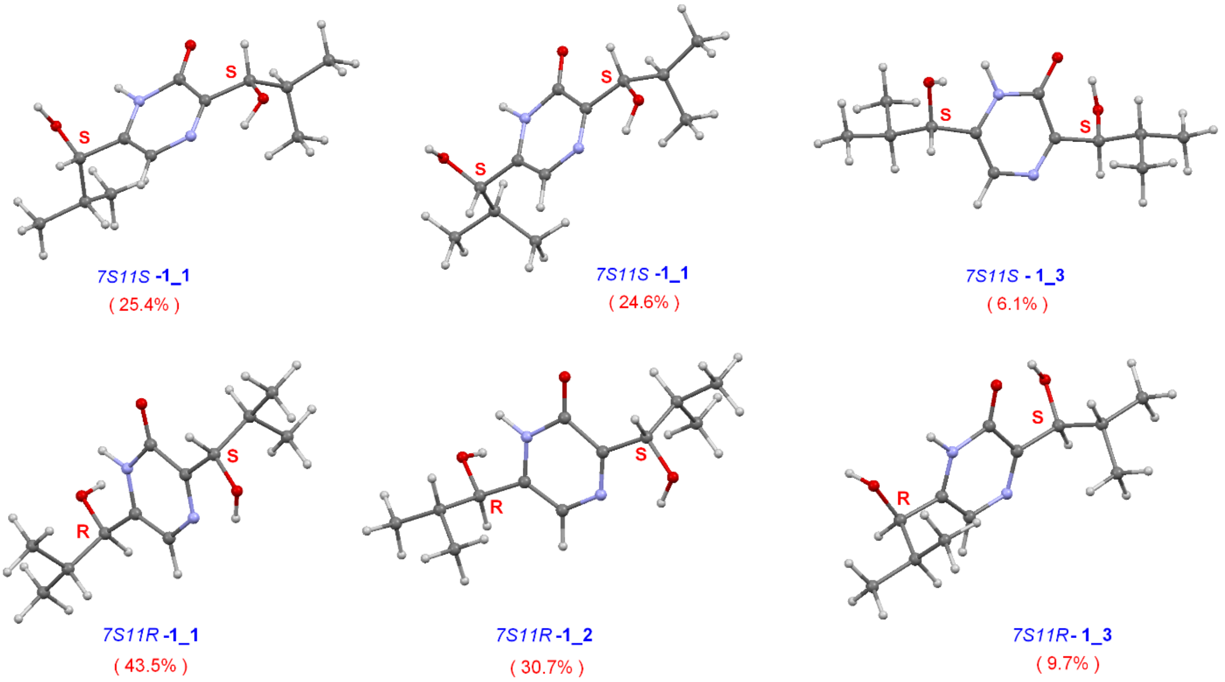

Figure S45. The potential energy profile for internal rotation of the 3-O–H group around C(3)–OH bond for compound 3.

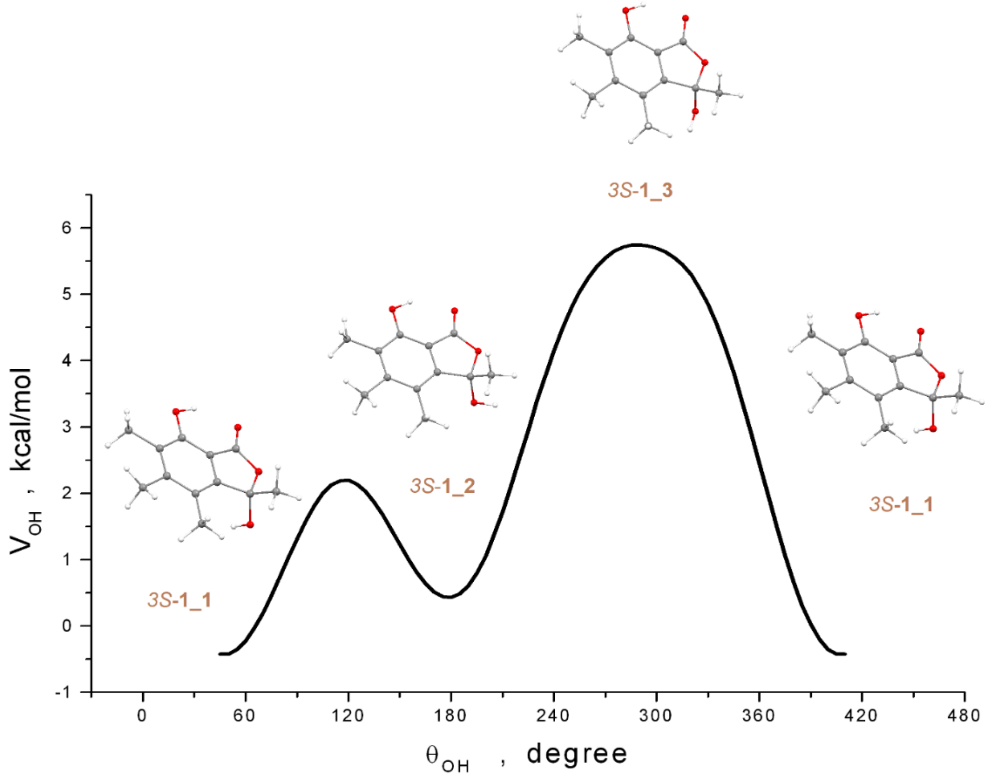

Figure S46. The ECD spectra, calculated for 3*S* stereoisomer of compound 3 as a function of different large-amplitude motions.

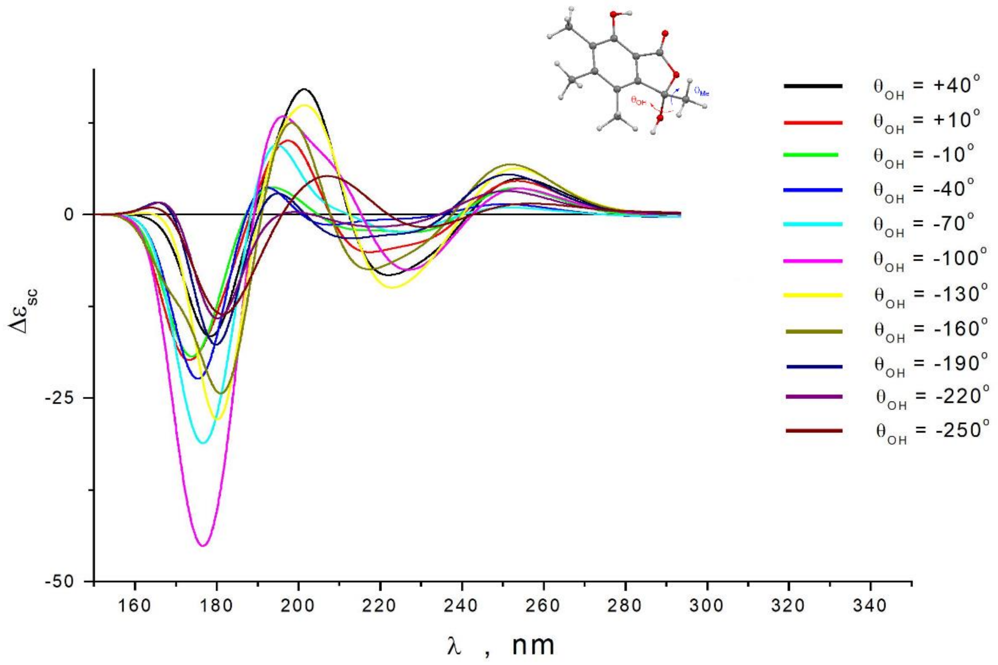

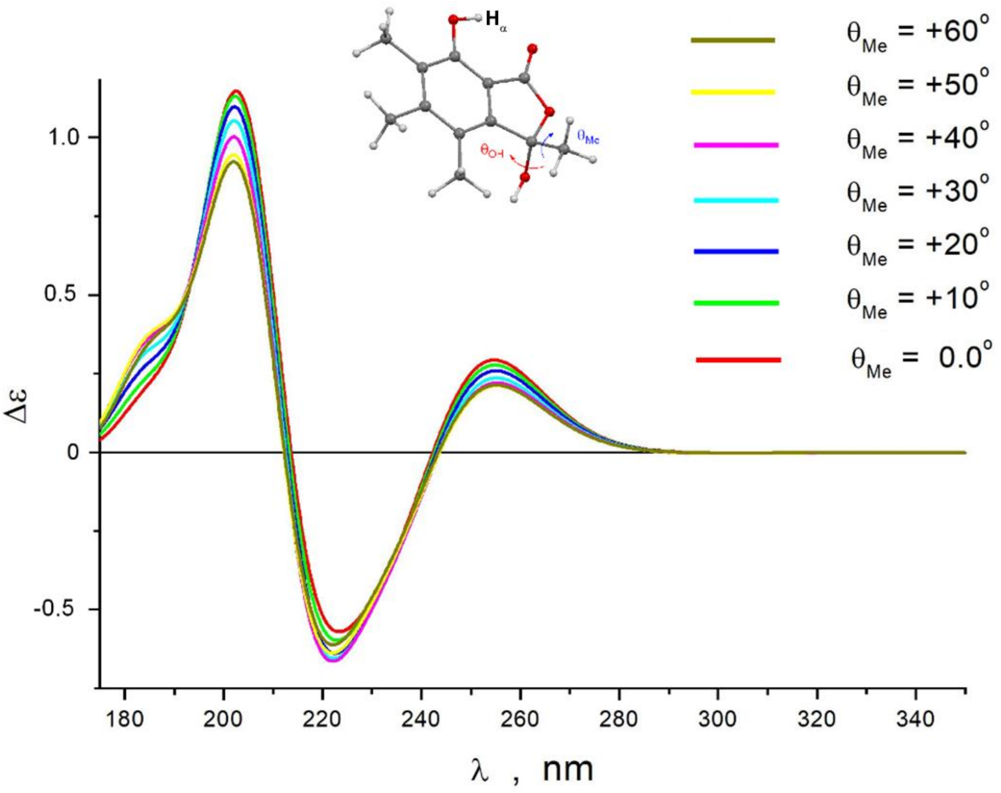

Figure S47. ECD spectra (Δε) and potential energy function (V) for the internal rotation of OH_α - group in 3*S* stereoisomer of compound 3.

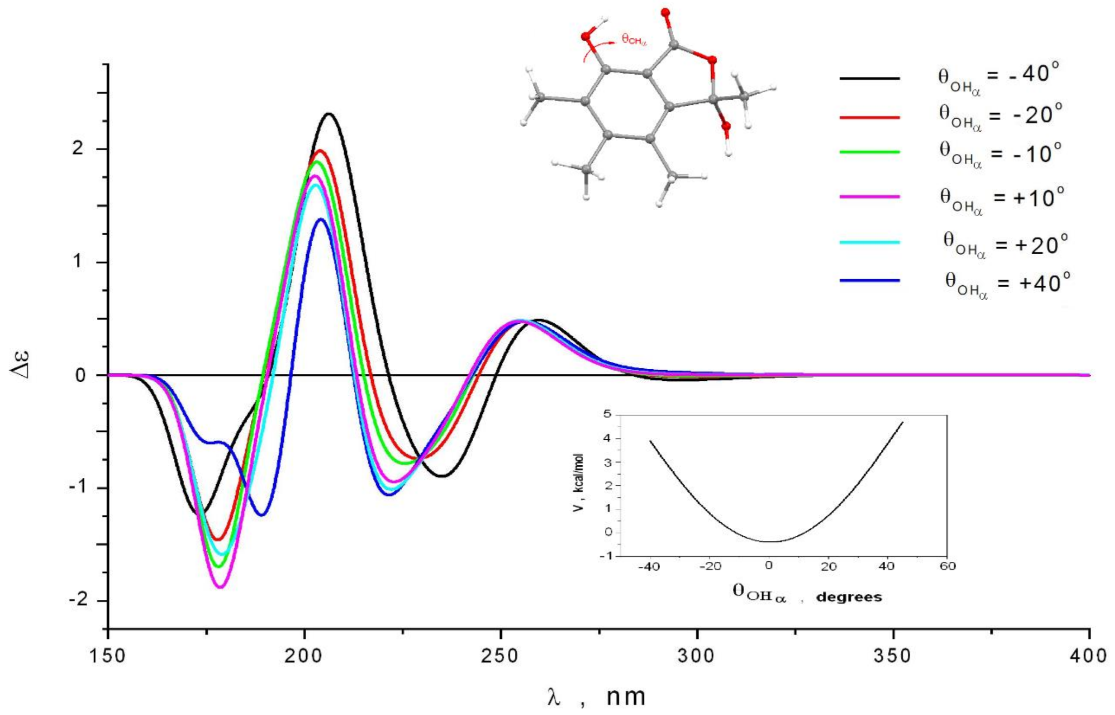

Figure S48. The calculated basis set dependence of ECD spectra of 3S stereoisomer of compound 3.

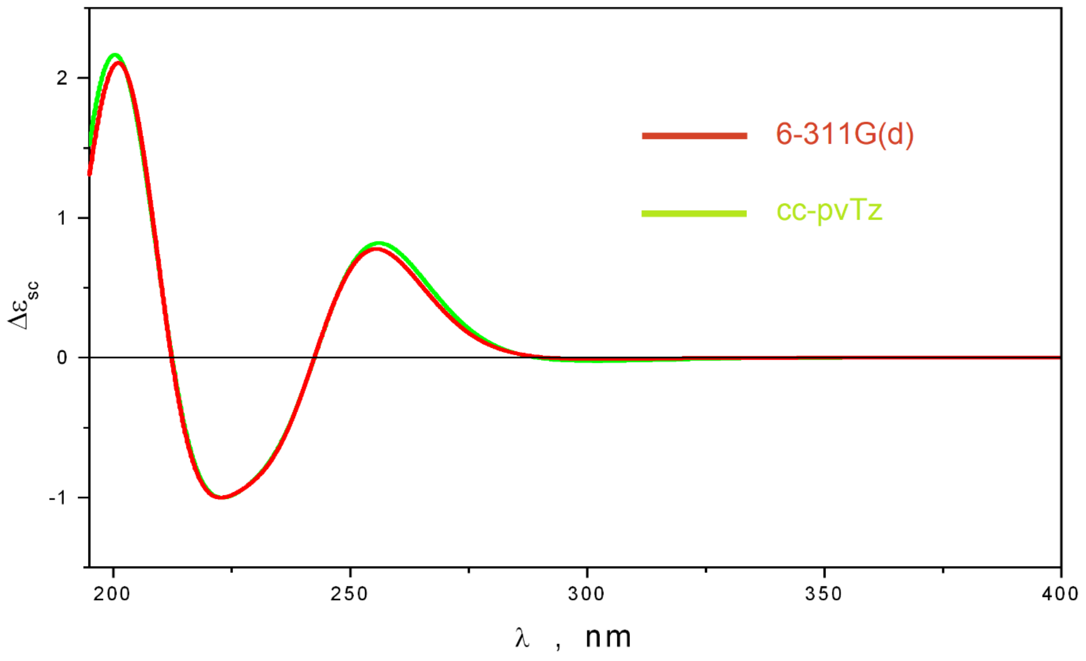

Supplement: Supplementary file 1 [file marinedrugs-23-00353-s001.zip › marinedrugs-3844360-supplementary.pdf]
